# Cu-Catalyzed aromatic C–H imidation with *N*-fluorobenzenesulfonimide: mechanistic details and predictive models†
†Electronic supplementary information (ESI) available: (1) Cu^I^Br oxidation by NFSI; (2) details of the Br/F exchange process; (3) bimetallic oxidation of LCu^I^X (where X = F, Br, Cl, and I) by NFSI; (4) conformational analysis of the oxidation of **D3-N-3F**; (5) NBO analysis of the Cu_2_F_2_ dimer; (6) analysis of electronic states along the catalytic cycle; (7) energy scan for the deprotonation step; (8) isotope effect calculation; (9) calculation procedure to predict regioselectivity for C–H imidation; (10) kinetic profiles of product formation of pre-catalysts; (11) characterization data, ^1^H and ^13^C NMR spectra; (12) observation of the proposed intermediate **D3-N-3F**; (13) energies and Cartesian coordinates. See DOI: 10.1039/c6sc04145k



**DOI:** 10.1039/c6sc04145k

**Published:** 2016-10-19

**Authors:** Brandon E. Haines, Takahiro Kawakami, Keiko Kuwata, Kei Murakami, Kenichiro Itami, Djamaladdin G. Musaev

**Affiliations:** a Cherry L. Emerson Center for Scientific Computation , Department of Chemistry , Emory University , Atlanta , Georgia 30322 , USA . Email: dmusaev@emory.edu; b Institute of Transformative Bio-Molecules (WPI-ITbM) and Graduate School of Science , Nagoya University , Chikusa , Nagoya 464-8602 , Japan; c JST-ERATO , Itami Molecular Nanocarbon Project , Nagoya University , Chikusa , Nagoya 464-8602 , Japan . Email: itami@chem.nagoya-u.ac.jp

## Abstract

The LCuBr-catalyzed C–H imidation of arenes by *N*-fluorobenzenesulfonimide (NFSI), previously reported by us, utilizes an inexpensive catalyst and is applicable to a broad scope of complex arenes. The computational and experimental study reported here shows that the mechanism of the reaction is comprised of two parts: (1) generation of the active dinuclear Cu^II^–Cu^II^ catalyst; and (2) the catalytic cycle for the C–H bond imidation of arenes. Computations show that the LCu^I^Br complex used in experiments is not an active catalyst. Instead, upon reacting with NFSI it converts to an active dinuclear Cu^II^–Cu^II^ catalyst that is detected using HRMS techniques. The catalytic cycle starting from the Cu^II^–Cu^II^ dinuclear complex proceeds *via* (a) one-electron oxidation of the active catalyst by NFSI to generate an imidyl radical and dinuclear Cu^II^–Cu^III^ intermediate, (b) turnover-limiting single-electron-transfer (**SET1**) from the arene to the imidyl radical, (c) fast C–N bond formation with an imidyl anion and an aryl radical cation, (d) reduction of the Cu^II^–Cu^III^ dinuclear intermediate by the aryl radical to regenerate the active catalyst and produce an aryl-cation intermediate, and (e) deprotonation and rearomatization of the arene ring to form the imidated product. The calculated KIE for the turnover-limiting **SET1** step reproduces its experimentally observed value. A simple predictive tool was developed and experimentally validated to determine the regiochemical outcome for a given substrate. We demonstrated that the pre-reaction LCuX complexes, where X = Cl, Br and I, show a similar reactivity pattern as these complexes convert to the same catalytically active dinuclear Cu^II^–Cu^II^ species.

## Abstract 2

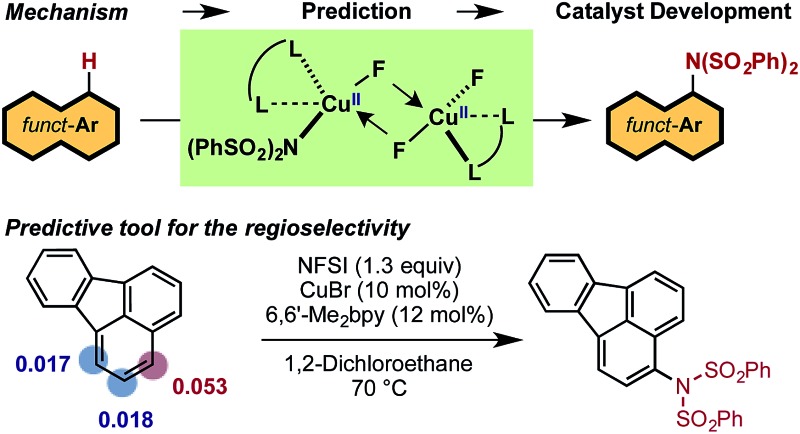
Dinuclear Cu^II^–Cu^II^ intermediate is an active catalyst for an unusual stepwise two-electron oxidation by NFSI, a regioselectivity predictive tool and a new catalyst development.

## Introduction

There exists a need for reliable catalytic methods capable of installing nitrogen functionalities onto a broad range of precious organic substrates. Of particular interest are arylamine functionalities (*i.e.*, aryl C–N bond formation) due to their privileged status in biomolecules^1^ and materials.^2,3^ Extensive investigations have established that conventional transition-metal-catalyzed methods (such as the Buchwald–Hartwig amination) can produce new aryl C–N bonds by coupling aryl halides with several types of amines.^4–10^ However, these approaches require pre-functionalization of the halide substrates. Therefore, the search for synthetic methods to directly transform C–H bonds into C–N bonds is of the utmost importance.

Indeed, the use of C–H amination^11–16^ methods allow for direct access to the desired C–N functionality in a vast range of hydrocarbons and are cost-effective and environmentally friendly.^17–19^ A pertinent example is the recent breakthrough by Nicewicz and coworkers utilizing organic photoredox catalysis to functionalize arene substrates with N-heterocycles and ammonium salts.^20^ Transition metal catalyzed aryl C–H amination is another highly attractive approach, but it suffers from limitations such as the need for a directing group,^21–25^ excess of arene substrate,^26,27^ and limited substrate scope.^28,29^

**Fig. 1:**
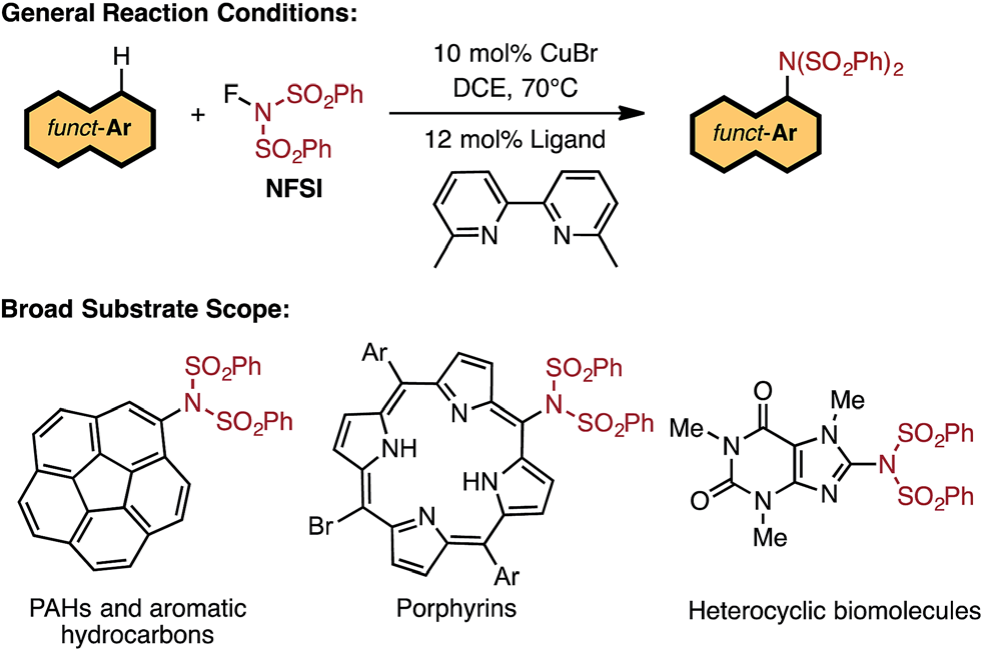
LCu^I^Br-catalyzed aromatic C–H imidation with NFSI that is applicable to a broad range of functional aromatic molecules.^33^

Recent studies by Baran and coworkers^30^ and Ritter and coworkers^31,32^ explicitly address these limitations by creating new C–N bonds with ferrocene and Pd catalysts, respectively, without the need of a directing group or excess aromatic substrate. Along the same lines, Cu-catalyzed aromatic C–H imidation with *N*-fluorobenzenesulfonimide (NFSI), reported by Itami and coworkers,^33^ not only utilizes an earth-abundant transition metal complex (CuBr) and a commonly available bipyridine ligand, 6,6′-dimethyl-2,2′-bipyridine (6,6′-Me_2_bpy), but is also applicable to a broad scope of complex arenes relevant to bio-functional and materials systems (Fig. 1). Intriguingly, the reactivity of the Cu catalyst shows a strong dependence on the 6,6′-substitution of the bpy ligand with the highest yields achieved using 6,6′-Me_2_bpy.^33^ As such, an atomistic level understanding of the mechanism of this important reaction will greatly advance the burgeoning field of aromatic C–H amination.

A number of previously reported experiments provide some insight into the mechanism of this reaction.^33^ Namely, independent deuterium labeling studies with 2-phenylthiophene (**1**) and 2-deuterio-5-phenylthiophene (**1-D**) show an inverse kinetic isotope effect (KIE) of *k*_H_/*k*_D_ = 0.91 at 70 °C,^33–37^ which implies addition to the arene in the rate-limiting step.^33^ This finding is instrumental in understanding why the electronic nature of the arene substrate is important to the observed activity of the catalyst: (a) arenes with extended π-systems and electron-rich heterocycles react nicely, but simple aromatics such as benzene produce low yields; and (b) electron-rich arenes react faster than electron-deficient arenes in competition experiments.^33^ It also should be noted that this reaction, as well as that reported by Ritter and coworkers,^31,32^ proceeds with unusually high levels of regioselectivity with a broad range of substrates. Ritter and coworkers^32^ attribute this to significant charge transfer from the arene to the highly electrophilic nitrogen radical derived from Selectfluor in the C–N bond-forming step. Lectka and coworkers also describe importance of the electronic effect in Cu-catalyzed C(sp^3^)–H fluorination with Selectfluor.^38^ It remains to be seen if this is also the case for the NFSI oxidant.

In general, NFSI is a two-electron oxidant that belongs to a class of electrophilic fluorinating reagents^39^ used as an equivalent of the highly oxidizing fluoronium cation (“F^+^”).^40–43^ For example, as shown by Muñiz, NFSI reacts with Pd^II^ as a two-electron oxidant to form a Pd^IV^ intermediate.^44^ However, NFSI is a versatile reagent that is known to also react in one-electron processes such as radical fluorination (F˙) of alkyl radicals.^45–49^ Therefore, a better understanding of how NFSI reacts with earth-abundant transition metals complexes such as the LCuBr is highly desirable.

**Fig. 2:**
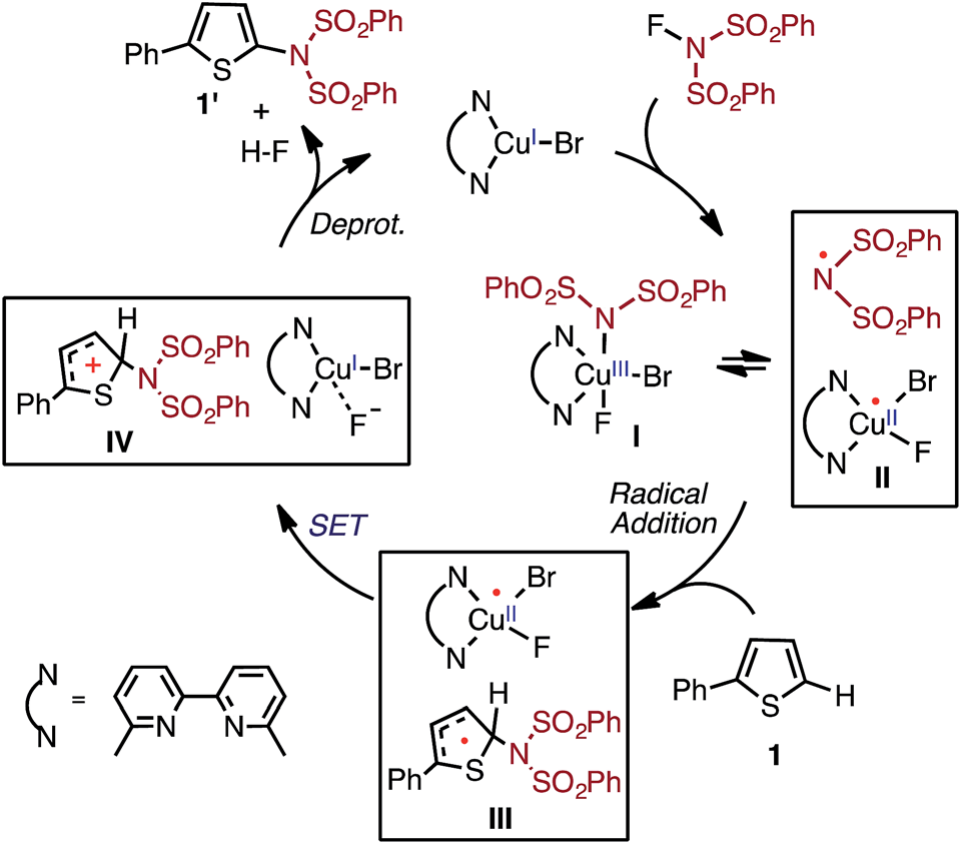
Proposed catalytic cycle for LCu^I^Br-catalyzed aromatic C–H imidation with NFSI.^33,54^

Based on the above-mentioned findings,^31–33,50–54^ previously we proposed the following multi-step mechanism for the LCu^I^Br catalyzed aromatic C–H imidation (where L = 6,6′-Me_2_bpy) by NFSI (Fig. 2): (a) oxidation of LCu^I^Br by NFSI to form LCu^III^BrF(NSI), (**I**), [where NSI = N(SO_2_Ph)_2_] in equilibrium with LCu^II^BrF and imidyl radical (**II**);^54^ (b) rate-limiting imidyl radical addition to the arene substrate (C–N bond formation) to form aryl radical (**III**); (c) single electron transfer (SET) from the aryl radical to LCu^II^BrF to regenerate the LCu^I^Br catalyst and produce an aryl cation (**IV**) and coordinated F^–^, and (d) deprotonation and rearomatization of the aryl cation by F^–^ to afford the imidated arene product and HF.

However, this proposed mechanism lacks the intimate details of the reaction that are essential to rationally design more effective catalysts for selective C–H imidation and to predict the regioselective outcome. Therefore, here we set out to gain an in-depth understanding of the mechanism and governing factors of Cu-catalyzed aromatic C–H imidation by NFSI by means of a computational and experimental collaboration. This study provides insight into: (a) the versatile reactivity of NFSI; (b) the true nature of the active Cu catalyst; and (c) the impact of the nature of arene substrate on the observed reactivity and selectivity. The acquired fundamental knowledge will be used to develop the next generation of novel catalysts and ligands for selective C–H imidation with broader scope and higher reactivity.

## Results and discussion

Geometry optimizations and frequency calculations of all presented structures were performed at the B3LYP-D3/[6-31G(d,p) + Lanl2dz (Cu, Br, I)] level of theory (B3LYP-D3/BS1). The reported energies were re-computed at the B3LYP-D3/[6-311+G(d,p) + SDD (Cu, Br, and I)] level of theory (B3LYP-D3/BS2). The calculated Gibbs free energies are corrected to a solution standard state of 1 M at 298.15 K.^55,56^ Bulk solvent effects are incorporated by using the IEF-PCM method^57–59^ with 1,2-dichloroethane (DCE) as the solvent.

The structures discussed in the text are labeled as **(D)A-X-Y-Z**, where **A** indicates the position of the structure on the free energy surface (*e.g.*, **A** = **1**, **TS**, *etc.*), the **D** prefix is appended when the structure is a dinuclear Cu complex, and **X**, **Y**, and **Z** explicitly identify the ligands directly attached to the Cu center(s). This notation clearly shows how the ligand environment of Cu changes as the reaction progresses (see Notes for details of the used computational procedure. Also, see the ESI† for Cartesian coordinates of all reported structures).

### *In situ* generation of active catalyst

**Fig. 3:**
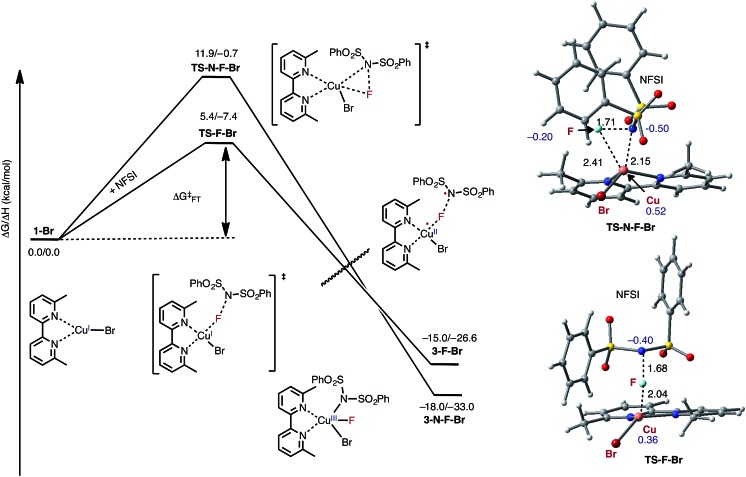
Free energy profile for oxidation of LCu^I^Br through the oxidative addition (**TS-N-F-Br**) and F-atom transfer pathways (**TS-F-Br**). Bond distances (in Å) are shown in black and Mulliken spin density values (in |*e*|) are shown in blue. For simplicity, only the pathways through the lowest energy electronic states are shown (see the ESI† for more details).

As indicated in Fig. 2, the first step of the reaction is oxidation of the LCu^I^Br (where L = 6,6′-Me_2_bpy) complex (**1-Br**) by NFSI (see the ESI† for analysis of full potential energy surface of the reaction). In general, this process may proceed *via* two distinct pathways: (a) NFSI as a two-electron oxidant,^54^ *i.e.* oxidative addition of the N–F bond to the Cu^I^-center of LCu^I^Br *via* transition state **TS-N-F-Br** to form Cu^III^ intermediate LCu^III^BrF[NSI] (**3-N-F-Br**) or (b) NFSI as an one-electron oxidant, *i.e.* one-electron oxidation of LCu^I^Br by F-atom transfer^44,60^ (at the transition state **TS-F-Br**) to form the Cu^II^ intermediate LCu^II^BrF···[NSI], (**3-F-Br**) (see Fig. 3).

**Fig. 4:**
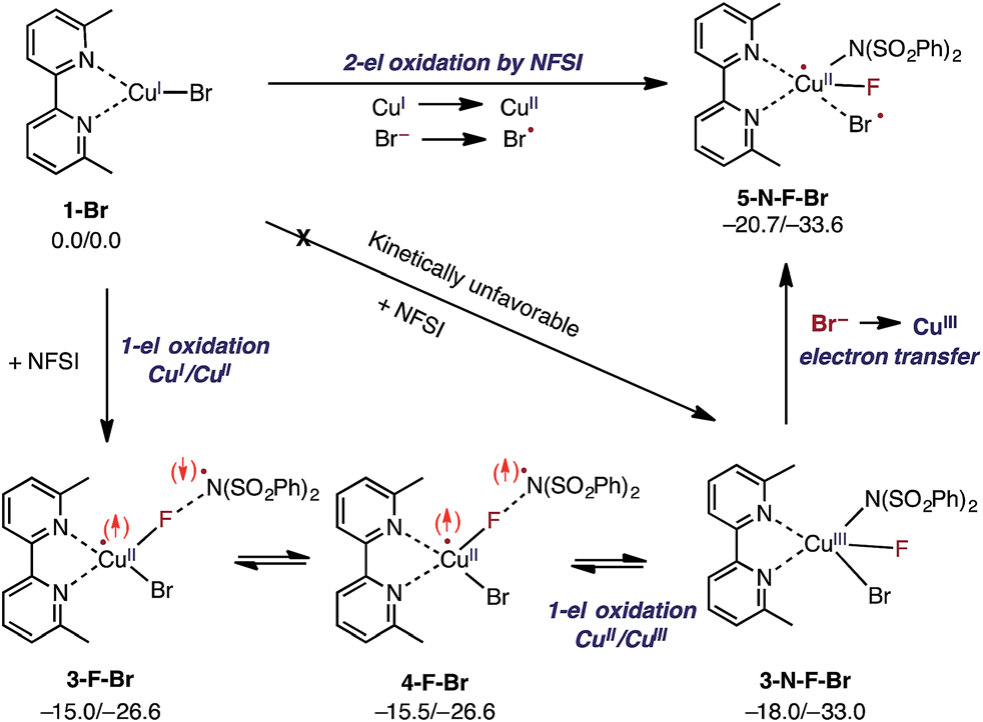
Mechanism for initial two-electron reduction of NFSI by LCu^I^Br (**1-Br**), which results in one-electron oxidation of the mono-nuclear Cu^I^ complex and a one-electron oxidation of the bromide ligand to a bromine radical. The signs of the explicitly depicted spins were assigned arbitrarily. Relative energies are given as Δ*G*/Δ*H* in kcal mol^–1^.

Extensive calculations show that the one-electron oxidation transition state **TS-F-Br** has a significant antiferromagnetic diradical character with 0.40|*e*| and 0.36|*e*| unpaired β- and α-spins on the nitrogen atom of the NSI fragment and Cu center, respectively. The free energy barrier (Δ*G*‡FT) associated with **TS-F-Br** is calculated to be 5.4 kcal mol^–1^ (calculated relative to **1-Br** + NFSI), which is 6.5 kcal mol^–1^ lower than that for oxidative addition through **TS-N-F-Br**.^61–65^ Thus, based on these findings, we can conclude that the reaction of LCu^I^Br with NFSI proceeds through the kinetically favored one-electron oxidation pathway that leads to the formation of antiferromagnetically coupled Cu^II^ intermediate **3-F-Br** (Fig. 3) with 0.71|*e*| and 0.67|*e*| unpaired β- and α-spins on the nitrogen atom of the NSI fragment and Cu center, respectively. This product is in equilibrium with its ferromagnetically-coupled counterpart **4-F-Br** (see Fig. 4). The imidyl fragment of **4-F-Br** has significant unpaired spin (0.74|*e*|) and is only weakly bound to the rest of the molecule (Cu–N = 3.51 Å).

Careful analyses indicated that complex **4-F-Br** easily rearranges through radical combination to the more stable two-electron oxidation product **3-N-F-Br**, which is Δ*G* = 2.5 kcal mol^–1^ lower in energy (see Fig. 4). However, the Cu^III^ complex **3-N-F-Br** is also metastable and converges to the energetically most stable complex **5-N-F-Br** (Δ*G* = –5.2 kcal mol^–1^ relative to **4-F-Br**) with a fully formed Cu–N bond (Cu–N = 2.35 Å) and a loosely associated Br radical (Cu–Br = 2.94 Å and the Br atom has 0.75|*e*| unpaired spin). Thus, the **3-N-F-Br** → **5-N-F-Br** redox transformation entails electron transfer from the coordinated bromide ligand to the Cu^III^ center to form a loosely associated bromine radical and a Cu^II^ center.

In summary, the presented calculations show that the reaction of LCu^I^Br with NFSI indeed results in a two-electron reduction of NFSI, which proceeds *via* (a) one-electron Cu^I^-to-Cu^II^ oxidation through an F-atom transfer process (**1-Br** + NFSI → **4-F-Br**) and (b) a series of one-electron redox steps leading to the formation of the most stable Cu^II^ intermediate **5-N-F-Br** with a loosely coordinated Br-radical (**4-F-Br** → **3-N-F-Br** → **5-N-F-Br**). In other words, the two-electron reduction of one molecule of NFSI by LCu^I^Br does not result in two-electron oxidation of the Cu center but instead produces **5-N-F-Br** with Cu^II^ and Br radicals (see Fig. 3 and 4). It is important to emphasize that the oxidation also does not lead to formation of the reactive imidyl radical (**4-F-Br**), which is endergonic by 5.2 kcal mol^–1^ relative to **5-N-F-Br**.

**Fig. 5:**
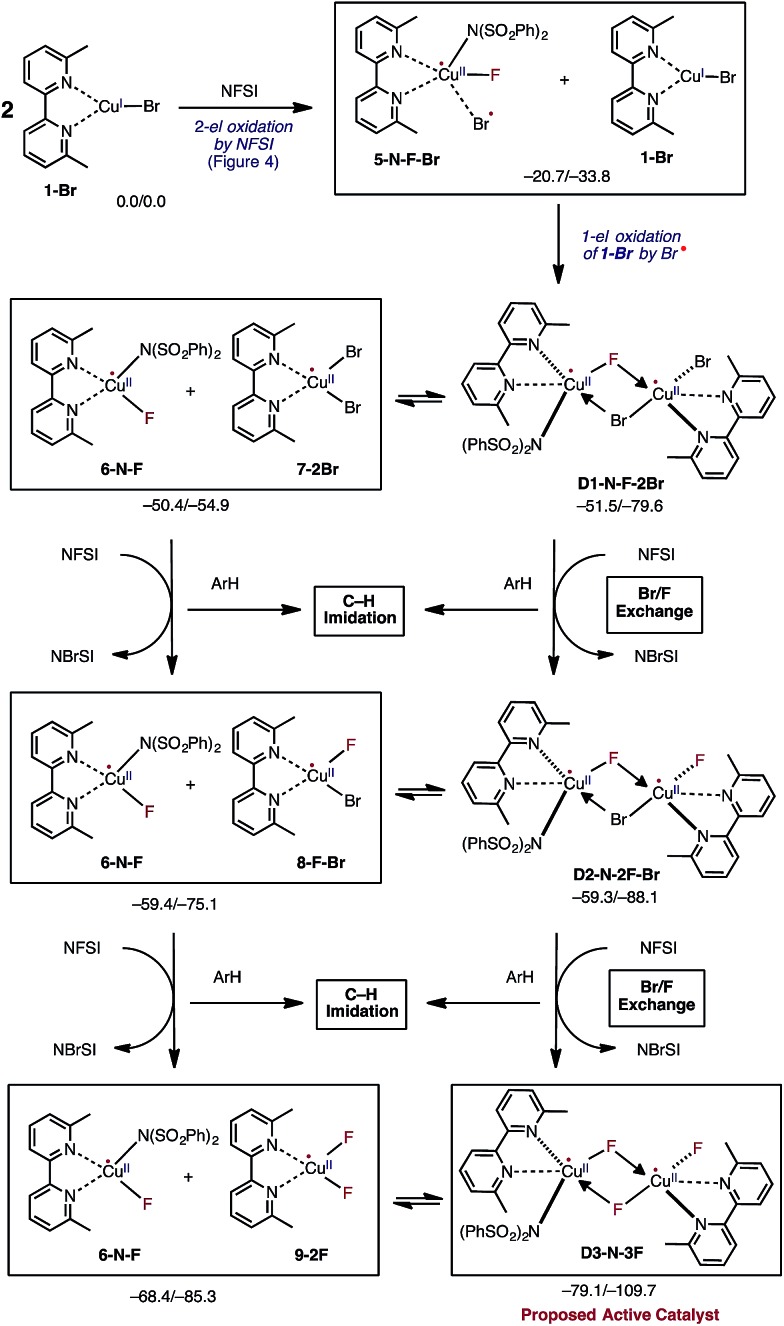
Mechanistic steps of the reaction of two molecules of LCu^I^Br with three molecules of oxidant NFSI that leads to generation of the catalytically active dinuclear Cu^II^–Cu^II^ (**D3-N-3F**) catalyst. This process is referred to Br/F exchange in the text. Relative energies are given as Δ*G*/Δ*H* in kcal mol^–1^.

In the next step, the resulting **5-N-F-Br** complex reacts with another molecule of LCu^I^Br (**1-Br**) and forms a dinuclear Cu^II^–Cu^II^ complex **D1-N-F-2Br** with one terminal Br ligand and bridging F and Br ligands (Fig. 5). The formation of **D1-N-F-2Br** involves (a) one-electron oxidation of the second **1-Br** complex by the bromine radical of **5-N-F-Br**, and (b) complexation (*i.e.* two mono-nuclear complexes → one dinuclear complex).^66^ Since the reaction **5-N-F-Br** + **1-Br** → **D1-N-F-2Br** is highly exergonic (Δ*G* = –30.8 kcal mol^–1^), it is reasonable to conclude that in the reaction mixture **1-Br** will be fully converted to **D1-N-F-2Br** by NFSI.

Thus, the overall reaction 2 (**1-Br**) + NFSI → **D1-N-F-2Br** consists of concurrent one-electron oxidation of two distinct mononuclear Cu^I^ complexes by one NFSI molecule (Fig. 4 and 5). In the closely related literature analogs of bimetallic oxidation by one molecule of oxidant,^67–70^ the oxidant simultaneously oxidizes two metal centers by one-electron each. However, in the reported reaction of CuBr with NFSI, the oxidant is added to the same metal center and the auxiliary ligand (*i.e.*, Br) is responsible for oxidation of the second metal center. Thus, the distinction between what we propose and the examples in the literature for bimetallic oxidation lies in the fate of the oxidant. Furthermore, it is important to emphasize that the newly reported mechanism of the reaction of LCu^I^Br with one molecule of NFSI does not produce the reactive imidyl radical and, consequently, cannot lead directly to C–N bond formation. This novel finding is profoundly different from the two-electron oxidative addition mechanism of this reaction proposed by Zhang and coworkers.^54^

Close examination of the dinuclear Cu^II^–Cu^II^ complex **D1-N-F-2Br** shows that it is in equilibrium with two mono-nuclear Cu^II^ complexes: the disproportionation reaction **D1-N-F-2Br** → LCu^II^F[NSI] (**6-N-F**) + LCu^II^Br_2_ (**7-2Br**) is only slightly endergonic (Δ*G* = 1.1 kcal mol^–1^, see Fig. 5). Therefore, one may expect that both dinuclear **D1-N-F-2Br** and mono-nuclear **7-2Br** complexes will be present in the reaction mixture and can react with another molecule of NFSI (Fig. 5).^71^ Surprisingly, regardless of the nuclearity of the reactant complexes, *i.e.* starting from either dinuclear **D1-N-F-2Br** or mono-nuclear **7-2Br** complexes, the reaction with a second NFSI molecule leads to exchange of one Br ligand for a F ligand and concomitant formation of *N*-bromobenzenesulfonimide (NBrSI).

The mechanism for this process (called here Br/F exchange) is very complex: it is initiated by oxidation of the Cu^II^ species by NFSI to form Cu^III^ and an imidyl radical. As expected, the barrier for oxidation of the Cu^II^ complexes (Δ*G*‡FT = 25.9 and 23.6 kcal mol^–1^ for the dinuclear **D1-N-F-2Br** and mono-nuclear **7-2Br** Cu^II^ complexes, respectively) is much higher than the barrier for oxidation of the Cu^I^ complex **1-Br** (Δ*G*‡FT = 5.4 kcal mol^–1^). Then, extraction of a bromine radical by the generated imidyl radical produces NBrSI and Cu^II^, as shown in Fig. 5 (see the ESI† for more detailed discussion and free energy surface for this process).

Even though Br/F exchange from **D1-N-F-2Br** and **7-2Br** is exergonic by 7.8 and 8.9 kcal mol^–1^, respectively, it is possible that a small amount of the imidyl radical will be able to escape from the Cu solvation shell to react with the substrate. However, this is not expected to lead to a high level of C–N product formation due to fast imidyl–bromine radical combination leading to NBrSI formation.

The resulting dinuclear complex **D2-N-2F-Br** (with only one Br ligand) and mono-nuclear complexes LCu^II^F(NSI) (**6-N-F**) + LCu^II^BrF (**8-F-Br**) are in equilibrium (Δ*G* = 0.1 kcal mol^–1^), and can react with a third NFSI molecule. The reaction of **D2-N-2F-Br** and NFSI leads to formation of the dinuclear complex [LCu^II^F(NSI)···LCu^II^F_2_] **D3-N-3F**, and a second molecule of NBrSI. The resulting dinuclear complex **D3-N-3F** has three fluoride ligands (*i.e.*, no Br ligands) and is the energetically most stable intermediate in the series of Br/F exchange processes shown in Fig. 5. Interestingly, this dinuclear complex is stable relative to disproportionation to the mono-nuclear complexes LCu^II^F(NSI) (**6-N-F**) + LCu^II^F_2_ (**9-2F**) by Δ*G* = –10.7 kcal mol^–1^.

**Fig. 6:**
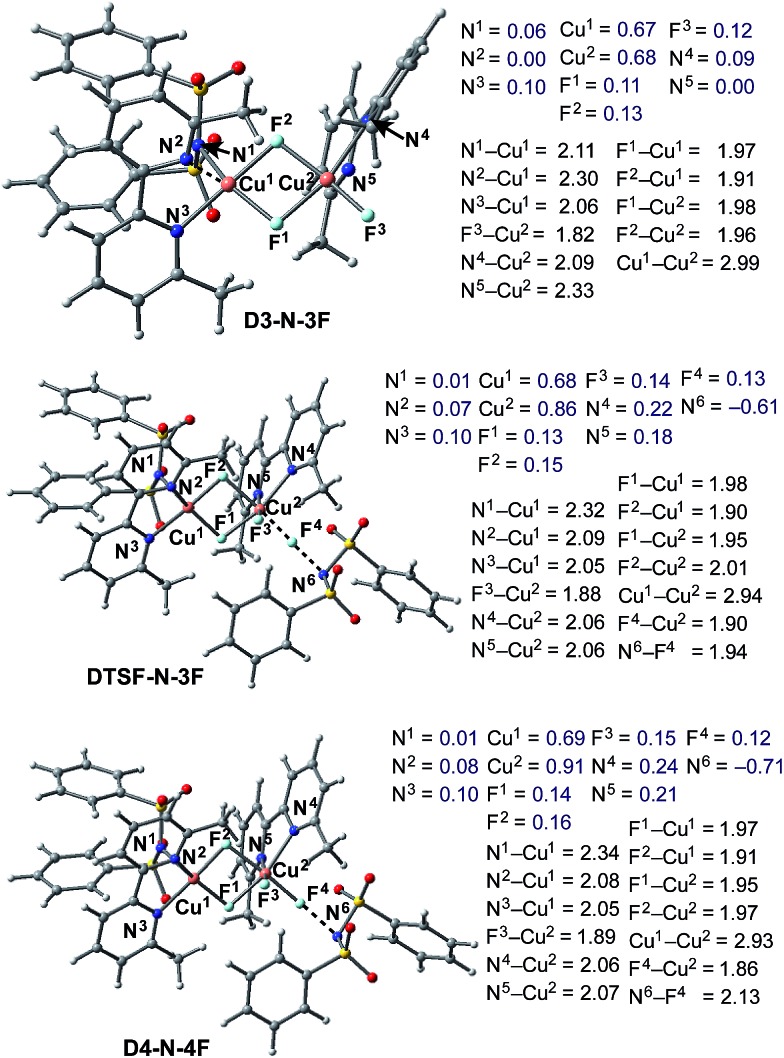
Structural analysis of the active catalyst (**D3-N-3F**), antiferromagnetic F-atom transfer transition state (**DTSF-N-3F**) and imidyl radical product complex (**D4-N-4F**) involved in the oxidation of **D3-N-3F** by NFSI. Bond distances (in Å) are shown in black and Mulliken spin density values (in |*e*|) are shown in blue.

Briefly, the dinuclear complex [LCu^II^F(NSI)···LCu^II^F_2_] **D3-N-3F** has the characteristic diamond core with bridging fluoride ligands and two square planar Cu^II^ centers sitting in the same plane^72^ (Fig. 6). The diamond core of **D3-N-3F** closely resembles that of other dinuclear Cu_2_F_2_ complexes that have previously been extensively characterized,^73–77^ but their role in catalysis is under-appreciated. In **D3-N-3F**, both bipyridine ligands are asymmetrically bound: each Cu^II^ center has one pyridine loosely coordinated at its axial position (the calculated N^2^–Cu^1^ and N^5^–Cu^2^ bond distances are 2.30 and 2.33 Å, respectively). As shown across Fig. 3–5, the overall Br/F exchange reaction 2 **1-Br** + 3 NFSI → **D3-N-3F** + 2 NBrSI, is highly exergonic (Δ*G* = –79.1 kcal mol^–1^) and requires a maximum free energy barrier of 24.7 kcal mol^–1^ (see the ESI†), which is reasonable for the experimental reaction conditions. Thus, the dinuclear Cu^II^–Cu^II^ complex **D3-N-3F** is the sole and thermodynamically most stable Cu species from the reaction of two molecules of Cu^I^ complex **1-Br** with three molecules of NFSI oxidant involving a series of redox steps and Br/F exchange.

### Generation of the reactive imidyl radical

**Fig. 7:**
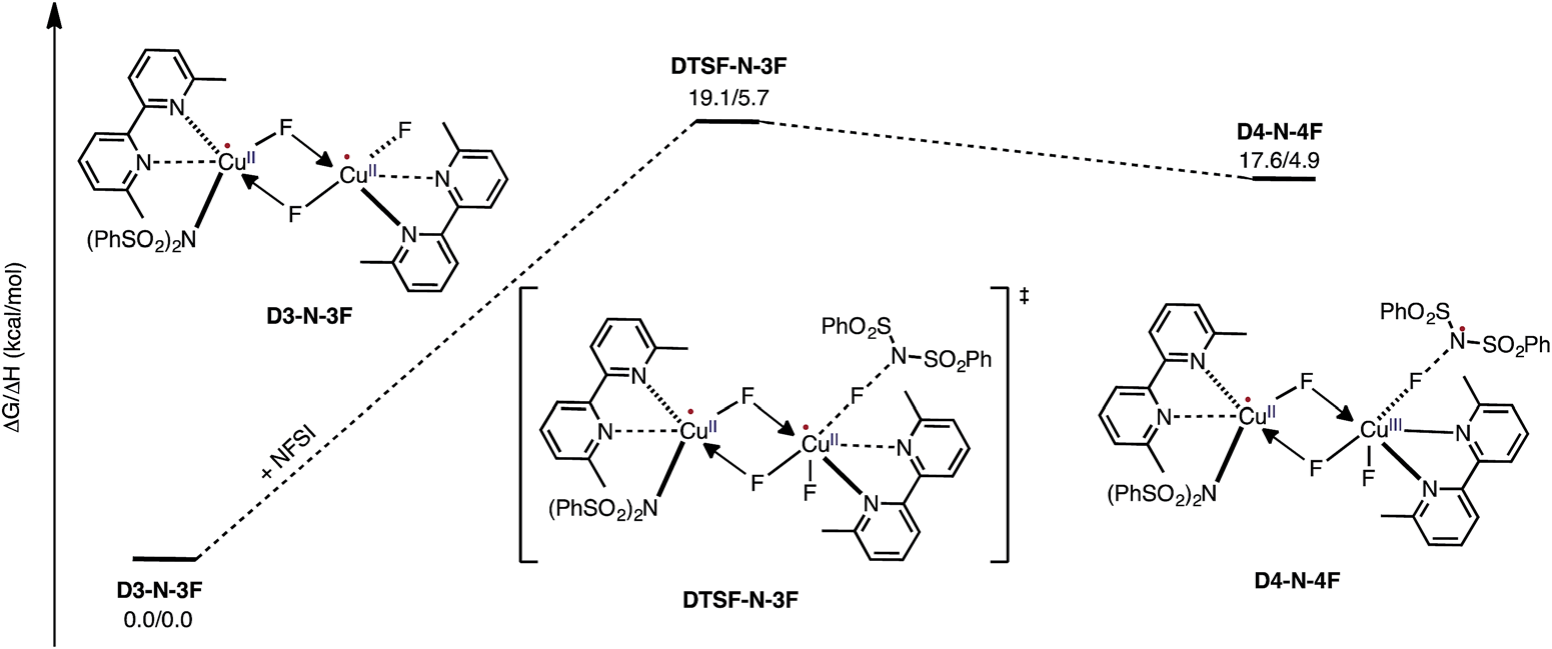
Free energy profile for oxidation of the catalytically active dinuclear Cu^II^–Cu^II^ complex **D3-N-3F** by NFSI and the formation of the reactive imidyl radical complex (**D4-N-4F**) on the triplet surface.

Armed with the aforementioned findings, we examined the reaction of **D3-N-3F** with a fourth molecule of NFSI. The barrier for this reaction, at the antiferromagnetic F-atom transfer transition state **DTSF-N-3F**, is found to be Δ*G*‡FT = 19.1 kcal mol^–1^ (Fig. 7).

As shown in Fig. 6, in the transition state, **DTSF-N-3F**, F^4^ is transferred from NFSI to Cu^2^ *trans* to the bridging fluoride ligand F^2^. This process triggers ligand rearrangements on the dinuclear Cu core. The imidyl group rotates to the axial position of Cu^1^ resulting in the N^1^–Cu^1^ bond elongates to 2.32 Å. This is likely due to rearrangement of F^3^ to the axial position of Cu^2^ in order to accommodate the incoming F^4^. At the same time, the bipyridine ligands on each Cu center go from the aforementioned asymmetric coordination mode to a symmetric coordination mode (N^2^–Cu^1^ 2.09 Å and N^5^–Cu^2^ 2.06 Å) to provide additional stabilization to the oxidized structure.

This oxidation reaction is endergonic by Δ*G* = 17.6 kcal mol^–1^ (Fig. 7), suggesting that the imidyl radical will be present in low concentrations so that the likelihood of homo-coupling or other side reactions involving imidyl radicals is reduced. In the product complex **D4-N-4F**, the Cu^III^ center has an octahedral geometry. Oxidation of this Cu center is evident by an increase in its unpaired Mulliken spin from **D3-N-3F** (0.68|*e*|) to **DTSF-N-3F** (0.86|*e*|) to **D4-N-4F** (0.91|*e*|), while significant delocalization of unpaired spin is found on the ligands. In addition, the Cu–Cu distance decreases (2.99 → 2.93 Å) indicating weak coupling between the Cu centers: possibly indicating the formation of a weak two-center/one-electron (or less) Cu–Cu bond (see the ESI† for more analysis). Therefore, it is a reasonable to conclude that the dinuclear structural motif, additional fluoride ligands and bipyridine ligands together play a significant role in facilitating the oxidation process. This statement is also supported by the calculation of the barrier for oxidation of the mononuclear Cu^II^ complex **9-2F** by NFSI: we found that the barrier of this reaction is Δ*G*‡FT = 28.4 kcal mol^–1^, which is 9.3 kcal mol^–1^ higher than oxidation of the dinuclear complex **D3-N-3F** by NFSI.

Therefore, in contrast to the active catalyst generation steps, NFSI reacts with the catalytically active dinuclear Cu^II^–Cu^II^ complex, **D3-N-3F**, as a one-electron oxidant to generate a dinuclear Cu^II^–Cu^III^ intermediate and a reactive imidyl radical through a reasonable energy barrier (Δ*G*‡FT = 19.1 kcal mol^–1^). Based on the aforementioned mechanistic findings, we predict that the identity of the LCu^I^X pre-catalyst, where X = Cl, Br, and I, is not directly connected to the imidation reactivity, so these pre-catalysts should show similar reactivity upon loss of their X groups and *in situ* formation of the same active dinuclear Cu^II^–Cu^II^ catalyst (**D3-N-3F**) when reacting with NFSI. [For more details of the reaction of LCuX (where X = F, Cl and I) with NFSI, see the ESI†].

### Experimental validation

Thus, the computations predict that, at the initial stage of the reaction of LCu^I^Br with NFSI, all of the LCu^I^Br complexes (10 mol% under the standard conditions) converge irreversibly to the active dinuclear Cu^II^–Cu^II^ catalyst (**D3-N-3F**) and NBrSI. In order to validate these computational findings, we launched extensive experimental studies.

**Fig. 8:**
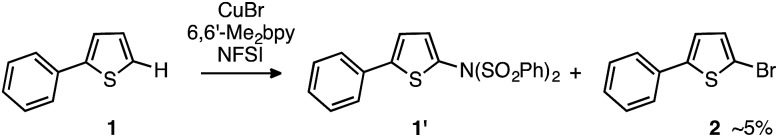
Detection of brominated substrate, **2**, during the reaction with 2-phenylthiophene, **1**.

At first, considering NBrSI as an electrophilic brominating reagent similar to *N*-bromosuccinimide (NBS), we suspected that its generation *in situ* might lead to unwanted side reactions. Indeed, we found that the reaction of 2-phenylthiophene, **1**, with NFSI in the presence of LCu^I^Br (10 mol%) produces 5% of 2-bromo-5-phenylthiophene, **2**, as a side product (Fig. 8).

**Fig. 9:**
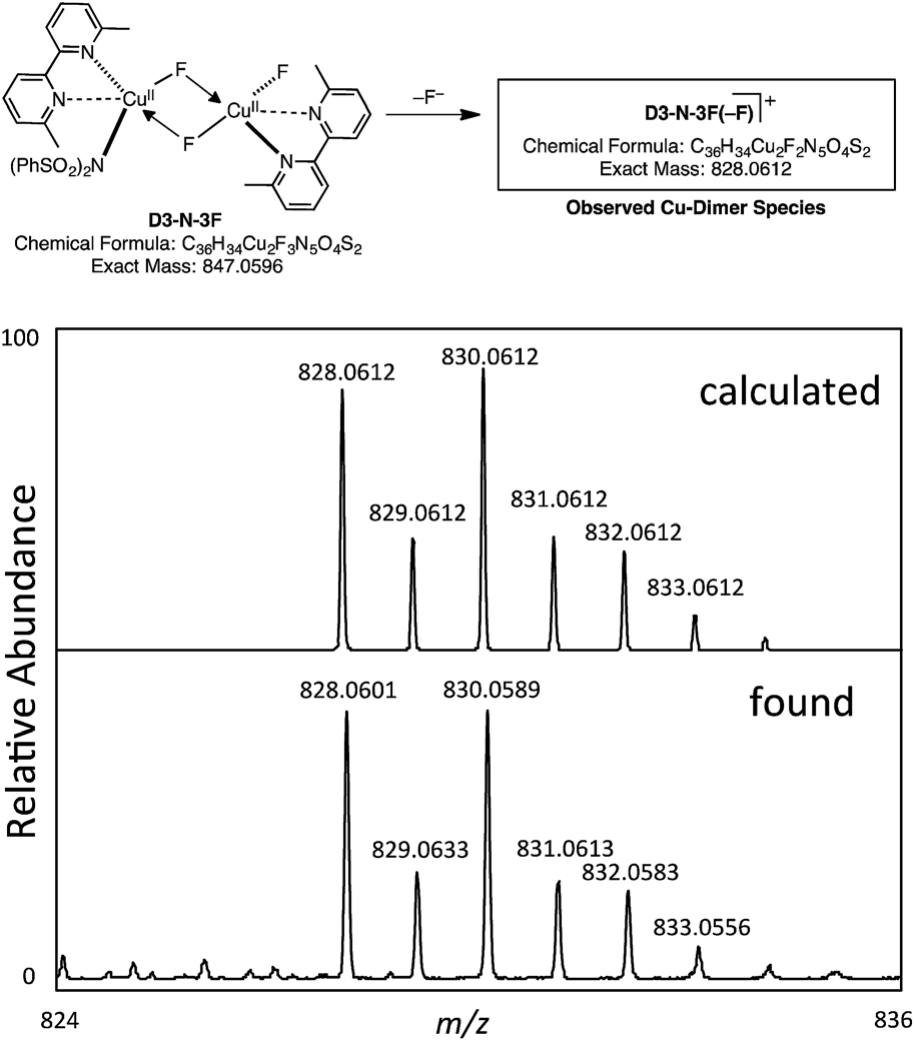
Observation of a copper-dimer species **D3-N-3F(–F)** by HRMS.

However, our extensive efforts to detect NBrSI in the reaction mixture by HRMS were not successful. Alternatively, it is also possible that an *in situ* generated CuBr species or Br_2_ can act as a brominating reagent. This result does support the computational prediction that the initial CuBr catalyst is converted to a different species through loss of its bromide ligand. As described above, the computations predict that the result of the Br/F exchange process is **D3-N-3F**. Gratifyingly, we were able to detect by HRMS a copper-dimer species **D3-N-3F(–F)**, which would be generated from **D3-N-3F** in the same mixture (Fig. 9) (it is also possible that loss of Br from **D2-N-2F-Br**, but computations suggest that **D3-N-3F** is more likely based on thermodynamics). Thus, we experimentally confirmed the generation of **D3-N-3F** in the reaction system, as predicted by the computation.

**Fig. 10:**
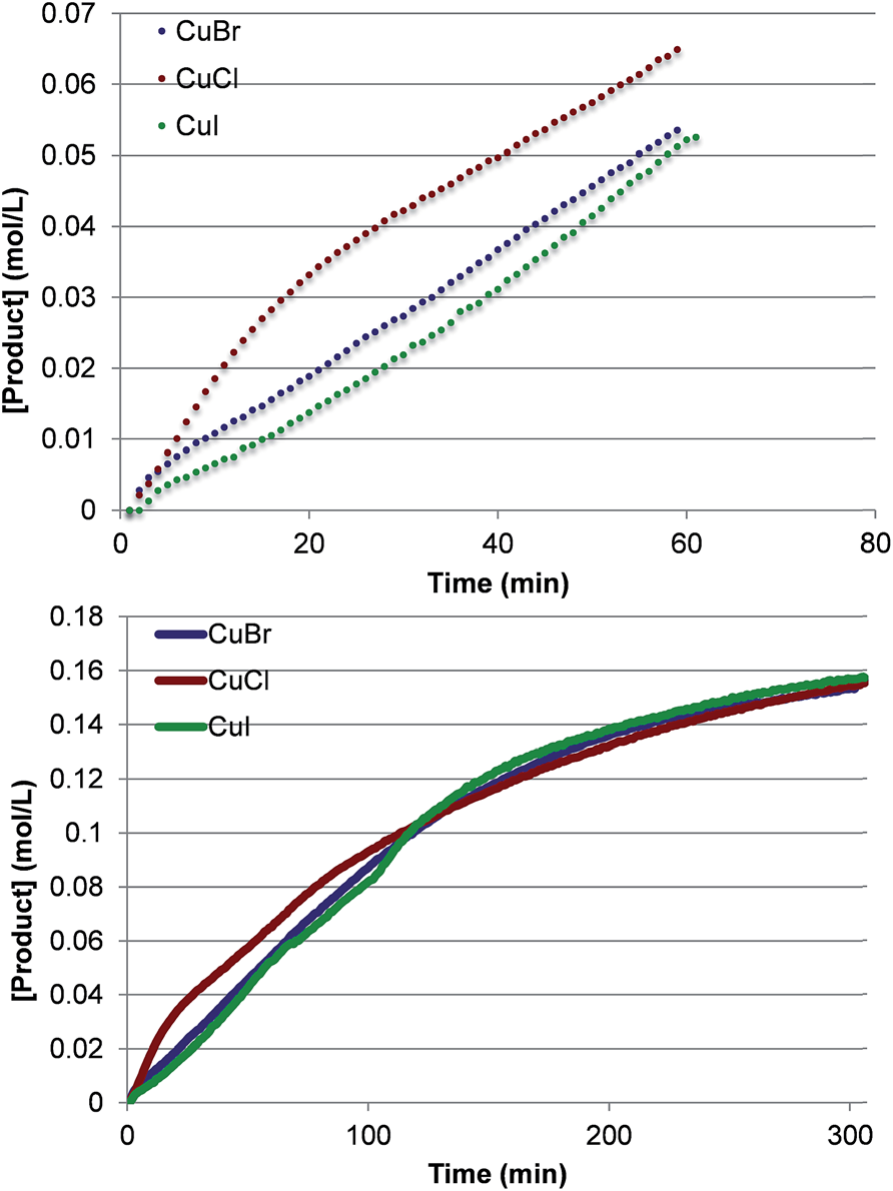
Kinetic profiles at short times (top) and the full time course (bottom) of product formation for pre-catalysts LCuX, where X = Cl, Br, and I.

To investigate further the nature of the active catalyst and to validate the conclusions of the computations, we also performed the reaction with various LCuX complexes, where X = Cl, Br and I. Interestingly, the reaction with **1** produces similar yield of imidated product after 5 h regardless of the identity of X: 77%, 77% and 78% with CuCl, CuBr, and CuI, respectively. In addition, the kinetic profiles of the reaction with these pre-catalysts show that over the full time course each reaction converges to the same rate (Fig. 10). This suggests that each pre-catalyst of the reaction is converted to the same species, which acts as the predominant catalyst in the reaction. Together these experimental findings and the aforementioned computational data allow us to conclude reasonably that the C–H imidation activity of LCuX complexes with NFSI is catalyzed by a dinuclear Cu^II^–Cu^II^ complex, **D3-N-3F**, that is generated *in situ* from LCuX precursors. We expect that these findings will provide insight for other reactions catalyzed by Cu^I^-salts, and in particular, will aid in the design of more efficient and inexpensive pre-catalysts for the reaction that, for example, do not require excess NFSI or sacrifice an amount of the substrate.

### Radical addition and generation of C–N imidation product

**Fig. 11:**
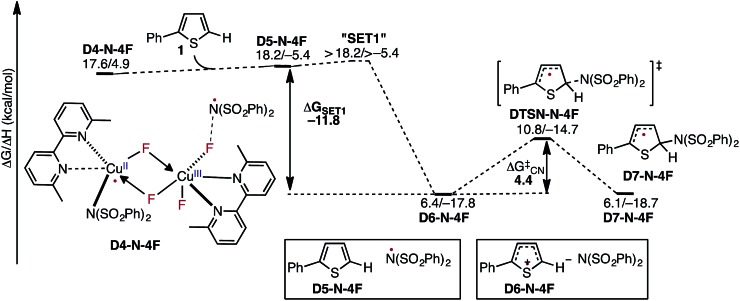
Free energy surface for the stepwise mechanism for C–N bond formation starting with electron transfer from **1** to the imidyl radical (**SET1**) followed by C–N bond formation by the resulting ion-pair to produce an aryl radical intermediate. All energies are computed relative to **D3-N-3F** + NFSI and are calculated for the energetically lowest antiferromagnetically coupled triplet states except **D7-N-4F**, for which this electronic state is not stable. Therefore, for **D7-N-4F** we report the quintet electronic state energy, which is very close to that for the triplet state for all the other structures (see the ESI†). The dinuclear Cu complex is included in all calculations but has been removed from the Figure for clarity.

**Fig. 12:**
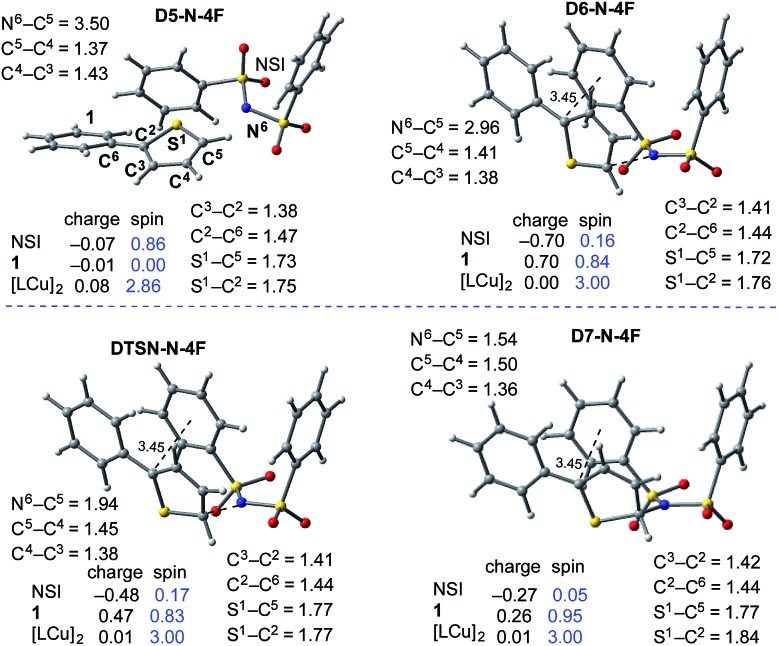
Geometric and electronic structure analysis of the intermediates of C–N bond formation between the imidyl radical and **1**. Bond distances (in Å) are shown in black and Mulliken spin density values (in |*e*|) are shown in blue. The dinuclear Cu complex has been removed from the Figure for clarity. The structures on the quintet and triplet electronic states are geometrically, electronically, and energetically similar (see the ESI†). For the antiferromagnetically-coupled triplet electronic states the spin values of the NSI and **1** fragments have the opposite sign.

In the next step, association of the imidyl radical of **D4-N-4F** with the 2-phenylthiophene (**1**) substrate forms a weakly coordinated complex **D5-N-4F**. This process is almost thermoneutral (Δ*G* = 0.6 kcal mol^–1^) (Fig. 11). However, as the imidyl radical and **1** approach each other (N^6^–C^1^ = 3.50 Å → 2.96 Å, Fig. 12), single electron transfer (**SET1**) occurs from the π-system of **1** to the imidyl radical. This process is exergonic (Δ*G*_**SET1**_ = –11.8 kcal mol^–1^) and leads to the formation of intermediate **D6-N-4F**, which can be characterized as an ion-pair between an imidyl anion and aryl radical-cation. Electron transfer in this complex is evidenced by the build-up of unpaired spin (0.84|*e*|) and positive charge (0.70|*e*|) on the ring of **1** (Fig. 12). Unfortunately, we are not able to accurately compute the exact value of the free energy barrier required for **SET1** with the methods used in this study. Therefore, we estimate it to be greater than the energy of the pre-reaction complex **D5-N-4F**, *i.e.* >18.2 kcal mol^–1^ (Fig. 11).

From the product of the single electron transfer step (**D6-N-4F**), C–N bond formation occurs through an energy barrier of Δ*G*‡CN = 4.4 kcal mol^–1^ (at the transition state **DTSN-N-4F**) and leads to the aryl radical intermediate **D7-N-4F** ^78^ (Fig. 11). Thus, C–N bond formation (*i.e.*, the reaction of **D4-N-4F** with **1**) occurs through a stepwise mechanism involving (i) single electron transfer (**SET1**) from the π-system of **1** to the imidyl radical of **D4-N-4F**, and (ii) C–N bond formation at the transition state **DTSN-N-4F**.

**Fig. 13:**
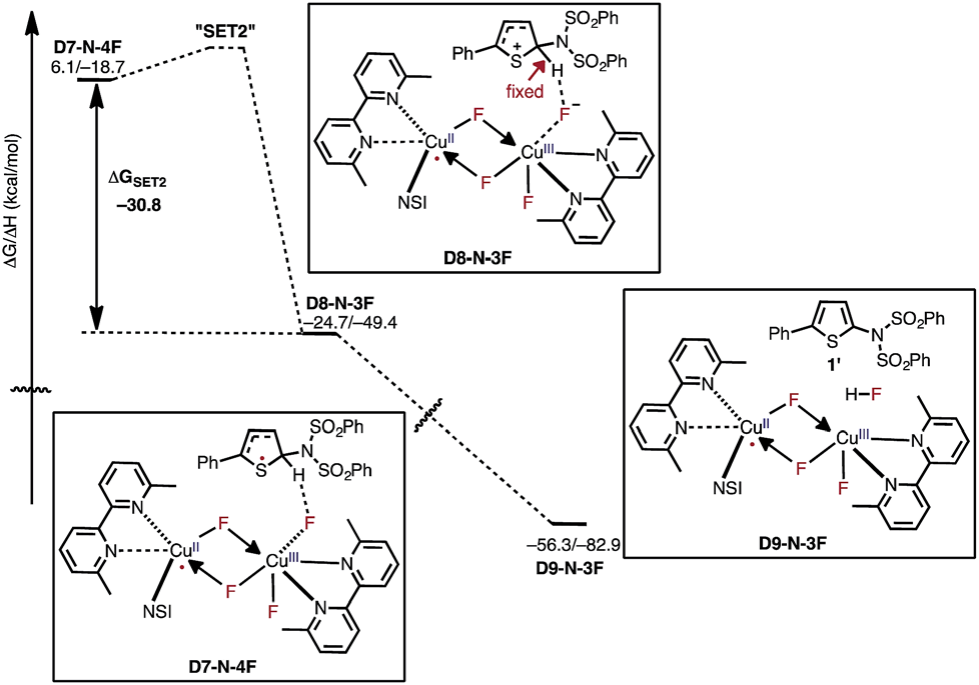
Free energy surface for active catalyst regeneration and product formation, which proceeds through an electron transfer to the catalyst and ejection of fluoride anion. The fluoride anion then deprotonates and rearomatizes the aryl cation intermediate to generate the imidated product and HF. The presented energies are computed relative to the **D3-N-3F** + NFSI dissociation limit.

After the aryl radical is formed, the next step of the reaction is another single electron transfer (**SET2**) from the aryl radical to the dinuclear Cu^II^–Cu^III^ complex (Fig. 13). This step should produce an aryl cation and dinuclear Cu^II^–Cu^II^ complex with a coordinated fluoride anion (**D8-N-3F**). However, all our attempts to locate this structure resulted in the deprotonation product **D9-N-3F**. To estimate the thermodynamic stability of **D8-N-3F**, we optimized the structure by fixing the C–H bond distance to 1.09 Å, and found that **SET2** is thermodynamically favored by Δ*G* = –30.2 kcal mol^–1^. The fact that **D8-N-3F** could not be located without geometry constraint indicates that subsequent deprotonation and rearomatization of the substrate by fluoride anion is barrierless (see the ESI† for energy scans). The formation of the product, HF, and the active catalyst (**D3-N-3F**) in complex **D9-N-3F** is highly exergonic from **D8-N-3F** (Δ*G* = –31.6 kcal mol^–1^). Indeed, the overall reaction, **D3-N-3F** + NFSI + **1** → **D3-N-3F** + **1′** + HF is calculated to be Δ*G* = –62.8 kcal mol^–1^ exergonic.

Careful analysis of the energy span^79^ (δ*E*) for the entire free energy surface of the reaction of **D3-N-3F** with NFSI and **1** substrate indicates that its turnover-determining intermediate (TDI) is **D3-N-3F** + NFSI + **1**. However, based only on the presented energy calculations, it is not straightforward to determine the turnover-limiting TS (TDTS) of the reaction because we were not able to compute accurately the energy barrier associated with the **SET1** step (*i.e.*, electron transfer from the substrate to the imidyl radical), the actual mechanism of which depends on many factors including (but not limited to) the nature of solvent and driving force for electron transfer. Furthermore, its estimated value of >18.2 kcal mol^–1^ is very close to the 19.1 kcal mol^–1^ energy barrier calculated for oxidation of **D3-N-3F** by NFSI. Since these two barriers are shown to be the largest energy barriers on the full potential energy surface of the reaction, we expect either oxidation of **D3-N-3F** by NFSI or **SET1** to be the turnover-limiting step of overall reaction.

**Fig. 14:**
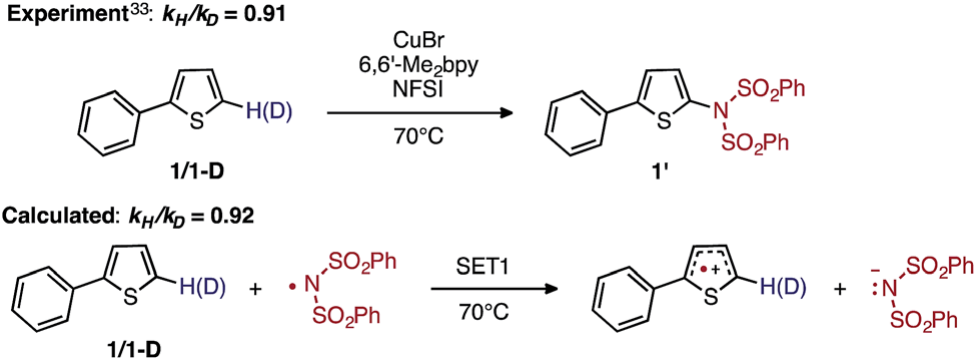
Schematic presentation of the experimental^33^ and computational isotope effect studies for electron transfer from **1** to the imidyl radical.

To discriminate between these possible turnover-limiting steps, we examined the H/D substrate kinetic isotope effect (KIE). The oxidation step (*i.e.*, **D3-N-3F** → **DTSF-N-3F** → **D4-N-4F**) does not involve the substrate, so if it is the turnover-limiting step, there should not be any substrate KIE. In contrast, the calculated isotope effect for the **SET1** step (*i.e.*, **D5-N-4F** → **D6-N-4F**), which is found to be *k*_H_/*k*_D_ = 0.92 at 70 °C, (calculated from the 5-hydrogen and 5-deuterium isotopologues of **1**, see the ESI† for more details) is in excellent agreement with the previously determined experimental^33^ KIE of *k*_H_/*k*_D_ = 0.91 at 70 °C as shown in Fig. 14.

Based on the calculated and measured KIEs, we conclude that the **SET1** step is the turnover-limiting step of the reaction. The calculated KIE can be attributed to differences in the zero point energies of harmonic frequencies arising from delocalization of the adjacent sulfur lone pair into the anti-bonding orbital (n–σ*) of the C–H and C–D bonds.^80–83^ Although measured inverse KIEs often are attributed to the change in C(sp)^2^ → C(sp)^3^ hybridization in the TS,^34–37^ previous studies have also shown that electron transfer reactions can produce inverse isotope effects.^84,85^

**Fig. 15:**
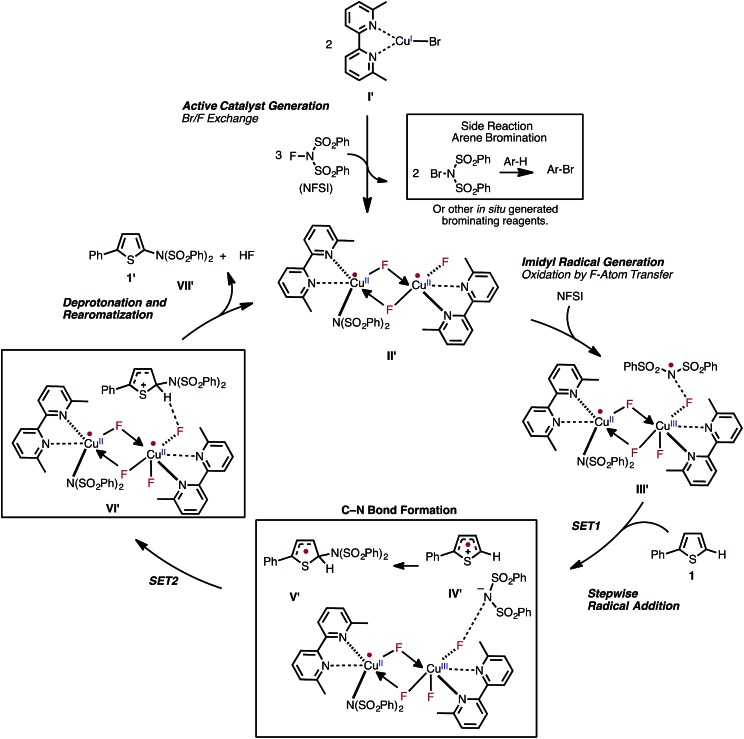
The newly proposed mechanism for Cu-catalyzed aromatic C–H imidation by NFSI based on the collaborative computational and experimental results described in this study. The novel reaction mechanism has two major parts: (1) generation of the dinuclear Cu^II^–Cu^II^ active catalyst; and (2) subsequent catalytic cycle for aromatic C–H imidation with NFSI.

Based on the aforementioned computational and experimental findings, we propose a novel dinuclear mechanism for the LCu^I^Br-catalyzed aromatic C–H imidation with NFSI that includes the following steps (Fig. 15): (1) conversion of the LCu^I^Br pre-catalyst (**I**) through a series of Br/F exchange reactions with NFSI to the active dinuclear Cu^II^–Cu^II^ catalyst (**II′**); (2) oxidation of the active catalyst by NFSI *via* one-electron F-atom transfer pathway that generates a reactive imidyl radical (**III′**); (3) stepwise C–N bond formation comprising of turnover-limiting single-electron-transfer (**SET1**) from the substrate to the imidyl radical produces an ion-pair (**IV′**), and subsequently fast C–N bond coupling to form an aryl radical (**V′**); (4) reduction of the dinuclear Cu^II^–Cu^III^ intermediate by the aryl radical that produces an aryl cation, fluoride anion; and (5) deprotonation and rearomatization of the arene ring by the fluoride anion to form the imidated product and HF (**VII′**) and regeneration of the active catalyst (**II′**).

The above-presented mechanism for the LCu^I^Br-catalyzed C–H imidation with NFSI provides an opportunity to examine the effect of the substrate on the reaction, to improve the efficiency of the catalysis, and to widen substrate scope.

### Substrate effect

A major strength of this reaction is its applicability to a wide range of arene substrates. However, to further expand its activity to entirely new substrates requires better understanding of the factors impacting the reactivity of a given substrate. Based on the computed mechanism above, we hypothesized that the reactivity of a given substrate will depend on its ability to induce electron transfer. It is natural to expect that electron-rich substrates will most easily be oxidized by the imidyl radical. To further probe this hypothesis, we computationally investigate the mechanism of the reaction for benzene, which experimentally produces lower yields than 2-phenylthiophene.^33^

**Fig. 16:**
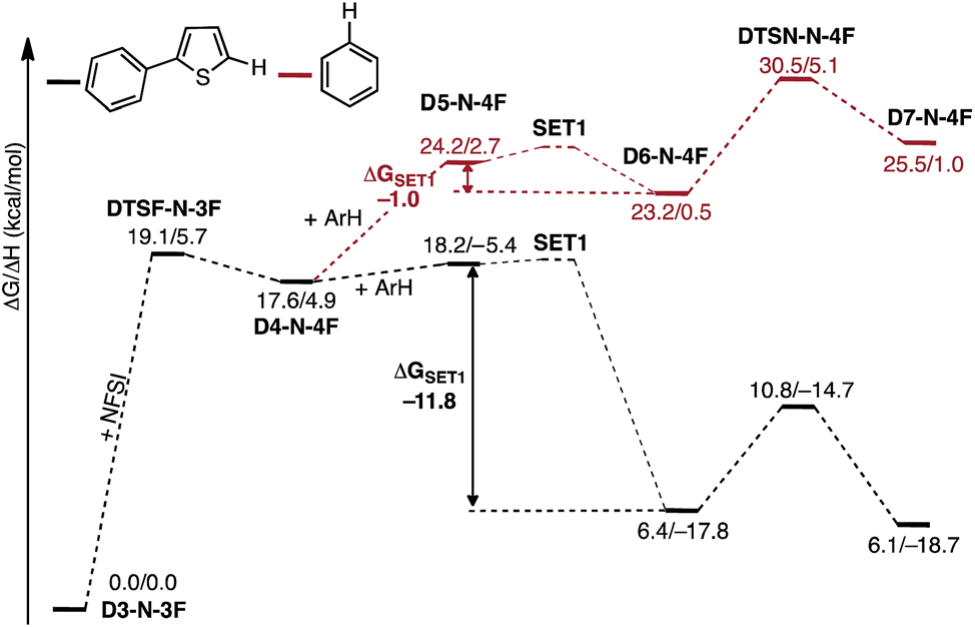
Free energy surfaces for stepwise C–N bond formation with 2-phenylthiophene (black) and benzene (red).

As shown in Fig. 16, the **SET1** (*i.e.*, **D5-N-4F** → **D6-N-4F**) step for benzene (Δ*G*_**SET1**_ = –1.0 kcal mol^–1^) is energetically much less favorable than for 2-phenylthiophene (Δ*G*_**SET1**_ = –11.8 kcal mol^–1^). In addition, oxidation of benzene is dramatically more endergonic than 2-phenylthiophene, calculated relative to the **D3-N-3F** + substrate. As a result, the **SET1** step and the subsequent C–N bond formation transition state are pushed much higher in energy with benzene. This outcome is consistent with the experimental observation that excess benzene is required to generate low yields of product.^33^ This finding also explains why electron-rich arenes react faster than electron-deficient arenes in competition experiments.^33^

### Factors impacting the regioselectivity of the reaction

The proposed new mechanism of the Cu-catalyzed aromatic C–H imidation by NFSI is also consistent with the experimentally observed high-levels of regioselectivity of the reaction. Since the turnover-limiting step (**D3-N-3F** → **SET1**) of the reaction is indiscriminate toward the regioselectivity, it is reasonable to expect that the relative barriers for C–N bond formation at each position will dictate the regioselectivity (*i.e.*, C–N bond formation is the regioselectivity-determining step). Therefore, we hypothesized that the positive charge on the arene carbons bearing hydrogen atoms in the oxidized substrate (*i.e.*, radical cation) will be predictive for the regiochemical outcome of the reaction. To validate this hypothesis, we calculated the Hirshfeld charges^86^ of the radical cation for a number of substrates used in the experiment.^33^ This approach is similar to the “charge-transfer-directed” concept of Ritter and coworkers.^31,32^

**Fig. 17:**
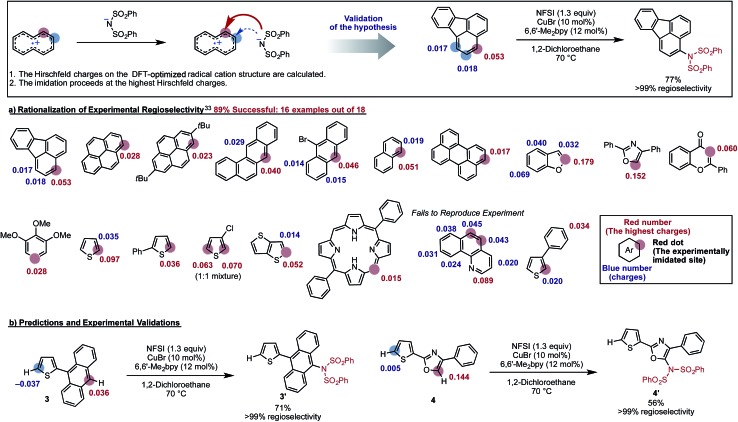
Validation of a model to predict the site of C–H imidation based on the calculated Hirshfeld charges (shown in red and blue) on the DFT-optimized radical cation structure of each substrate. The experimentally imidated site is indicated by a red sphere and the site with the highest positive charge is indicated with red numbers. Other possible sites for imidation are indicated with blue spheres. Using this model, regioselectivity predictions were made for new substrates, **3** and **4**. The predictions for these substrates were then validated experimentally producing the predicted product with >99% selectivity.

We find excellent agreement between the proposed charge model and the experimentally observed regioselectivity in most cases (Fig. 17). To show the utility of this simple predictive tool, we predicted that imidation of **3** and **4** would occur on the anthracene and oxazole rings of these substrates, respectively. These predictions were then confirmed by experiments showing that the predicted imidated products were formed with >99% regioselectivity (Fig. 17).

Close examination of the substrates where the regioselectivity predictions fail (see Fig. 17) also gives useful insight into the limitations of our predictive model, such as: (a) the assumption that the deprotonation and rearomatization step of the reaction is faster than **SET1**;^87^ and (b) not including the role of external directing effects such as hydrogen bonds, π-stacking, steric hindrance or other non-covalent interactions. However, we expect that the user can easily anticipate these limitations of the model and generate an acceptably accurate prediction of the site of imidation in a given molecule quickly and reliably (see the ESI† for the detailed procedure of this simple predictive tool).

## Conclusions

Above, we utilized a joint computational and experimental collaboration to obtain an in-depth understanding of the mechanism and governing factors of Cu-catalyzed aromatic C–H imidation with NFSI and propose a novel dinuclear mechanism (see Fig. 15) for this reaction. Briefly,

(1) We found that prior to initiation of the catalytic cycle NFSI acts like a two-electron oxidant by reacting with two molecules of LCu^I^Br and producing the dinuclear Cu^II^–Cu^II^ complex [LCu^II^F(NSI)···LCu^II^Br_2_], **D3-N-F-2Br**. The predicted bimetallic oxidation reaction with one molecule of oxidant is unprecedented because here NFSI directly interacts only with one equivalent of LCu^I^Br resulting in simultaneous one-electron oxidation of both the Cu^I^ center and Br-ligand, which then oxidizes the second molecule of LCu^I^Br.

(2) In contrast, in the next stage of the reaction NFSI acts as overall redox neutral molecule: two molecules of NFSI react with the dinuclear Cu^II^–Cu^II^ complex **D3-N-F-2Br** *via* the Br/F exchange pathway and produce two molecules of NBrSI (or other Br-containing molecules) and the catalytically active dinuclear Cu^II^–Cu^II^ complex [LCu^II^F(NSI)···LCu^II^F_2_], **D3-N-3F**. The resulting dinuclear Cu^II^–Cu^II^ complex is predicted to be the thermodynamically most stable Cu species of the reaction. Subsequent HRMS experiments detected a copper-dimer species **D3-N-3F(–F)** and confirmed the generation of the dinuclear Cu^II^–Cu^II^ complex **D3-N-3F** in the reaction system, as predicted by computation.

(3) Since the dinuclear Cu^II^–Cu^II^ complex [LCu^II^F(NSI)···LCu^II^F_2_], **D3-N-3F**, is shown to be the catalytically active species, we predicted that the identity of the LCu^I^X pre-catalyst, where X = Cl, Br, and I, is not directly connected to its imidation activity. This prediction of computation also was validated by experiments by showing that (a) the reaction with 2-phenylthiophene produces a similar yield regardless of the identity of X, and (b) over the full time course the kinetic profile of the reaction with LCu^I^X converges to the same rate.

(4) The catalytic cycle starts from the reaction of the dinuclear Cu^II^–Cu^II^ complex **D3-N-3F** with NFSI, as a one-electron oxidant, and forms the reactive imidyl radical and the Cu^II^–Cu^III^ dinuclear intermediate. This reaction subsequently proceeds through the following elementary steps: (a) stepwise C–N bond formation between the substrate and imidyl radical proceeding *via* turnover-limiting single-electron-transfer (**SET1**) from the substrate to the imidyl radical followed by fast imidyl anion and aryl radical-cation coupling to produce an aryl radical intermediate; (b) reduction of the Cu^II^–Cu^III^ dinuclear intermediate by the resulting aryl radical to regenerate the active catalyst and produce an aryl cation and fluoride anion; and (c) deprotonation and rearomatization of the arene ring by the fluoride anion to form the imidated product and HF.

(5) The utilization of these mechanistic findings revealed that the limited reactivity of benzene should be attributed to its lower ability to be oxidized by the imidyl radical in the turnover-limiting **SET1** step. We predicted that electron-rich substrates will be relatively easily oxidized by the imidyl radical and, consequently, will be more reactive toward C–H imidation, which is consistent with our previously reported experiments.^33^

(6) We developed a simple computational tool for predicting the regioselectivity for imidation and demonstrated that C–N bond formation is the regioselectivity-determining step. Subsequent experiments confirmed our computational predictions and validated the predictive power of this simple regioselectivity tool.

In summary, the atomistic-level understanding gained from our joint computational and experimental studies, presented here for the Cu-catalyzed aromatic C–H imidation by NFSI, will not only be instrumental for the development of the next generation of novel catalysts and ligands for selective aromatic C–H imidation, but also open many prospects for the design of novel, efficient, and highly selective reactions with earth-abundant transition metal catalysts and other nitrogen radical sources.

## Experimental

### Computational and experimental procedures

#### Computational methodology

Geometry optimizations and frequency calculations of all presented structures were performed with the Gaussian-09 suite of programs^88^ at the B3LYP-D3/[6-31G(d,p) + Lanl2dz (Cu, Br, I)] level of theory (below, termed as B3LYP-D3/BS1) with the corresponding Hay–Wadt effective core potentials for Cu, Br and I^89–91^ and Grimme's empirical dispersion-correction for B3LYP.^92^ The frequency analysis is used to characterize each minimum with zero imaginary frequencies and transition state (TS) structures with only one imaginary frequency. Intrinsic reaction coordinate (IRC) calculations were performed for selected TSs to ensure their true nature and to connect proper reactants and products. The calculated Gibbs free energies are corrected to a solution standard state of 1 M at 298.15 K.^55,56^ Bulk solvent effects are incorporated for all calculations (*i.e.* geometry optimization, frequency, and single point energy calculations) using the self-consistent reaction field polarizable continuum model (IEF-PCM)^57–59^ with 1,2-dichloroethane (DCE) as the solvent. The electronic energies were re-computed at the B3LYP-D3/[6-311+G(d,p) + SDD (Cu, Br, and I)] level of theory (below, termed as B3LYP-D3/BS2) with the corresponding effective core potentials for Cu, Br and I.^93–95^ The thermal corrections for the free energy and enthalpy are calculated at the B3LYP-D3/BS1 level. These corrections were then applied to the energies calculated at the B3LYP-D3/BS2 level to afford the reported free energy and enthalpy values. Because of the clear importance of entropy on the reaction profiles, we focus the discussion on the free energies in the text.

The electronic states of each transition state and intermediate were carefully analyzed. It was found that many of the TSs and intermediates on the singlet potential energy surface have lower energy open-shell singlet electronic states. In these cases, we re-calculated the geometries and energies of the structures at their open-shell singlet electronic states using unrestricted DFT (UB3LYP-D3).^96,97^ The presented Hirshfeld charges^98–100^ were computed at the B3LYP-D3/BS2 level of theory.

#### Experimental methodology

Unless otherwise noted, all reactants or reagents including dry solvents were obtained from commercial suppliers and used as received. CuBr was purchased from Nacalai Tesque, Inc. NFSI and 6,6′-Me_2_bpy were purchased from TCI. Anhydrous 1,2-dichloroethane was purchased from Kanto Chemical Co., Inc. All reactions were performed with dry solvents under an atmosphere of N_2_ gas in flame-dried glassware using standard vacuum-line techniques. All work-up and purification procedures were carried out with reagent-grade solvents in air. For kinetic experiments using *in situ* IR, the reaction spectra were recorded using an IC 15 from Mettler-Toledo AutoChem. Data processing was carried out using Microsoft® Excel® for Mac v. 14.2.5.

Experimental procedures are as follows: a three-necked reaction vessel equipped with a magnetic stirring bar was dried with a heat gun. CuX, where X = Cl, Br and I, (0.060 mmol, 10 mol%), 6,6′-Me_2_bpy (13 mg, 0.072 mmol, 12 mol%), NFSI (199 mg, 0.63 mmol, 1.05 equiv.), and triphenylene (69 mg, 0.3 mmol; an internal standard for ^1^H NMR analysis) were added to the vessel. The IR probe was inserted through an adapter into the middle neck; another neck was capped by a rubber septum for the purpose of reagent injection, and the third was jointed with a three-way cock in order to flow N_2_ gas. This vessel was evacuated and purged with N_2_ three times. DCE (2 mL) was then added to the vessel and the mixture was heated at 70 °C in an oil bath. After stirring the mixture for 6 min, **1** (0.6 M, DCE solution, 1 mL) was added to the vessel *via* a syringe and at this point the data collection was started. *In situ* IR spectra were recorded over the course of the reaction.

Experimental procedures for imidation of 2-(9-anthryl)-thiophene (**3**) and 4-phenyl-2-(thiophen-2-yl)oxazole (**4**) are as follows: to a Schlenk tube were added NFSI (66 mg, 0.21 mmol, 1.05 equiv.), CuBr (2.8 mg, 0.020 mmol, 10 mol%) and 6,6′-Me_2_bpy (4.4 mg, 0.024 mmol, 12 mol%) in open air. The tube was filled with N_2_ by employing a usual Schlenk evacuate–refill cycle technique. DCE (1.0 mL) and the starting material (0.20 mmol) were added to the tube and the mixture was heated at 70 °C for 12 h. The mixture was then cooled to 25 °C. The crude solution was filtered through a pad of silica gel and Na_2_SO_4_ and concentrated *in vacuo*. Purification by chromatography on silica gel (hexane/EtOAc = 5 : 1) provided the corresponding imidated product (**3′**, 79.3 mg, 0.14 mmol, 71%; **4′**, 58.8 mg, 0.11 mmol, 56%).

## References

[cit1] Uno S. N., Kamiya M., Yoshihara T., Sugawara K., Okabe K., Tarhan M. C., Fujita H., Funatsu T., Okada Y., Tobita S., Urano Y. (2014). Nat. Chem..

[cit2] Li L. L., Diau E. W. G. (2013). Chem. Soc. Rev..

[cit3] Shirota Y., Kageyama H. (2007). Chem. Rev..

[cit4] Fukui N., Cha W. Y., Lee S., Tokuji S., Kim D., Yorimitsu H., Osuka A. (2013). Angew. Chem., Int. Ed..

[cit5] Hartwig J. F. (2008). Acc. Chem. Res..

[cit6] Ley S. V., Thomas A. W. (2003). Angew. Chem., Int. Ed..

[cit7] Ma D. W., Cai Q. A. (2008). Acc. Chem. Res..

[cit8] Surry D. S., Buchwald S. L. (2008). Angew. Chem., Int. Ed..

[cit9] Surry D. S., Buchwald S. L. (2010). Chem. Sci..

[cit10] Valente C., Calimsiz S., Hoi K. H., Mallik D., Sayah M., Organ M. G. (2012). Angew. Chem., Int. Ed..

[cit11] Jiao J., Murakami K., Itami K. (2016). ACS Catal..

[cit12] Shin K., Kim H., Chang S. (2015). Acc. Chem. Res..

[cit13] Lyons T. W., Sanford M. S. (2010). Chem. Rev..

[cit14] Kaliappan K. P., Subramanian P., Rudolf G. C. (2016). Chem.–Asian J..

[cit15] Thansandote P., Lautens M. (2009). Chem.–Eur. J..

[cit16] Dong X., Liu Q., Dong Y., Liu H. (2016). Chem.–Eur. J..

[cit17] Segawa Y., Maekawa T., Itami K. (2015). Angew. Chem., Int. Ed..

[cit18] Wencel-Delord J., Glorius F. (2013). Nat. Chem..

[cit19] Yamaguchi J., Yamaguchi A. D., Itami K. (2012). Angew. Chem., Int. Ed..

[cit20] Romero N. A., Margrey K. A., Tay N. E., Nicewicz D. A. (2015). Science.

[cit21] Shang M., Sun S. Z., Dai H. X., Yu J. Q. (2014). J. Am. Chem. Soc..

[cit22] Yoo E. J., Ma S., Mei T. S., Chan K. S. L., Yu J. Q. (2011). J. Am. Chem. Soc..

[cit23] Kim H., Chang S. (2016). ACS Catal..

[cit24] Roane J., Daugulis O. (2016). J. Am. Chem. Soc..

[cit25] Raghuvanshi K., Zell D., Rauch K., Ackermann L. (2016). ACS Catal..

[cit26] Allen L. J., Cabrera P. J., Lee M., Sanford M. S. (2014). J. Am. Chem. Soc..

[cit27] Antonchick A. P., Samanta R., Kulikov K., Lategahn J. (2011). Angew. Chem., Int. Ed..

[cit28] Kawano T., Hirano K., Satoh T., Miura M. (2010). J. Am. Chem. Soc..

[cit29] Wang Q., Schreiber S. L. (2009). Org. Lett..

[cit30] Foo K., Sella E., Thome I., Eastgate M. D., Baran P. S. (2014). J. Am. Chem. Soc..

[cit31] Boursalian G. B., Ham W. S., Mazzotti A. R., Ritter T. (2016). Nat. Chem..

[cit32] Boursalian G. B., Ngai M. Y., Hojczyk K. N., Ritter T. (2013). J. Am. Chem. Soc..

[cit33] Kawakami T., Murakami K., Itami K. (2015). J. Am. Chem. Soc..

[cit34] Do Amaral L., Bull H. G., Cordes E. H. (1972). J. Am. Chem. Soc..

[cit35] Nakane R., Kurihara O., Takematsu A. (1971). J. Org. Chem..

[cit36] Olah G. A. (1971). Acc. Chem. Res..

[cit37] Olah G. A., Kuhn S. J., Flood S. H. (1961). J. Am. Chem. Soc..

[cit38] Pitts C. R., Bloom S., Woltornist R., Auvenshine D. J., Ryzhkov L. R., Siegler M. A., Lectka T. (2014). J. Am. Chem. Soc..

[cit39] Lal G. S., Pez G. P., Syvret R. G. (1996). Chem. Rev..

[cit40] Engle K. M., Mei T. S., Wang X. S., Yu J. Q. (2011). Angew. Chem., Int. Ed..

[cit41] Liang T., Neumann C. N., Ritter T. (2013). Angew. Chem., Int. Ed..

[cit42] Bobbio C., Gouverneur V. (2006). Org. Biomol. Chem..

[cit43] Champagne P. A., Desroches J., Hamel J. D., Vandamme M., Paquin J. F. (2015). Chem. Rev..

[cit44] Iglesias A., Alvarez R., de Lera A. R., Muñiz K. (2012). Angew. Chem., Int. Ed..

[cit45] Halperin S. D., Fan H., Chang S., Martin R. E., Britton R. (2014). Angew. Chem., Int. Ed..

[cit46] Halperin S. D., Kwon D., Holmes M., Regalado E. L., Campeau L. C., DiRocco D. A., Brittont R. (2015). Org. Lett..

[cit47] Nodwell M. B., Bagai A., Halperin S. D., Martin R. E., Knust H., Britton R. (2015). Chem. Commun..

[cit48] Rueda-Becerril M., Sazepin C. C., Leung J. C. T., Okbinoglu T., Kennepohl P., Paquin J. F., Sammis G. M. (2012). J. Am. Chem. Soc..

[cit49] West J. G., Bedell T. A., Sorensen E. J. (2016). Angew. Chem., Int. Ed..

[cit50] Kaneko K., Yoshino T., Matsunaga S., Kanai M. (2013). Org. Lett..

[cit51] Ni Z. K., Zhang Q., Xiong T., Zheng Y. Y., Li Y., Zhang H. W., Zhang J. P., Liu Q. (2012). Angew. Chem., Int. Ed..

[cit52] Zhang B., Studer A. (2014). Org. Lett..

[cit53] Zhang H. W., Pu W. Y., Xiong T., Li Y., Zhou X., Sun K., Liu Q., Zhang Q. (2013). Angew. Chem., Int. Ed..

[cit54] Zhang H. W., Song Y. C., Zhao J. B., Zhang J. P., Zhang Q. (2014). Angew. Chem., Int. Ed..

[cit55] Essafi S., Tomasi S., Aggarwal V. K., Harvey J. N. (2014). J. Org. Chem..

[cit56] Plata R. E., Singleton D. A. (2015). J. Am. Chem. Soc..

[cit57] Cances E., Mennucci B., Tomasi J. (1997). J. Chem. Phys..

[cit58] Mennucci B., Tomasi J. (1997). J. Chem. Phys..

[cit59] Scalmani G., Frisch M. J. (2010). J. Chem. Phys..

[cit60] Liu Q. L., Yuan Z. L., Wang H. Y., Li Y., Wu Y. C., Xu T., Leng X. B., Chen P. H., Guo Y. L., Lin Z. Y., Liu G. S. (2015). ACS Catal..

[cit61] Jones G. O., Liu P., Houk K. N., Buchwald S. L. (2010). J. Am. Chem. Soc..

[cit62] Yu H. Z., Jiang Y. Y., Fu Y., Liu L. (2010). J. Am. Chem. Soc..

[cit63] Lefevre G., Franc G., Tlili A., Adamo C., Taillefer M., Ciofini I., Jutand A. (2012). Organometallics.

[cit64] Zhang S. L., Bie W. F., Huang L. (2014). Organometallics.

[cit65] Zhang S. L., Fan H. J. (2013). Organometallics.

[cit66] The study of the exact mechanism of this process, which could occur *via* stepwise (complexation-then-oxidation) or concerted pathways, is beyond scope of this paper

[cit67] Breitenfeld J., Ruiz J., Wodrich M. D., Hu X. L. (2013). J. Am. Chem. Soc..

[cit68] Powers D. C., Benitez D., Tkatchouk E., Goddard W. A., Ritter T. (2010). J. Am. Chem. Soc..

[cit69] Schley N. D., Fu G. C. (2014). J. Am. Chem. Soc..

[cit70] Zhu D., Budzelaar P. H. M. (2010). Organometallics.

[cit71] Dissociation of the imidyl radical from **6-N-F**, *i.e.*, LCu^II^F[NSI] → LCuIF + ˙NS^I^, is calculated to be unfavorable by Δ*G* = 39.9 kcal mol^–1^. This indicates that the formation of a reactive imidyl radical from **6-N-F** is unlikely

[cit72] Rodriguez-Fortea A., Alemany P., Alvarez S., Ruiz E. (2002). Inorg. Chem..

[cit73] Hefni M. A., Mcconnell N. M., Rietmeijer F. J., Gribnau M. C. M., Keijzers C. P. (1986). Mol. Phys..

[cit74] Jacobson R. R., Tyeklar Z., Karlin K. D., Zubieta J. (1991). Inorg. Chem..

[cit75] Lee S. C., Holm R. H. (1993). Inorg. Chem..

[cit76] Marino N., Armentano D., De Munno G., Cano J., Lloret F., Julve M. (2012). Inorg. Chem..

[cit77] Rietmeijer F. J., Degraaff R. A. G., Reedijk J. (1984). Inorg. Chem..

[cit78] **D7-N-4F** is unstable toward **SET2** on the triplet surface. For the C–N bond forming process, the energies of the triplet and quintet states are very close in energy (see the ESI). Therefore, we expect that the calculations of **D7-N-4F** on the quintet surface will be an adequate approximation of the same structure on the triplet surface

[cit79] Kozuch S., Shaik S. (2011). Acc. Chem. Res..

[cit80] Halevi E. A. (2014). New J. Chem..

[cit81] Halevi E. A. (2015). New J. Chem..

[cit82] Perrin C. L. (2015). New J. Chem..

[cit83] Perrin C. L., Flach A. (2011). Angew. Chem., Int. Ed..

[cit84] Fiksel A. I., Parmon V. N., Zamaraev K. I. (1982). Chem. Phys..

[cit85] Ulstrup J., Jortner J. (1975). J. Chem. Phys..

[cit86] Recently Hirshfeld charges have been successful used in predicting regioselectivity in electrophilic aromatic substitution reactions. See: Liu S. B., J. Phys. Chem. A, 2015, 119 , 3107 –3111 . 25723372 10.1021/acs.jpca.5b00443

[cit87] This might be the case for benzo[*h*]quinolone, where imidation is observed on the middle ring at the positions bearing the second highest amount of positive charge in the calculations. This can be explained by the higher resonance energy of benzene relative to pyridine. See: Fishtik I. Grimme S., Phys. Chem. Chem. Phys., 2012, 14 , 15888 –15896 .23093113

[cit88] Frisch M. J., Trucks G. W., Schlegel H. B., Scuseria G. E., Robb M. A., Cheeseman J. R., Scalmani G., Barone V., Mennucci B., Petersson G. A., Nakatsuji H., Caricato M., Li X., Hratchian H. P., Izmaylov A. F., Bloino J., Zheng G., Sonnenberg J. L., Hada M., Ehara M., Toyota K., Fukuda R., Hasegawa J., Ishida M., Nakajima T., Honda Y., Kitao O., Nakai H., Vreven T., Montgomery Jr J. A., Peralta J. E., Ogliaro F., Bearpark M., Heyd J. J., Brothers E., Kudin K. N., Staroverov V. N., Kobayashi R., Normand J., Raghavachari K., Rendell A., Burant J. C., Iyengar S. S., Tomasi J., Cossi M., Rega N., Millam M. J., Klene M., Knox J. E., Cross J. B., Bakken V., Adamo C., Jaramillo J., Gomperts R., Stratmann R. E., Yazyev O., Austin A. J., Cammi R., Pomelli C., Ochterski J. W., Martin R. L., Morokuma K., Zakrzewski V. G., Voth G. A., Salvador P., Dannenberg J. J., Dapprich S., Daniels A. D., Farkas Ö., Foresman J. B., Ortiz J. V., Cioslowski J. and Fox D. J., Gaussian 09, Revision D.01, Gaussian, Inc., Wallingford CT, 2009.

[cit89] Hay P. J., Wadt W. R. (1985). J. Chem. Phys..

[cit90] Hay P. J., Wadt W. R. (1985). J. Chem. Phys..

[cit91] Wadt W. R., Hay P. J. (1985). J. Chem. Phys..

[cit92] Grimme S., Antony J., Ehrlich S., Krieg H. (2010). J. Chem. Phys..

[cit93] Andrae D., Haussermann U., Dolg M., Stoll H., Preuss H. (1990). Theor. Chim. Acta.

[cit94] Igelmann G., Stoll H., Preuss H. (1988). Mol. Phys..

[cit95] Vonszentpaly L., Fuentealba P., Preuss H., Stoll H. (1982). Chem. Phys. Lett..

[cit96] Marell D. J., Furan L. R., Woods B. P., Lei X., Bendelsmith A. J., Cramer C. J., Hoye T. R., Kuwata K. T. (2015). J. Org. Chem..

[cit97] Zou L. F., Paton R. S., Eschenmoser A., Newhouse T. R., Baran P. S., Houk K. N. (2013). J. Org. Chem..

[cit98] Hirshfeld F. L. (1977). Theor. Chim. Acta.

[cit99] Ritchie J. P. (1985). J. Am. Chem. Soc..

[cit100] Ritchie J. P., Bachrach S. M. (1987). J. Comput. Chem..

