# Supplementary material for: Cu-Catalyzed aromatic C–H imidation with N-fluorobenzenesulfonimide: mechanistic details and predictive models
Source: Chem Sci. 2016 Oct 19;8(2):988–1001. doi: 10.1039/c6sc04145k (PMC5354063; doi:10.1039/c6sc04145k)
Supplement: Supplementary file 1 [file SC-008-C6SC04145K-s001.pdf]

Electronic Supplementary Material (ESI) for Chemical Science.
This journal is © The Royal Society of Chemistry 2016

Supporting Information

Cu-catalyzed Aromatic C-H Imidation with N-Fluorobenzenesulfonimide: Mechanistic Details and Predictive Models

Brandon E. Haines[†], Takahiro Kawakami[§], Keiko Kuwata,[§] Kei Murakami[§],
Kenichiro Itami*[§,||], and Djamaladdin G. Musaev*[,†]

[†] Cherry L. Emerson Center for Scientific Computation, Emory University, 1515 Dickey Drive, Atlanta, Georgia 30322, United States

[§] Institute of Transformative Bio-Molecules (WPI-ITbM) and Graduate School of Science and

[||] JST-ERATO, Itami Molecular Nanocarbon Project, Nagoya University, Chikusa, Nagoya 464-8602, Japan

E-mail: dmusaev@emory.edu, itami@chem.nagoya-u.ac.jp

Table of Contents

1. $Cu^{I}Br$ oxidation by NFSI S2
2. Details of the Br/F exchange process S3
3. Bimetallic Oxidation of $LCu^{I}X$ (where X = F, Br, Cl, and I) by NFSI S6
4. Conformational Analysis of the Oxidation of **D3-N-3F** S8
5. Analysis of electronic states along the catalytic cycle S9
6. Energy Scan for Deprotonation Step S10
7. Isotope effect calculation S10
8. Validation of FTIR for Reaction Analysis S11
9. Sample FTIR Spectra of Reaction Profile S13
10. Independent Intermolecular KIE Experiments S14
11. Characterization Data, ^{1}H and ^{13}C NMR Spectra S16
12. Energies and Cartesian Coordinates S19

1. LCu^{I}Br oxidation by NFSI

Association of NFSI and the catalyst precursor **1-Br** initiates the generation of the active catalyst through N-coordination (**2-N-Br**) or F-coordination (**2-Br)** of NFSI to the Cu atom (Figure S1). In the two examined oxidation pathways, oxidative insertion (**TS-N-F-Br'**) and S_N2-type displacement (**TS-F-Br'**), the closed shell singlet determinants were found to be unstable using the G09 keyword *stable=opt*. Subsequent re-optimization of the TS structure using unrestricted DFT leads to lower energy TS structures (**TS-N-F-Br** and **TS-F-Br**) with significant diradical character. The lowest energy pathway proceeds through the S_N2-type TS with diradical character, which is 6.5 kcal/mol lower than the oxidative insertion TS (**TS-N-F-Br**) and 2.3 kcal/mol lower than the closed-shell singlet TS (**TS-F-Br'**).

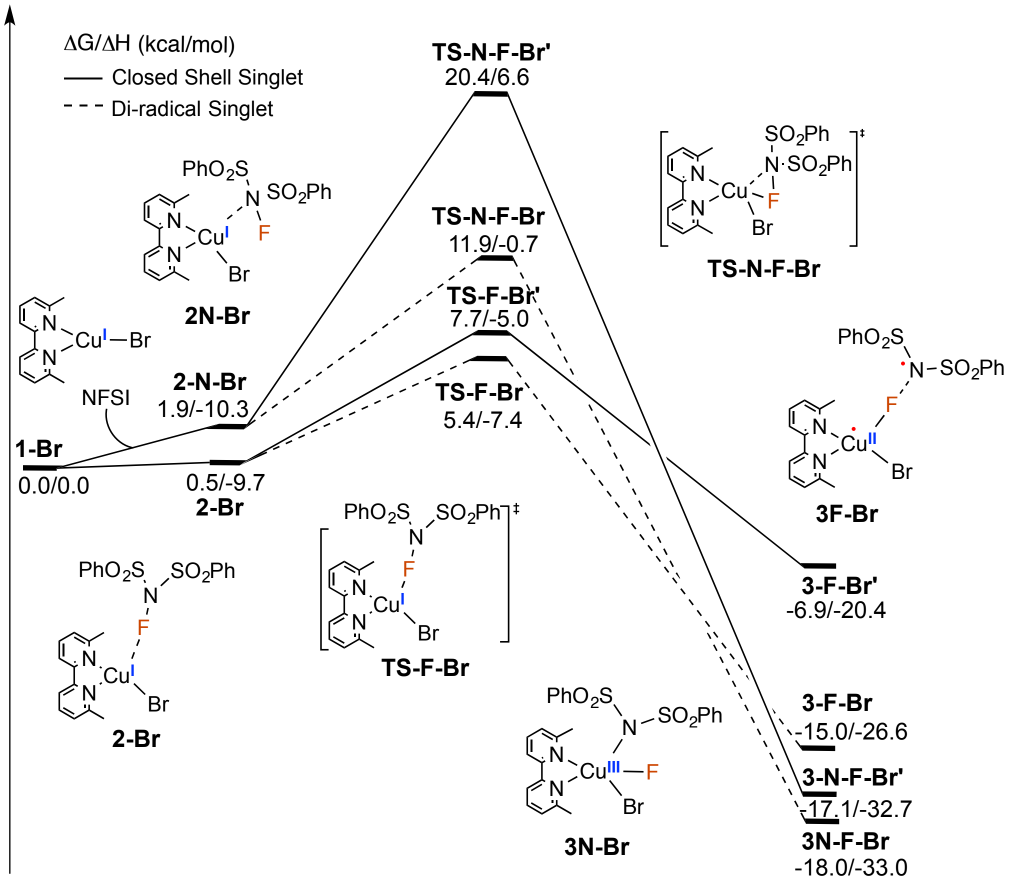

Figure S1. Complete free energy surface for LCu^{I}Br oxidation by NFSI.

On the restricted DFT singlet surface, oxidative insertion of Cu into the N–F bond (**TS-N-F-Br')** proceeds through a symmetric 3-center TS corresponding to a formal oxidative addition (OA) process. In comparison, on the unrestricted DFT singlet surface, **TS-N-F-Br** is asymmetric indicating concerted but asynchronous reactivity for this mode of N–F bond cleavage.[1] (Figure S2) The Cu–N bond is nearly formed (2.15 Å) and the N–F bond is barely broken (1.71 Å) while the Cu–F bond is quite long (2.41 Å). Likewise, on the restricted DFT

singlet surface, S_N2-type TS (**TS-F-Br'**) corresponds to a two-electron oxidation of Cu by a transferring "F^+", whereby the ligand is partially oxidized. This TS structure is late on the N-F bond breaking coordinate, indicating the relative instability of the product complex **3-F-Br'**. Stabilization of the TS and product by the mixed-spin states is consistent with the propensity of the Cu catalyst to favor one-electron oxidation processes.

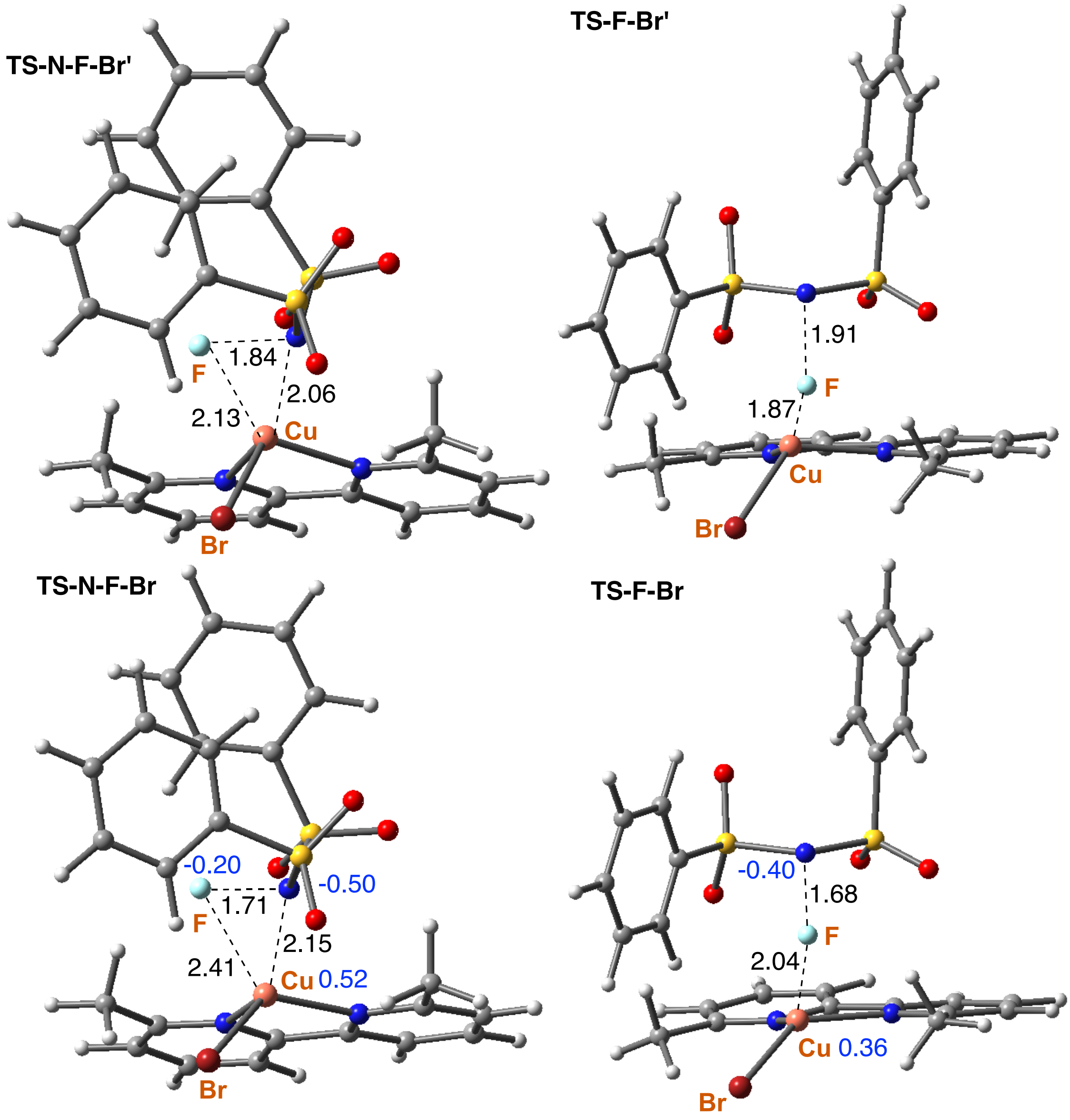

Figure S2. Structures of the transition states for Cu oxidation by NFSI with different electronic states.

1) Marell, D.J.; *et al. J. Org. Chem.* **2015**, *80*, 11744-11754.

2. Details of the Br/F exchange process

After the first oxidation, dinuclear Cu^{II}-Cu^{II} complex **D1-N-F-2Br** and mono-nuclear complexes **6-N-F** and **7-2Br** are in equilibrium. We show that upon reaction with two molecules of NFSI, either side of the equilibrium will produce the dinuclear Cu^{II}-Cu^{II}

complex with bridging fluoride ligands **D3-N-3F** and two molecules of NBrSI (Figure 5). For computational simplicity, we calculated the entire Br/F exchange process starting from the mono-nuclear complex **7-2Br** (Figure S3) and only calculate selected structures starting from the di-nuclear Cu^{II}-Cu^{II} complex **D1-N-F-2Br**.

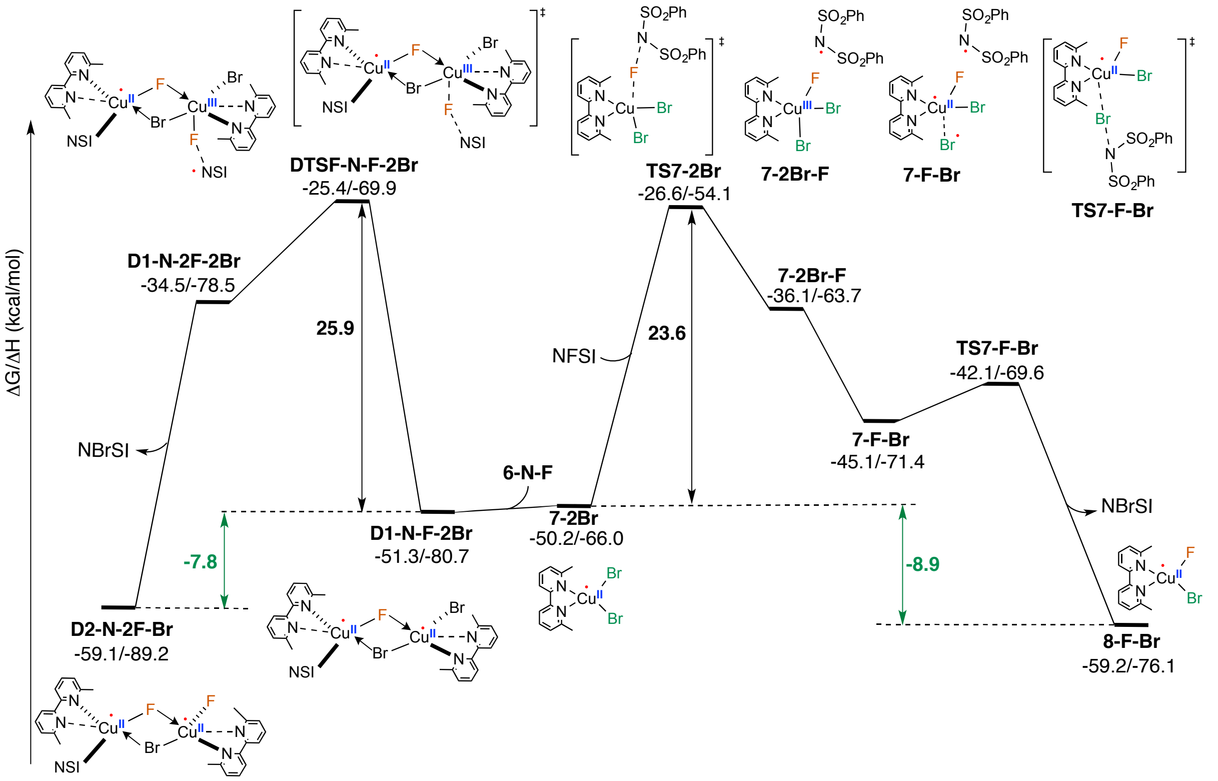

Figure S3. Free energy surface for the first Br/F exchange reaction for the mono-nuclear Cu complex **7-2Br** (right) and the di-nuclear Cu complex **D1-N-F-2Br** (left). Energies are calculated relative to 2 **1-Br** + NFSI.

The calculated barrier for one-electron oxidation of **7-2Br** by NFSI is $\Delta G^{\ddagger}$ = 23.6 kcal/mol indicating that this step is reasonable under the reaction conditions. In the anti-ferromagnetically coupled TS (**TS7-2Br**), the incoming F atom begins to displace one of the Br ligands. This process generates the reactive imidyl radical species: **7-2Br** + NFSI → $LCu^{III}FBr_2$ (**7-2Br-F**) + ·NSI, where NSI = $N(SO_2Ph)_2$, and is endergonic by ΔG = 14.1 kcal/mol. However, the Cu^{III} intermediate **7-2Br-F** is unstable toward dissociation of bromine radical that reduces the Cu^{III} center to Cu^{II}. The resulting $LCu^{II}BrF$ complex (**7-F-Br**) contains both Br and imidyl radicals, and its formation is exergonic from **7-2Br-F** by ΔG = 9.0 kcal/mol. At this stage, it is reasonable to expect that a small amount of the imidyl radical could dissociate and react with arene, but we expect that its majority will react quickly with the bromine radical to form NBrSI. Indeed, imidyl-bromine radical combination to form a new N–Br bond occurs a small barrier of $\Delta G^{\ddagger}$ = 3.0 kcal/mol (**TS7-F-Br**) and falls to a stable N–Br bond and $LCu^{II}BrF$ (**8-F-Br**). This step completes the first Br/F exchange process

($LCuBr_2$ + NFSI → LCuBrF + NBrSI), which is exergonic by ΔG = –8.9 kcal/mol (Figure S3). The calculated barrier for one-electron oxidation of **D1-N-F-2Br** by NFSI is $\Delta G^{\ddagger}$ = 25.9 kcal/mol, and the overall Br/F exchange is exergonic by ΔG = –7.8 kcal/mol. Overall, the energies for the mono-nuclear and di-nuclear pathways are close in energy and either pathway can lead to occurrence of Br/F exchange.

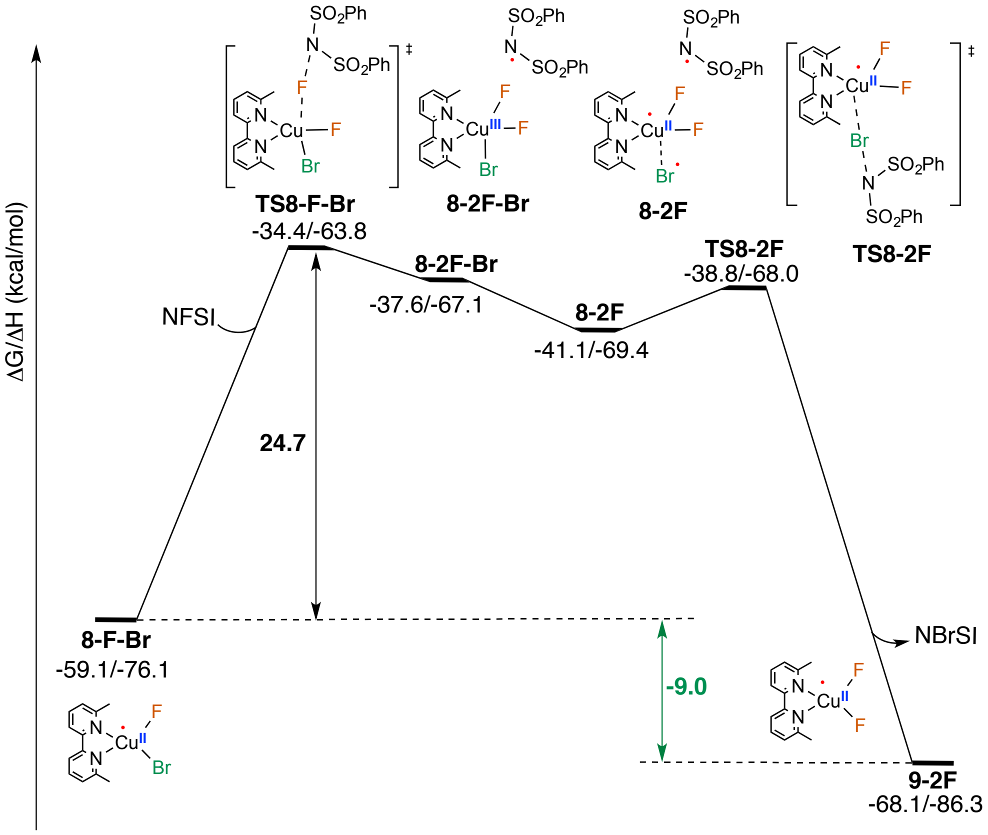

Figure S4. Free energy surface for the second F/Br exchange reaction for the mono-nuclear Cu complex **8-F-Br**. Energies are calculated relative to 2 **1-Br** + NFSI.

The Br/F exchange process can occur a second time when **8-F-Br** reacts with NFSI. For this case, we only examine the energies of the mono-nuclear pathway and assume that the di-nuclear pathway is similar. (Figure S4) The one-electron oxidation of **8-F-Br** by NFSI occurs through a slightly higher barrier (**TS8-F-Br**) than the first exchange process ($\Delta G^{\ddagger}$ = 24.7 kcal/mol). The formation of Cu^{III} complex **8-F-2Br** is endergonic by ΔG = 21.5 kcal/mol. Once again, the Cu^{III} intermediate **8-2F-Br** is unstable toward dissociation of bromine radical that reduces the Cu^{III} center to Cu^{II}. The resulting $LCu^{II}F_2$ complex (**8-2F**) again contains both Br and imidyl radicals, and its formation is exergonic from **8-2F-Br** by ΔG = 3.5 kcal/mol. Then, imidyl-bromine radical combination occurs through a small barrier of $\Delta G^{\ddagger}$ = 2.3 kcal/mol (**TS7-F-Br**) and produces a second molecule of NBrSI and $LCu^{II}F_2$ (**9-2F**). Overall, the second halogen exchange process, LCuBrF + NFSI → $LCuF_2$ + NBrSI, is favorable by

ΔG = –9.0 kcal/mol (Figure S4). Therefore, the overall driving force for the two sequential F/Br exchange reactions with the mono-nuclear complexes is ΔG = –17.9 kcal/mol. Subsequent dinuclear complex formation between **6-N-F** and **9-2F** to form **D3-N-3F** is exergonic by ΔG = –10.7 kcal/mol indicating that **D3-N-3F** will be the major product of the Br/F exchange process regardless of the nuclearity of the reactants.

3. Bimetallic Oxidation of LCu^{I}X (where X = F, Br, Cl, and I) by NFSI

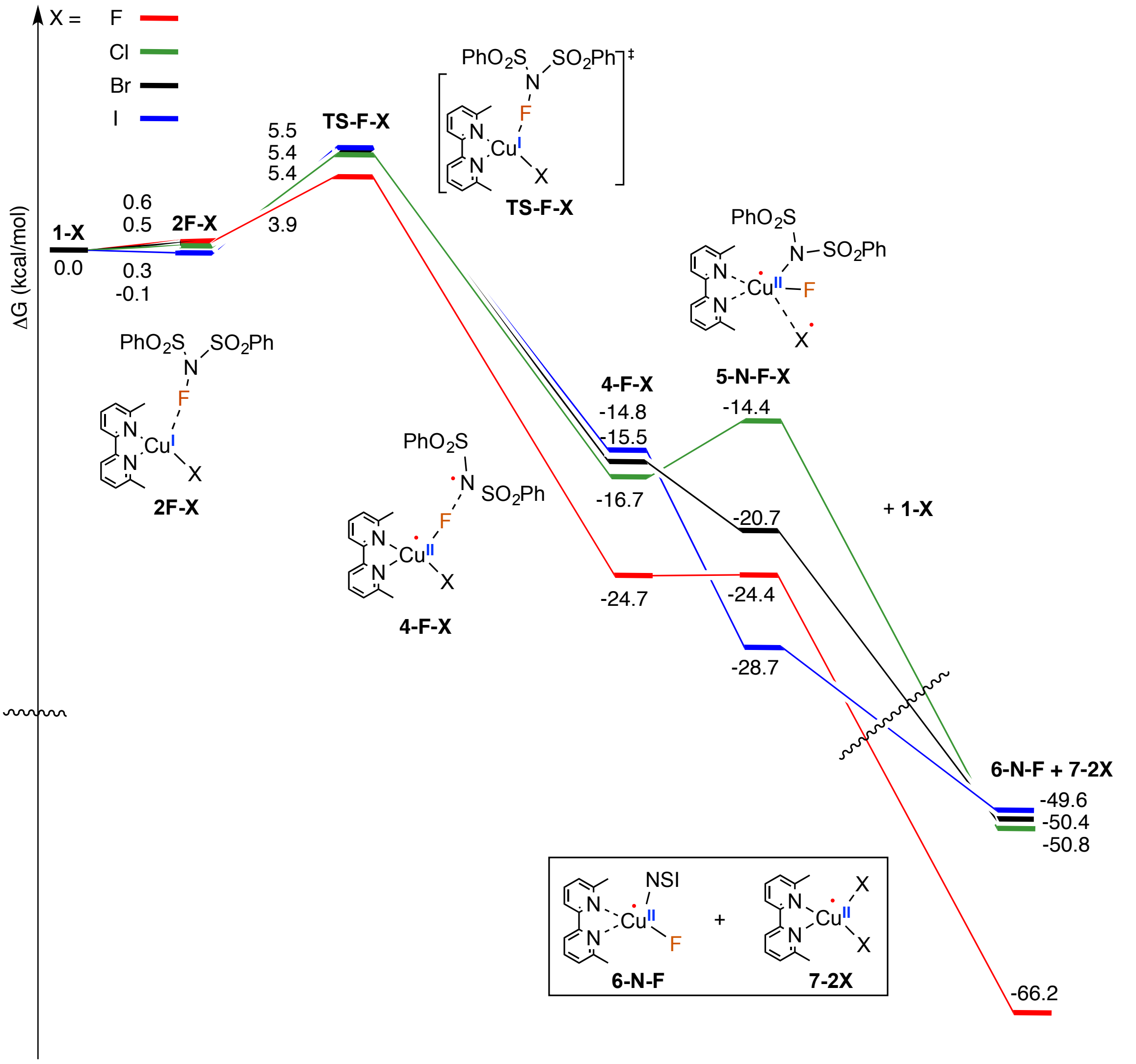

Figure S5. Computed free energy surface for NFSI oxidation of LCu^{I}X catalysts, where X = F, Cl, Br, and I.

The identity of X in the LCu^{I}X pre-catalyst has very little effect on the first oxidation barrier with NFSI (Figure S5). However, the identity of X has a larger effect on the thermodynamic stability of the Cu^{II} intermediates, **4-F-X** and **5-N-F-X**. For X = I and Br, the halogen radical complexes (**5-N-F-I** and **5-N-F-Br**) are lower in energy than the imidyl radical complex (**4-F-I** and **4-F-Br**). Therefore, these are likely more susceptible to the bimetallic oxidation process

described in the text. For X = Cl, **5-N-F-Cl** is higher in energy than **4-F-Cl** by 2.2 kcal/mol indicating that the imidyl radical could react with arene to generate product at this stage, but accessibility to **5-N-F-Cl** will eventually lead to the bimetallic oxidation process. For X = F, **5-N-F-F** and **4-F-F** are the same energy, so both complexes are readily accessible. However, the bimetallic oxidation process might be limited by the extent of radical character on the fluoride ligands in **5-N-F-F** (Figure S6). As the radical character of X and the Cu–X distance increase, **5-N-F-X** will be more susceptible to the bimetallic oxidation process. The calculated trend in radical character of X is I > Br > Cl > F based on the Mulliken spin analysis. The Cu–X distance also follows the same trend increasing by I (3.49 Å) > Br (2.92 Å) > Cl (2.54 Å) > F (1.84 Å).

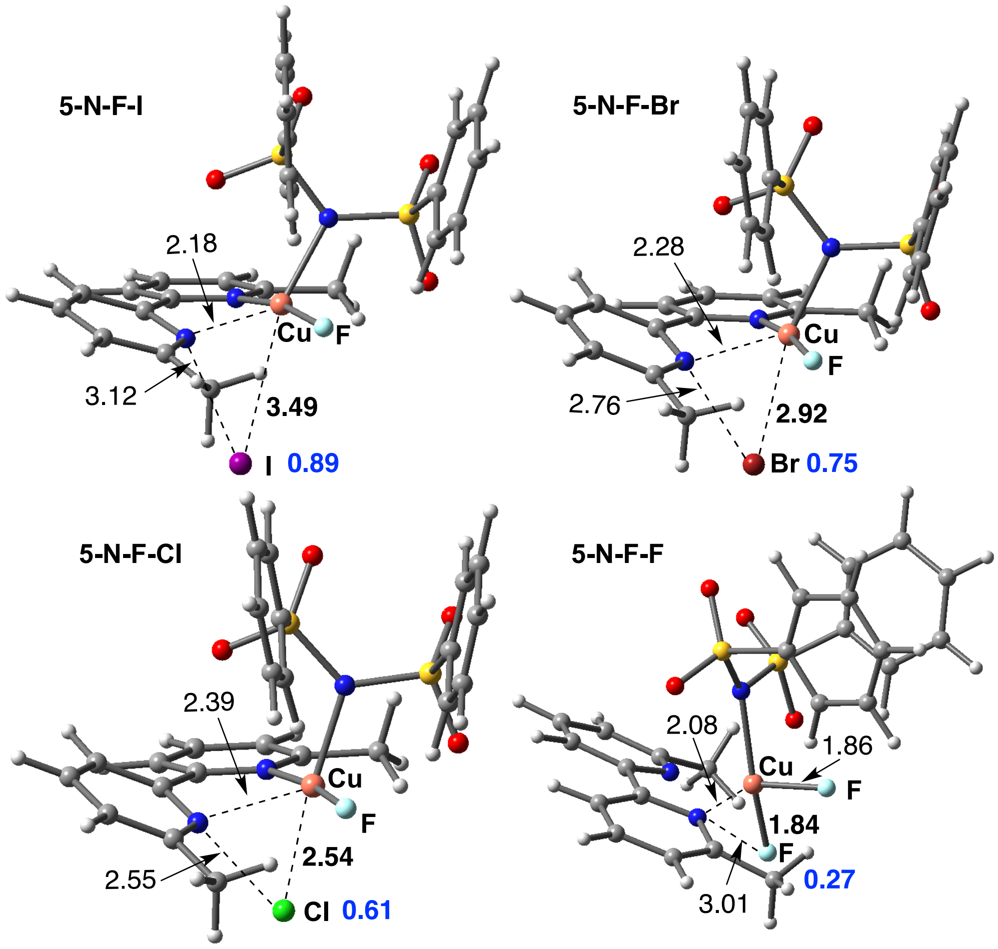

Figure S6. DFT-optimized structures of the Cu^{II}--X radical intermediates, **5-N-F-X**, where X = F, Cl, Br, I. Distances in Å are shown in black and Mulliken spins densities are shown in blue.

However, in all cases, the oxidation of a second molecule of **1-X** ($LCu^{I}X$) is highly exergonic (Figure S5). This indicates that bimetallic oxidation as described in the text will occur regardless of the identity of X, which will then lead to X/F exchange with NFSI and generation of a common active catalyst, **D3-N-3F**. We also investigated the possibility of

molecular bromine formation through radical combination of two Br radicals from two molecules of **5-N-F-Br** (2 **5-N-F-Br** → 2 **6-N-F** + Br_2). While we find that this reaction also highly favorable (ΔG = -20.0 kcal/mol), it is not as exergonic as the bimetallic oxidation reaction of **5-N-F-Br** + **1-Br** → **6-N-F** + **7-2Br** (ΔG = -29.7 kcal/mol). Therefore, we expect that the bimetallic oxidation process is more likely to occur.

4. Conformational Analysis of the Oxidation of D3-N-3F

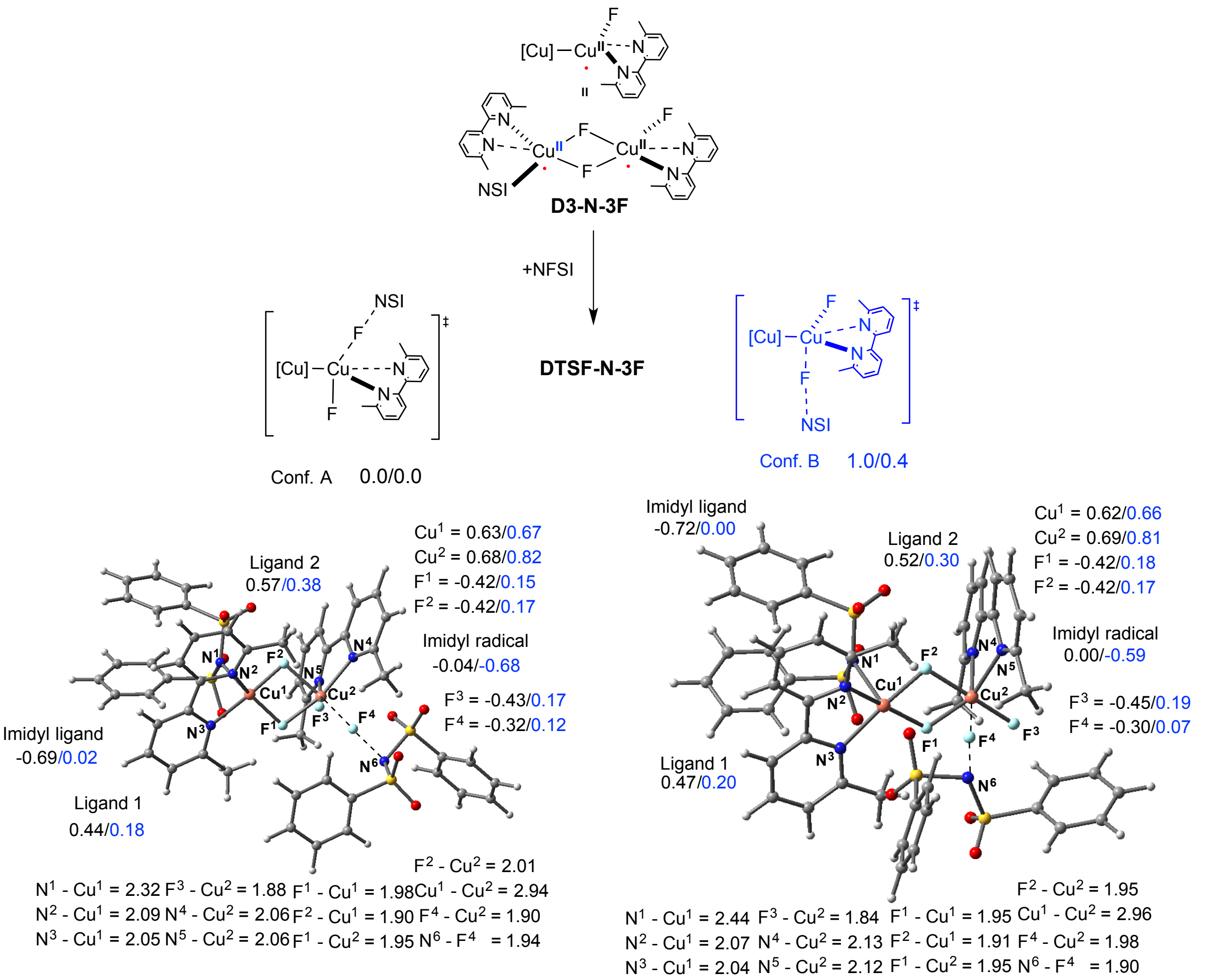

Figure S7.

Two conformational isomers were located for **DTSF-N-3F** as shown in Figure S7. The isomers are close in energy, but Conf. A is lower by 1.0 kcal/mol relative to Conf. B. The isomers differ in the way that NFSI approaches the Cu^{II} center: In Conf. A, the NFSI approaches along the basal plane and in Conf. B, NFSI approaches from below the basal plane. Because it is lower in energy, we discuss Conf. A in the text.

5. Analysis of electronic states along the catalytic cycle

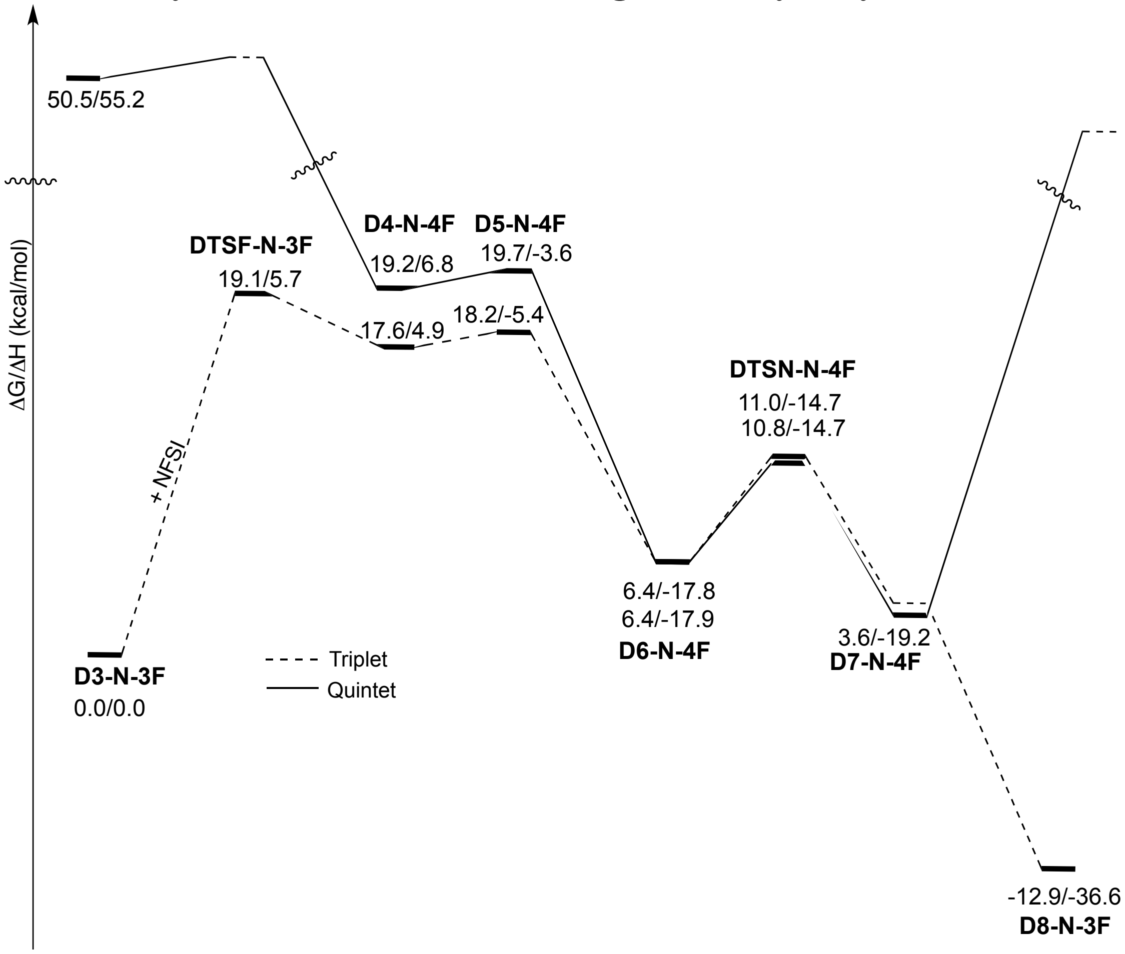

Figure S8. Analysis of the triplet and quintet electronic states during the catalytic cycle.

Along the catalytic cycle the anti-ferromagnetically-coupled triplet state and ferromagentically-coupled quintet states are close in energy (Figure S8). For the reactive imidyl radical, **D4-N-3F**, the anti-ferromagnetic coupling is between the imidyl radical and the dinuclear Cu fragment and the analogous structure on the quintet surface is higher in energy by ΔG = 1.6 kcal/mol. In the C–N bond formation process, the electronic states of **D6-N-4F** and **TSCN-N-4F** are energetically indistinguishable. However, triplet state calculations for **D7-N-F** converge to the much more stable **D8-N-3F** complex, which corresponds with SET from the aryl radical to the dinuclear Cu complex. For the remainder of the catalytic cycle (not shown) the quintet state will be higher in energy than the triplet state because the spin separation in the quintet state will have to take place on the dinuclear Cu complex. This is unfavorable by ΔG = 50.5 kcal/mol in complex **D3-N-3F**.

6. Energy Scan for Deprotonation Step

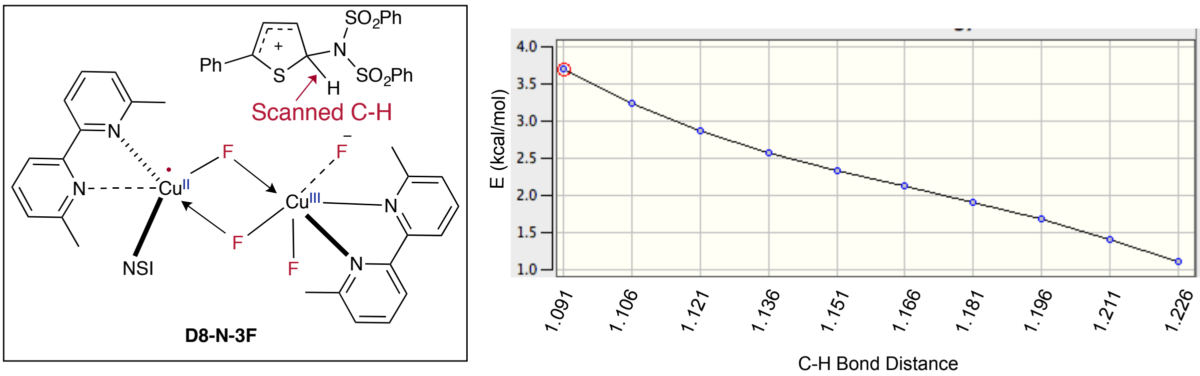

Figure S9. Energy scan of the C-H bond breaking coordinate from **D8-N-3F** for deprotonation and rearomatization of the substrate.

Because **D8-N-3F** could not be located without constraints, it indicates that the subsequent deprotonation and rearomatization step is barrierless. To investigate this further we performed an energy scan of the C-H bond breaking coordinate (Figure S9). Indeed, the process of breaking the C-H bond to form the product is downhill in energy along the coordinate. Additionally, all attempts to locate a TS resulted in convergence to the product complex **D9-N-3F**.

7. Isotope effect calculation

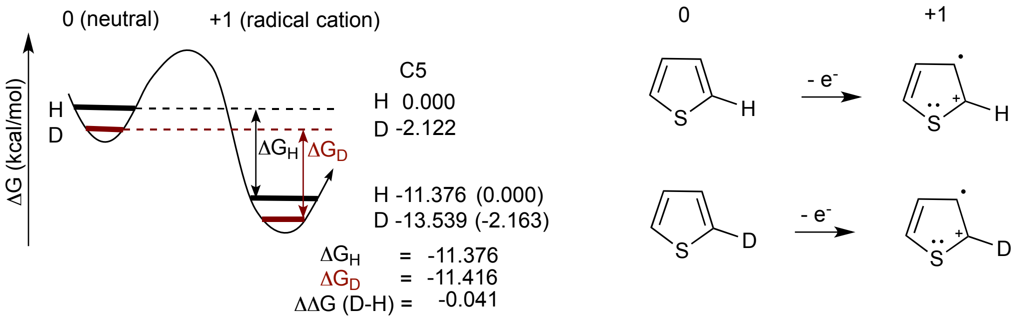

Figure S10. Calculation (left) and rationalization (right) of the difference in free energy for H and D isotopomers at the 5 position of 2-phenylthiophene

The free energy difference between the neutral and oxidized 2-phenylthiophene substrates (in the presence of the dinuclear Cu catalysts) with H at the 5-position is $\Delta G_H = -11.376$ kcal/mol and with D is $\Delta G_D = -11.416$ kcal/mol. (Figure S10) Therefore, the reaction with deuterium is more exergonic than the reaction with hydrogen by $\Delta G_{(D-H)} = -0.054$ kcal/mol. By examining the energy difference between the H and D isotopomers in the neutral molecule ($\Delta G = -2.107$ kcal/mol) and in the radical cation ($\Delta G = -2.161$ kcal/mol), we can conclude that the deuterium stabilizes the oxidized substrate relative to the hydrogen. This can

be rationalized by considering that deuterium is more inductively donating than hydrogen and therefore better stabilizes the carbocation of the oxidized substrate.

8. Validation of FTIR for Reaction Analysis[2]

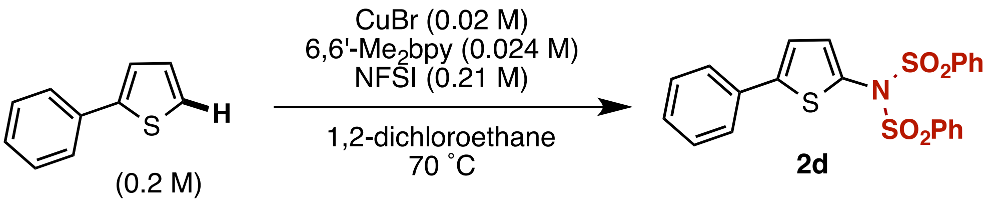

A three-necked reaction vessel equipped with a magnetic stirring bar was dried with a heat gun. CuBr (8.7 mg, 0.060 mmol, 10 mol%), 6,6'-Me_2bpy (13.3 mg, 0.072 mmol, 12 mol%), NFSI (199 mg, 0.63 mmol, 1.05 equiv), and triphenylene (68.5 mmol, 0.3 mmol; an internal standard for ^{1}H NMR analysis) were added to the vessel. The IR probe was inserted through an adapter into the middle neck; another neck was capped by a rubber septum for the purpose of reagent injection, and the third one was jointed three-way cock in order to flow N_2 gas. This vessel was evacuated and purged with N_2 three times. 1,2-Dichloroethane (2 mL) was then added to the vessel and the mixture was heated to 70 °C in an oil bath. After stirring the mixture for 6 min, 2-phenylthiophene (0.6 M, 1,2-dichloroethane solution, 1 mL) was added to the vessel via a syringe and at this point the data collection was started. *In situ* IR spectra were recorded over the course of the reaction. The reaction was intermittently sampled by withdrawal of aliquots (ca. 0.1 mL) from the reaction mixture (30, 60, 120, 180, 240, 480 min). The aliquots were filtered over a pad of silica-gel and the yield was determined by ^{1}H NMR analysis. The time course of the product formation by FITR was in agreement with the NMR sampling method.

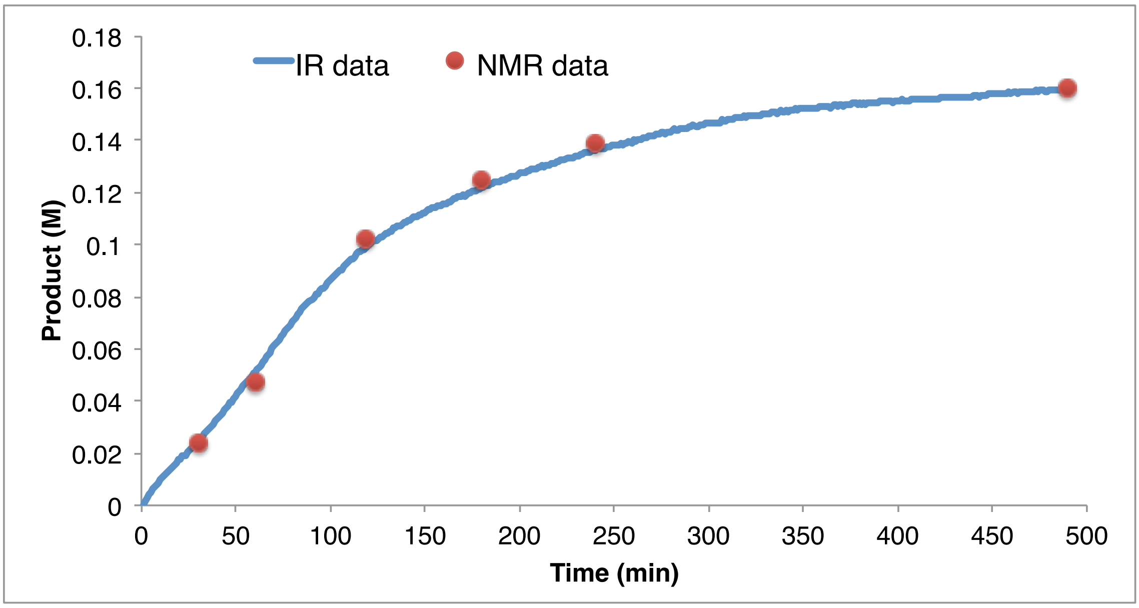

Figure S11. Comparison of conversion data through FTIR (blue line) and ^{1}H NMR reaction sampling (red dots).

2) Kawakami, T.; Murakami, K.; Itami, K. *J. Am. Chem. Soc.* **2015**, *137,* 2460–2463.

9. Sample FTIR Spectra of Reaction Profile[2]

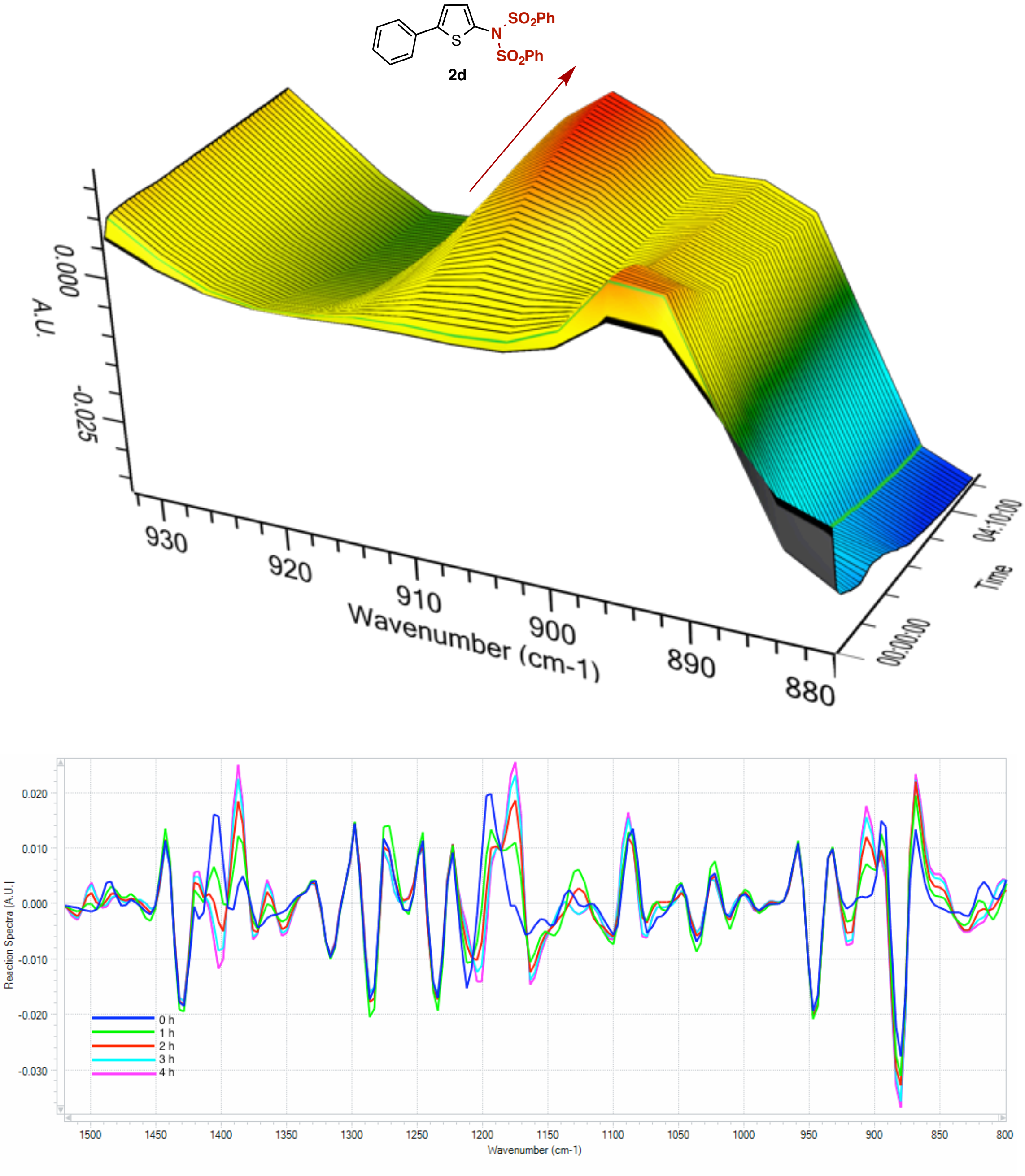

Figure S12. *In situ* IR profiles for the imidation of 2-phenylthiophene with NFSI under copper catalysis.

10. **Independent Intermolecular KIE Experiments**[2]

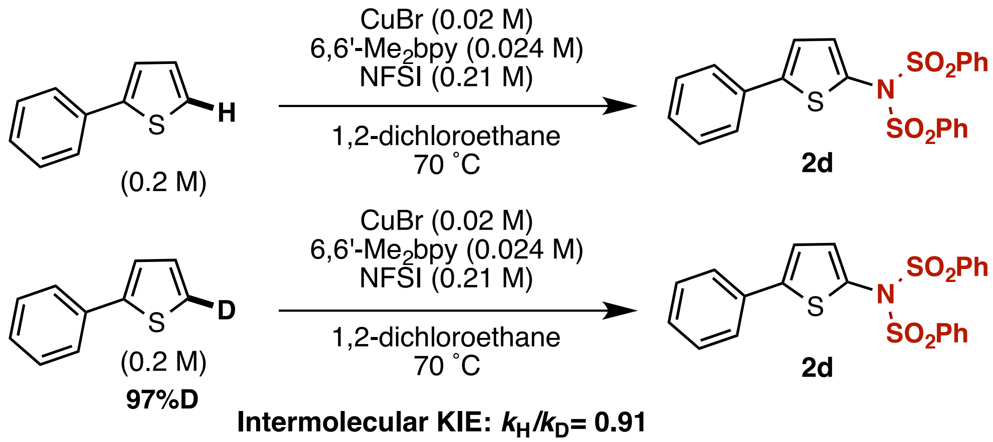

A three-necked reaction vessel equipped with a magnetic stirring bar was dried with a heat-gun. CuBr (8.7 mg, 0.060 mmol, 10 mol%), 6,6'-Me_2bpy (13.3 mg, 0.072 mmol, 12 mol%), and NFSI (199 mg, 0.63 mmol, 1.05 equiv) were added to the vessel. The IR probe was inserted through an adapter into the middle neck; another neck was capped by a rubber septum for the purpose of reagent injection, and the third one was jointed three-way cock in order to flow N_2 gas. This vessel was evacuated and purged with N_2 three times. 1,2-Dichloroethane (2 mL) was then added to the vessel and the mixture was heated to 70 °C in an oil bath. After stirring the mixture for 6 min, 2-phenylthiophene (or 2-deuterio-5-phenylthiophene) (0.6 M, 1,2-dichloroethane solution, 1 mL) was added to the vessel via a syringe and at this point the data collection was started. *In situ* IR spectra were recorded over the course of the reaction. After 4 h, the mixture was cooled to an ambient temperature. Ethyl acetate was the added to dilute the reaction mixture and then benzyl phenyl ether (10 mg, 0.0546 mmol; an internal standard for ^{1}H NMR analysis) was added. The mixture was immediately filtered over a pad of silica-gel and evaporated. The reaction yield was confirmed by ^{1}H NMR analysis. The experiments were performed three times for each substrate and the line plots were made with the average yields of the three runs.

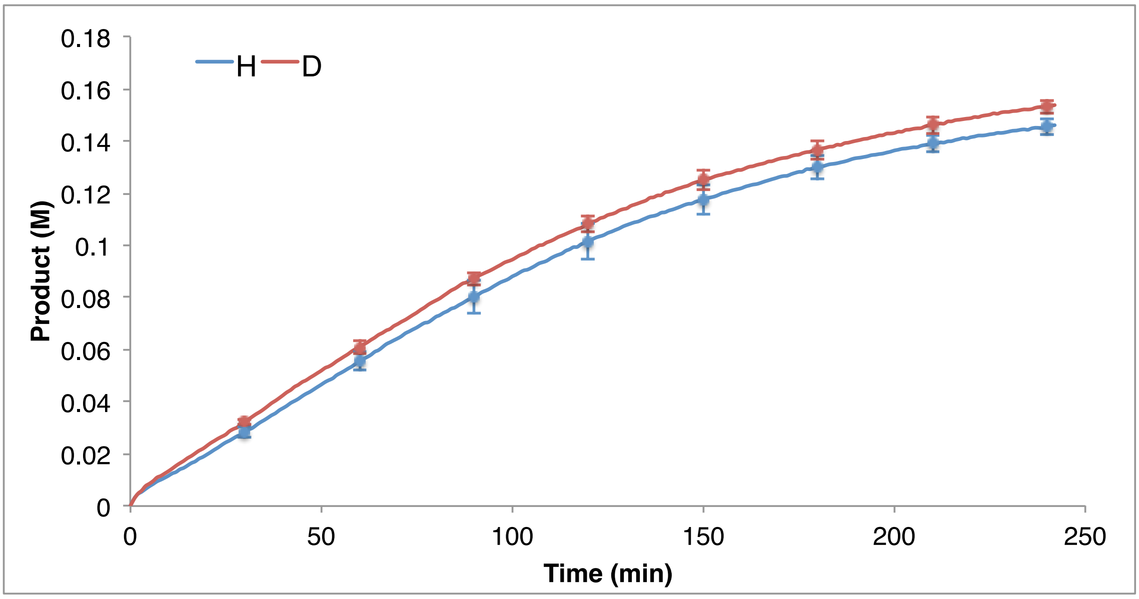

Figure S13. Intermolecular KIE experiments of two substrates. The top and bottom of each error bar indicate the highest and lowest concentration of the product **2d**, respectively. The line plots were made with the average of the three runs.

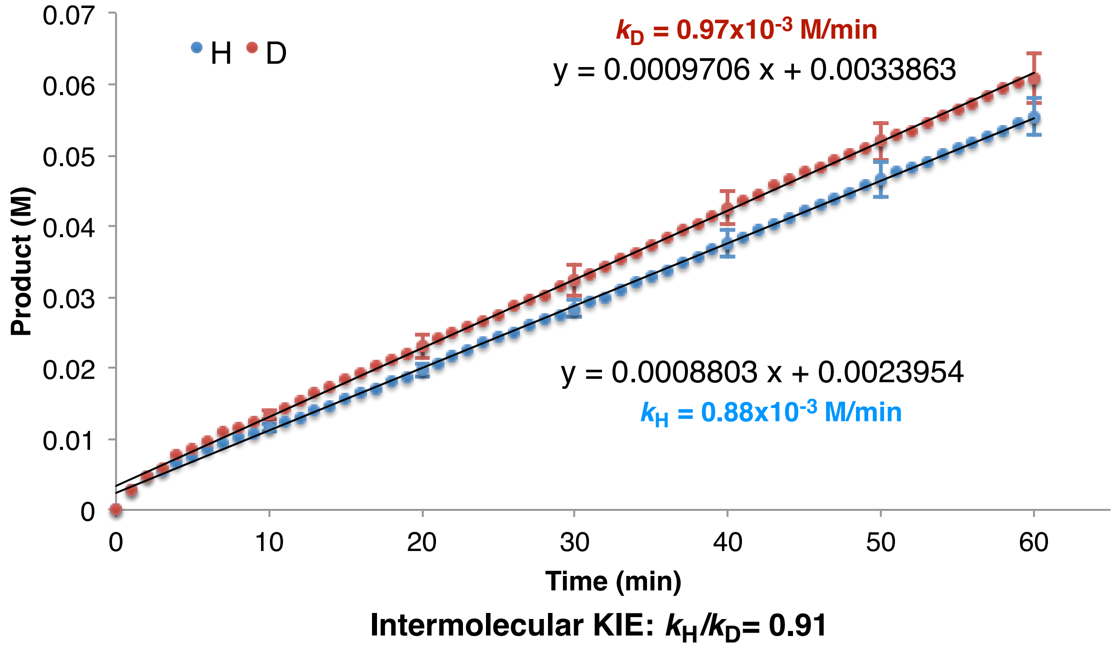

Figure S14. Inset picture of Figure S13. The top and bottom of each error bar indicate the highest and lowest concentration of the product **2d**, respectively. The line plots were made with the average of the three runs.

11. Characterization Data, ^{1}H and ^{13}C NMR Spectra

The high-resolution mass spectra were recorded on Thermo Fisher Scientific Exactive. Nuclear magnetic resonance (NMR) spectra were recorded on a JEOL JNM-ECA-600 (^{1}H 600 MHz, ^{13}C 150 MHz) spectrometer. Chemical shifts for ^{1}H NMR are expressed in parts per million (ppm) relative to tetramethylsilane (δ 0.00 ppm). Chemical shifts for ^{13}C NMR are expressed in ppm relative to $CDCl_3$ (δ 77.2 ppm). Data are reported as follows: chemical shift, multiplicity (s = singlet, d = doublet, dd = doublet of doublets, t = triplet, dt = doublet of triplets, td = triplet of doublets, q = quartet, m = multiplet, brs = broad singlet), coupling constant (Hz), and integration.

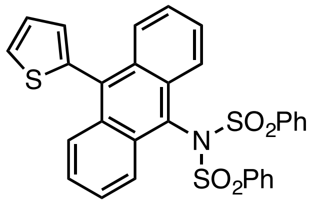

***N*-(Phenylsulfonyl)-*N*-(10-(thiophen-2-yl)anthracen-9-yl)benzenesulfonamide:** ^{1}H NMR ($CDCl_3$) δ 7.15–7.18 (m, 2H), 7.23 (d, *J* = 3.6 Hz, 1H), 7.31–7.33 (m, 3H), 7.50 (t, *J* = 7.8 Hz, 4H), 7.60 (d, *J* = 9.0 Hz, 2H), 7.64 (d, *J* = 5.4 Hz, 1H), 7.70 (t, *J* = 7.8 Hz, 2H), 7.81 (d, *J* = 9.0 Hz, 2H), 7.61 (d, *J* = 12.0 Hz, 4H); ^{13}C NMR ($CDCl_3$) δ124.92, 125.93, 126.58, 127.14, 127.29, 127.40, 128.08, 129.11, 129.98, 131.78, 132.63, 133.36, 134.45, 138.24, 139.44 (one sp^2 signal was not observed because of overlapping); HR-MS (ESI-MS, positive): *m*/*z* = 578.0525. calcd for $C_{30}H_{21}NO_4S_3Na$: 578.0525 [*M* + Na]$^+$.

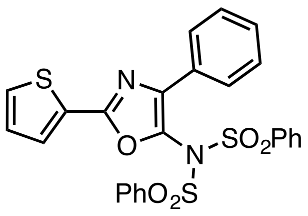

***N*-(4-Phenyl-2-(thiophen-2-yl)oxazol-5-yl)-*N*-(phenylsulfonyl)benzenesulfonamide:** ^{1}H NMR ($CDCl_3$) δ 7.13–7.23 (m, 4H), 7.44 (t, *J* = 7.8 Hz, 4H), 7.51 (d, *J* = 4.2 Hz, 1H), 7.61 (t, *J* = 7.8 Hz, 2H), 7.64 (d, *J* = 4.2 Hz, 1H), 7.67 (d, *J* = 7.8 Hz, 2H), 7.96 (d, *J* = 7.8 Hz, 4H); ^{13}C NMR ($CDCl_3$) δ 127.04, 128.29, 128.57, 129.12, 129.17, 129.23, 129.54, 129.86, 132.67, 134.67, 139.27, 140.92, 157.05 (two sp^2 signals were not observed because of overlapping); HR-MS (ESI-MS, positive): *m*/*z* = 545.0273. calcd for $C_{25}H_{18}N_2O_5S_3Na$: 545.0270 [*M* + Na]$^+$.

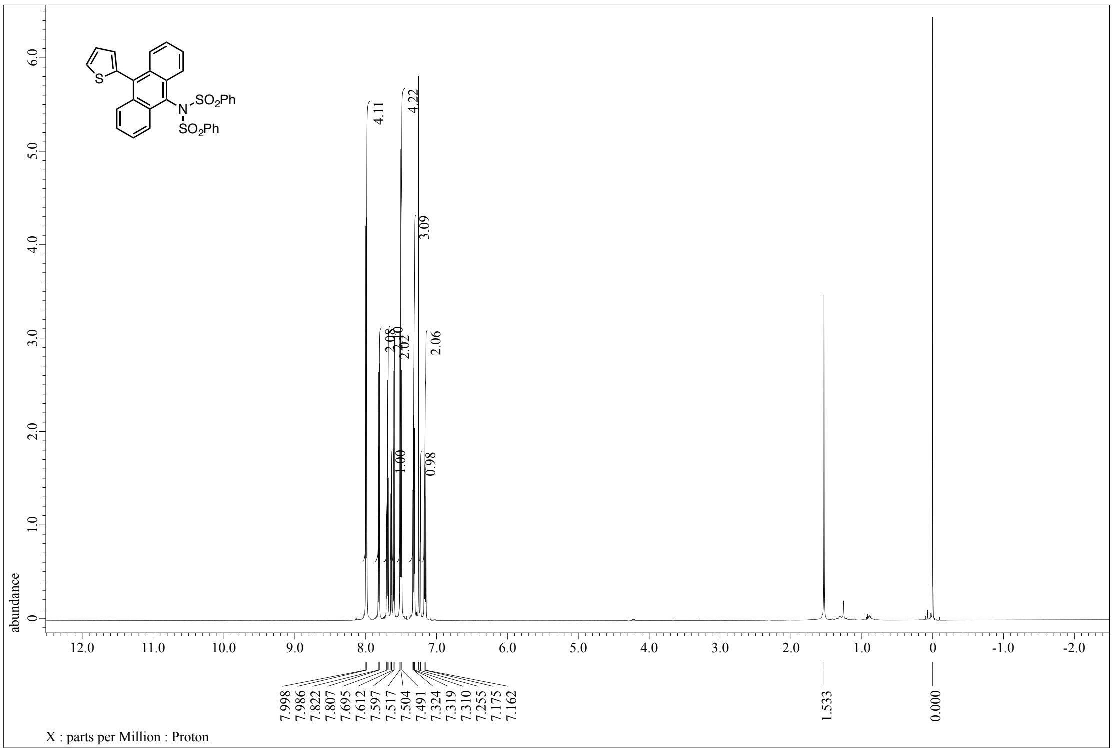

Figure S15. ^{1}H NMR spectrum of *N*-(phenylsulfonyl)-*N*-(10-(thiophen-2-yl)anthracen-9-yl)benzenesulfonamide.

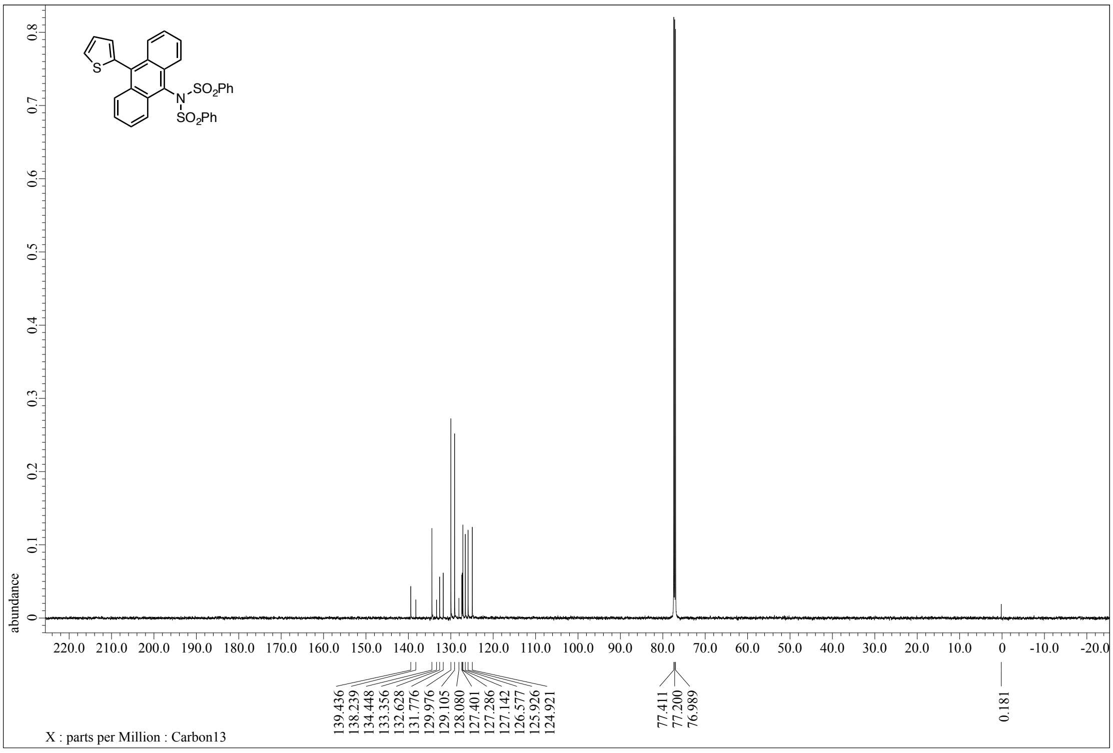

Figure S16. ^{13}C NMR spectrum of *N*-(phenylsulfonyl)-*N*-(10-(thiophen-2-yl)anthracen-9-yl)benzenesulfonamide.

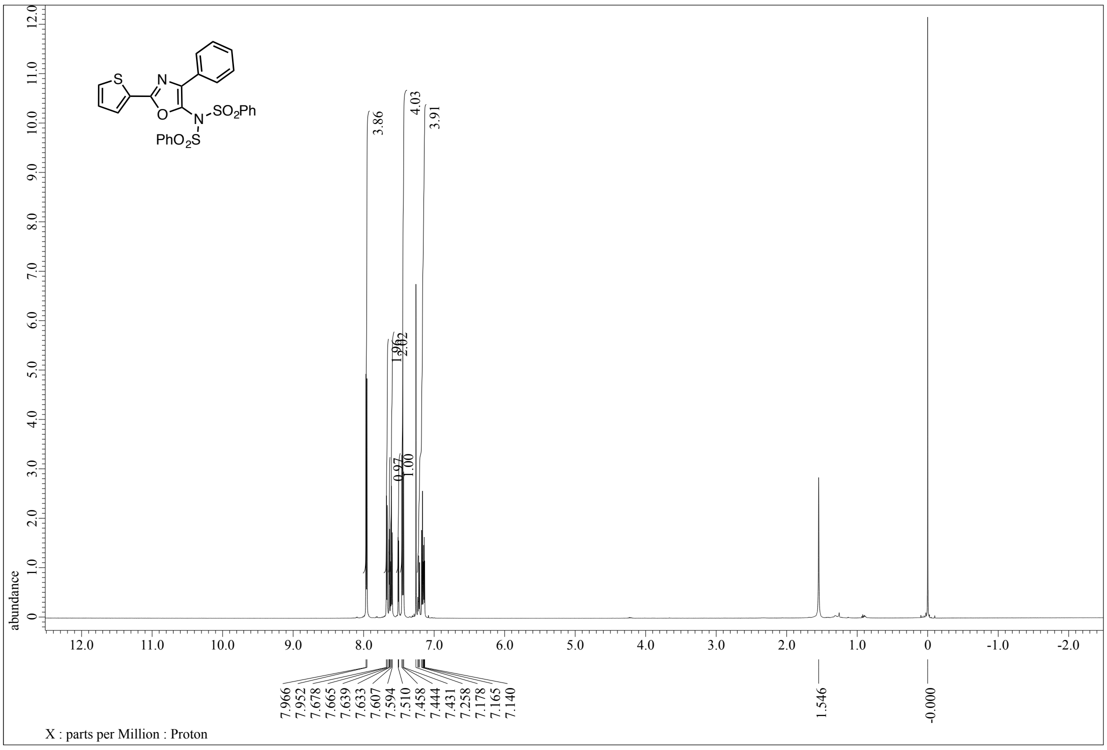

Figure S17. ^{1}H NMR spectrum of *N*-(4-phenyl-2-(thiophen-2-yl)oxazol-5-yl)-*N*-(phenylsulfonyl)benzenesulfonamide.

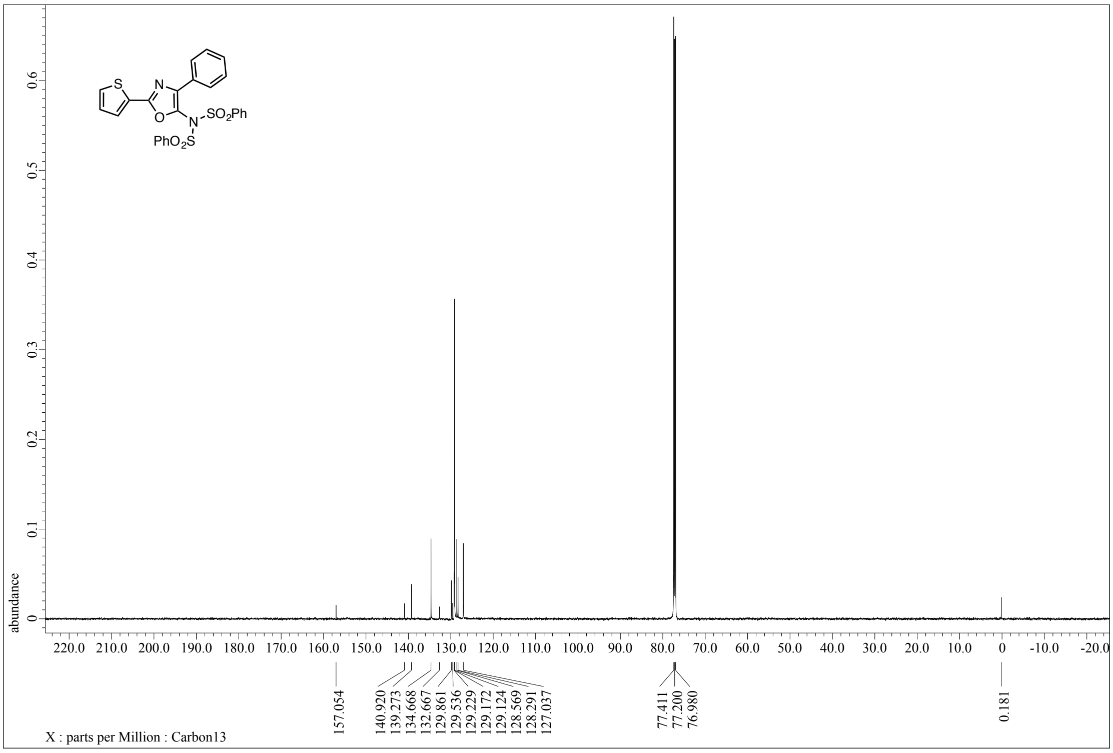

Figure S18. ^{13}C NMR spectrum of *N*-(4-phenyl-2-(thiophen-2-yl)oxazol-5-yl)-*N*-(phenylsulfonyl)benzenesulfonamide.

12. Energies and Cartesian Coordinates

The electronic energies calculated at B3LYP-D3/BS1 [6-31G(d,p) + Lanl2dz (Cu, Br, I)] and B3LYP-D3/BS2 [6-311+G(d,p) + SDD (Cu, Br, and I)] levels of theory are provided, as well as the applied zero point energy (ZPE), enthalpy (H) and free energy (G) corrections calculated at the B3LYP-D3/BS1 level. The provided Cartesian coordinates are generated from geometry optimizations at the B3LYP-D3/BS1 level. Bulk solvent effects are incorporated for all calculations using the self-consistent reaction field polarizable continuum model (IEF-PCM) with 1,2-dichloroethane (DCE) as the solvent.

NFSI

E(BS1) = -1714.996896
E(BS2) = -1715.292232
ZPE = 0.208663
H = 0.227581
G = 0.163274

S -1.42586100 1.63006400 -0.17098700
O -1.68086400 2.34302600 -1.41947500
O -1.34196000 2.31634600 1.11408800
N 0.25134300 0.99259400 -0.40551100
S 0.76289800 -0.39545300 0.64454000
O 0.15304700 -1.62590300 0.14280400
O 0.51929800 0.08780200 2.00274000
C 2.49660400 -0.38266600 0.26411300
C 2.95693600 -1.17066900 -0.79366400
C 3.34198300 0.42975400 1.02410300
C 4.31790800 -1.14128800 -1.09252300
H 2.26893100 -1.79073700 -1.35593900
C 4.69971400 0.44171100 0.71124400
H 2.94548800 1.02506800 1.83851800
C 5.18305300 -0.33865300 -0.34345000
H 4.70155500 -1.74594400 -1.90728100
H 5.37846100 1.05871500 1.29023500
H 6.24167400 -0.32216100 -0.58209200
C -2.47389900 0.20364300 -0.05932400
C -2.81644000 -0.27885900 1.20616600
C -2.85846700 -0.43539200 -1.24124200
C -3.58460100 -1.43880900 1.28195800
H -2.48454200 0.23959600 2.09758600
C -3.62246000 -1.59597400 -1.14252500
H -2.57223500 -0.03019100 -2.20441900
C -3.98190500 -2.09437000 0.11334400
H -3.86988400 -1.83075700 2.25234500
H -3.93692400 -2.11001800 -2.04450800
H -4.57612900 -3.00003500 0.18131000
F 0.25170900 0.42722800 -1.70811600

1-Br
E(BS1) = -783.515223
E(BS2) = -785.058555
ZPE = 0.215459
H = 0.232695
G = 0.167969
Cu -0.88986400 -0.04687500 0.02603900
N 0.74690600 -1.34155600 0.00522800
C 0.66890300 -2.68589500 0.02745400
C 1.82494100 -3.47407600 0.03671400
C 3.06928900 -2.85104300 0.03124300
C 3.13847600 -1.46011300 0.01284300
C 1.94924200 -0.72494700 -0.00421200
N 0.70997400 1.34618900 0.08437500
C 0.59151900 2.68709300 0.08918500
C 1.71793000 3.51008500 -0.03042600
C 2.97416600 2.92557100 -0.15567600
C 3.08636800 1.53698000 -0.15267700
C 1.92592600 0.76769900 -0.02504400
H 1.74088800 -4.55493700 0.05105800
H 4.10213000 -0.96775300 0.01961300
H 4.05889300 1.07387700 -0.25800400
H 1.60094200 4.58807800 -0.02679300
H 3.98011400 -3.44098600 0.04375100
H 3.86105400 3.54281900 -0.25715300
C -0.79307200 3.24954900 0.25161000
H -1.50022200 2.71433400 -0.38844400
H -1.13268700 3.11001100 1.28421000
H -0.82265200 4.31626900 0.01991200
C -0.70961900 -3.28415000 0.05445900
H -1.20593900 -3.03560700 0.99875200
H -1.32225900 -2.85667500 -0.74498600
H -0.68016500 -4.37036700 -0.05170500
Br -3.26862000 0.03319400 -0.14185600

2-Br
E(BS1) = -2498.515745
E(BS2) = -2500.370043
ZPE = 0.425386
H = 0.462376
G = 0.354616
Cu -1.57218200 -0.09349700 0.97903800
N -2.48532800 -0.29441900 -0.92182600
C -2.33239400 -1.32468400 -1.77316400
C -3.15477200 -1.45786800 -2.89827000
C -4.13862700 -0.50412000 -3.13606200
C -4.27748900 0.56835800 -2.25756000
C -3.42168800 0.64956800 -1.15649900
N -2.81392700 1.58627800 0.98079800
C -2.80493900 2.54641100 1.92336900
C -3.49465600 3.74837000 1.73187200
C -4.17792900 3.95500100 0.53688000
C -4.17248600 2.95790300 -0.43529200
C -3.48313200 1.77012200 -0.17612900
C -1.99293500 2.27731800 3.15823700
C -1.21424800 -2.28522400 -1.48639800
Br -0.34707200 -1.51144800 2.48656600
F 0.63600700 0.27443300 -0.83218900
N 1.10385700 1.43342300 -1.50972900
S 1.18903700 2.69021200 -0.22663000
S -0.11743700 1.73332100 -2.83352700
H -3.01438400 -2.29679600 -3.57088400
H -5.04746800 1.31135000 -2.42274200
H -4.67659600 3.11492700 -1.38030300
H -3.48131100 4.50704100 2.50666300
H -4.79464900 -0.59203000 -3.99640600
H -4.70598600 4.88656900 0.35910000
H -2.13551800 1.24495300 3.48958700
H -0.93115800 2.39503300 2.91837500
H -2.24931900 2.96339100 3.96861500
H -0.26722500 -1.81566800 -1.77087500
H -1.16024600 -2.50482000 -0.41608700
H -1.32790000 -3.21506400 -2.04813300
C 0.91492900 2.69441000 -3.91125500
O -0.32437500 0.39294100 -3.37632600
O -1.23804400 2.54062500 -2.34984600
C 2.54881300 2.03335400 0.69958200
O 1.57658200 3.88084600 -0.98504600
O -0.02249100 2.66210700 0.59112100
C 1.88891000 2.03138200 -4.66366000
C 2.68699500 2.78655400 -5.52036300
C 2.50083600 4.16984200 -5.61344300
C 0.71199900 4.07381300 -3.98731800
C 1.51795400 4.81081900 -4.85361900
H 2.01134500 0.95727000 -4.58246500
H 3.45020800 2.29631400 -6.11544600
H 3.12600000 4.75153900 -6.28363300
H -0.04912500 4.54865600 -3.38047900
H 1.37903000 5.88383900 -4.93296100
C 3.84476700 2.44799600 0.37984200
C 4.90370400 1.91291400 1.11108200
C 4.65594200 0.97939100 2.12346600
C 2.27531400 1.10288000 1.70554400
C 3.35052600 0.57447900 2.41796500
H 4.01088300 3.17180700 -0.41008900
H 5.91978600 2.22300500 0.89048500
H 5.48717800 0.56420500 2.68490000
H 1.25962200 0.78724000 1.91728500
H 3.16309000 -0.15527000 3.19862200

TS-F-Br
E(BS1) = -2498.503562
E(BS2) = -2500.363497
ZPE = 0.424088
H = 0.459557
G = 0.355759
$<S^2>$ = 0.1802
732.0170i
Cu -1.49326500 -0.04671500 0.77082900
N -2.68859900 -0.32910500 -0.90112000
C -2.55529300 -1.35226300 -1.76336900
C -3.40570700 -1.46617100 -2.86815100
C -4.39545400 -0.50803300 -3.06235600
C -4.52347800 0.54292500 -2.15662300
C -3.64133100 0.61021100 -1.07564700
N -2.90280600 1.52835500 1.03199500
C -2.89348900 2.44493000 2.01606000
C -3.70461000 3.58414000 1.94248000
C -4.51314300 3.77100500 0.82677000
C -4.51265100 2.81527300 -0.18723800
C -3.69519500 1.69088300 -0.05100200
C -1.95899300 2.21017500 3.16800500

C -1.45262900 -2.33152100 -1.48343300
Br -0.61006000 -1.41866000 2.58377500
F 0.17894800 0.19441500 -0.36559200
N 1.02122300 1.29699500 -1.30733200
S 1.18606700 2.61599200 -0.14669400
S -0.12531300 1.52354800 -2.64531600
H -3.28433800 -2.29395400 -3.55759600
H -5.30208500 1.28240100 -2.29138200
H -5.12922200 2.95615900 -1.06548100
H -3.68762100 4.30918600 2.74869400
H -5.06900000 -0.57842900 -3.91053900
H -5.14056600 4.65265900 0.74292900
H -1.90958300 1.14572200 3.41033300
H -0.95067700 2.52482700 2.87989000
H -2.26241600 2.77740700 4.05073900
H -0.49392200 -1.81312100 -1.56112000
H -1.53749800 -2.71107300 -0.45979900
H -1.47071900 -3.16967300 -2.18249600
C 0.92640500 2.45022100 -3.73817600
O -0.31017400 0.17058000 -3.16895900
O -1.27573200 2.33593300 -2.24115200
C 2.44298700 1.91193200 0.88931200
O 1.73592300 3.71532700 -0.94856600
O -0.04431400 2.80678900 0.62414700
C 1.93012700 1.76821400 -4.43366400
C 2.74630900 2.49514300 -5.29773700
C 2.54933500 3.87097700 -5.45542900
C 0.71432000 3.82365100 -3.87947900
C 1.53667600 4.53209100 -4.75312800
H 2.05927300 0.69968400 -4.30403600
H 3.53188700 1.98872200 -5.84868800
H 3.18850000 4.43101900 -6.13089600
H -0.07072600 4.31474200 -3.31744500
H 1.38826800 5.59885700 -4.88396700
C 3.78255400 2.16583300 0.58179300
C 4.76387200 1.60766900 1.39940900
C 4.39846600 0.80964000 2.48873400
C 2.05487500 1.11975800 1.97167400
C 3.05174900 0.56525900 2.77278200
H 4.04139500 2.78837800 -0.26719400
H 5.81119600 1.79491900 1.18618200
H 5.16883100 0.37532800 3.11842800
H 1.00942800 0.92564900 2.17638500
H 2.77094200 -0.06044300 3.61347500

3-F-Br
E(BS1) = -2498.530511
E(BS2) = -2500.396956
ZPE = 0.425799
H = 0.462328
G = 0.356854
$<S^2>$ = 0.9584
Cu -1.28050600 0.37079600 0.33197800
N -2.89386900 -0.05915700 -1.02173900
C -2.92743900 -0.94389800 -2.03259200
C -4.02972100 -0.98793400 -2.90172800
C -5.09181200 -0.11759300 -2.70977000
C -5.05589000 0.77531200 -1.63863600
C -3.93947000 0.77426600 -0.80309900
N -2.66201000 1.61972100 1.07144100
C -2.45412300 2.40964100 2.14760400
C -3.45900000 3.28093500 2.58068000
C -4.66673600 3.33288900 1.89484500
C -4.85494100 2.52264100 0.77936500
C -3.82562800 1.66890200 0.37766700
C -1.12676700 2.34095600 2.84349100
C -1.78997000 -1.90722900 -2.21222800
Br -1.30640100 -1.29884500 2.29694300
F 0.19870600 -0.42018700 -0.45051600
N 1.65360900 1.00847500 -1.59948000
S 1.27429100 2.32110500 -0.57493300
S 0.69225500 0.75675800 -3.03010500
H -4.03293400 -1.70582000 -3.71477000
H -5.88372300 1.45170000 -1.47219500
H -5.79073800 2.55681400 0.23830100
H -3.28133400 3.90635300 3.44767100
H -5.94730400 -0.13244900 -3.37724700
H -5.45741500 3.99994200 2.22216600
H -0.88894000 1.30656400 3.10433900
H -0.33976000 2.69820600 2.17469700
H -1.12989000 2.95110400 3.74802200
H -1.36661100 -1.80796100 -3.21539300
H -0.99417800 -1.72449200 -1.49460300
H -2.16062600 -2.93275600 -2.10537400
C 1.55256300 1.89044600 -4.10180100
O 1.00183500 -0.61546700 -3.43238300
O -0.69771800 1.19782900 -2.87741600
C 2.29264500 1.94849200 0.83997000
O 1.75321000 3.54453900 -1.23357700
O -0.15328000 2.30808200 -0.16075500
C 2.75697500 1.47911300 -4.68374900
C 3.43536300 2.37361600 -5.50894800
C 2.91143500 3.65031400 -5.73686500
C 1.01506400 3.16343700 -4.31562600
C 1.70520700 4.04302100 -5.14611700
H 3.13966700 0.48183100 -4.49871600
H 4.36912300 2.07529300 -5.97405100
H 3.44536500 4.34346000 -6.37950700
H 0.08103900 3.44648700 -3.84499500
H 1.30365400 5.03358400 -5.33270600
C 3.15891600 2.94799700 1.28833400
C 3.89752000 2.71175700 2.44866500
C 3.76407000 1.49939900 3.13011000
C 2.14605000 0.72410700 1.50108400
C 2.89617900 0.51030500 2.65588700
H 3.24968600 3.88068100 0.74352800
H 4.57567200 3.47481700 2.81657700
H 4.34141800 1.32256700 4.03235000
H 1.47812100 -0.03223200 1.10560600
H 2.79924100 -0.43227000 3.18471800

TS-F-Br’
E(BS1) = -2498.499306
E(BS2) = -2500.360945
ZPE = 0.424886
H = 0.460842
G = 0.356904
64.3497i
Cu -1.43454200 0.22939100 0.65119900
N -2.75337600 -0.23367800 -0.90723700
C -2.60596800 -1.23242500 -1.79365700
C -3.44212200 -1.30592100 -2.91460600

C -4.42717800 -0.34076200 -3.09310000
C -4.58594600 0.66727800 -2.14367500
C -3.72555100 0.69202700 -1.04457700
N -2.92951300 1.57566100 1.06144000
C -2.94904500 2.42789300 2.10480800
C -3.90519100 3.44832200 2.16946400
C -4.83071500 3.58393100 1.14120400
C -4.79443000 2.69875400 0.06662100
C -3.82594500 1.69336200 0.05102600
C -1.90567500 2.25242800 3.16884000
C -1.53054300 -2.24274300 -1.51944300
Br -0.83344900 -1.28400500 2.53133300
F 0.14352800 0.01762100 -0.33157400
N 1.19642500 1.21049400 -1.38645800
S 1.21417200 2.48787800 -0.22952800
S 0.08967700 1.35220900 -2.71883500
H -3.31207500 -2.11155500 -3.62821800
H -5.36470300 1.40860300 -2.26638700
H -5.50908300 2.79572700 -0.73982100
H -3.90939400 4.12262700 3.01812800
H -5.07928900 -0.37617500 -3.95985900
H -5.57695800 4.37116100 1.17248100
H -1.88268700 1.21456500 3.51024000
H -0.91947200 2.47609300 2.75114700
H -2.09146600 2.91442200 4.01653700
H -0.55551900 -1.76573500 -1.62916200
H -1.60976200 -2.59634400 -0.48657700
H -1.60126200 -3.09184100 -2.20155000
C 1.09552300 2.32491700 -3.82219000
O -0.01993500 -0.00716600 -3.25364500
O -1.11864900 2.10548800 -2.36037500
C 2.48829700 1.87993800 0.85118400
O 1.69159600 3.68106300 -0.94455300
O -0.05464000 2.57708600 0.51626000
C 2.10748500 1.68239400 -4.54375400
C 2.89093000 2.44267100 -5.40949200
C 2.65730100 3.81544700 -5.54186400
C 0.84929300 3.69543600 -3.93808900
C 1.63895400 4.43875000 -4.81270600
H 2.26482900 0.61552000 -4.43296900
H 3.67985400 1.96498700 -5.98112100
H 3.27155700 4.40270100 -6.21741200
H 0.06136500 4.15736600 -3.35592600
H 1.46103000 5.50328300 -4.92455600
C 3.80722500 2.27415200 0.61319300
C 4.80479700 1.79757400 1.46312000
C 4.47602500 0.94311800 2.52013000
C 2.13736500 1.02864100 1.90084200
C 3.14911000 0.56068000 2.73806700
H 4.03870700 2.94178200 -0.20900400
H 5.83584300 2.09450300 1.30071600
H 5.25792100 0.57466100 3.17707700
H 1.10863300 0.72933800 2.05700000
H 2.89668800 -0.10442800 3.55758200

3-F-Br’
E(BS1) = -2498.051197
E(BS2) = -2498.156823
ZPE = 0.427362
H = 0.462976
G = 0.360368

Cu -1.26406300 0.31990300 0.45594800
N -2.85661700 -0.03667800 -1.04807300
C -2.91312700 -0.91713200 -2.05775500
C -4.02526000 -0.93661400 -2.91724300
C -5.06716100 -0.04290400 -2.71542400
C -5.00295900 0.85311800 -1.64763100
C -3.87513900 0.82448100 -0.82817500
N -2.60568800 1.54240000 1.11015400
C -2.34451500 2.28837900 2.20465400
C -3.26295700 3.26820800 2.59009600
C -4.41679000 3.46464100 1.83946400
C -4.64616700 2.68468200 0.71105800
C -3.71737300 1.70684800 0.34889900
C -1.07264400 2.05051100 2.96253100
C -1.77262000 -1.87147900 -2.25145900
Br -2.09009700 -1.36880400 2.09240000
F 0.03868700 -0.74576900 -0.03826000
N 1.66516100 1.03721800 -1.53132600
S 1.14077900 2.19040000 -0.57328300
S 0.88441700 0.71819800 -2.96177800
H -4.05526300 -1.65476000 -3.72946700
H -5.81922100 1.54008400 -1.46575300
H -5.53514200 2.83752600 0.11425000
H -3.05607100 3.86361400 3.47120100
H -5.93122700 -0.04467300 -3.37172700
H -5.13486100 4.22467700 2.12829300
H -0.99780300 1.00159800 3.26229900
H -0.21319600 2.27970400 2.32763400
H -1.03339700 2.67797700 3.85318300
H -1.10381800 -1.49132000 -3.02849200
H -1.18217700 -1.96043600 -1.34170900
H -2.14541100 -2.85123200 -2.56274800
C 1.64326400 1.84753000 -4.13519100
O 1.28614700 -0.64536800 -3.34262700
O -0.55477900 1.04341800 -2.91122800
C 2.17448800 1.97193000 0.86708100
O 1.26594300 3.58155900 -1.05648800
O -0.31391100 2.00214600 -0.08488600
C 2.74394400 1.41956600 -4.87929800
C 3.34208300 2.31115200 -5.77149500
C 2.84093400 3.60853600 -5.90760600
C 1.12936100 3.13999000 -4.26095200
C 1.73671500 4.02121000 -5.15493500
H 3.11224800 0.40632900 -4.76411700
H 4.19660200 1.99152700 -6.35987800
H 3.30986600 4.29916200 -6.60207400
H 0.27692400 3.44545700 -3.66590300
H 1.34739100 5.02859800 -5.26532000
C 2.87050500 3.08146700 1.34753300
C 3.62936500 2.94094200 2.51140400
C 3.67773500 1.71152000 3.17231400
C 2.20869400 0.73161500 1.51294300
C 2.96984500 0.61205800 2.67455300
H 2.81754600 4.02650100 0.81924800
H 4.18094000 3.79187500 2.89820800
H 4.26836800 1.60827300 4.07749400
H 1.64186200 -0.10162100 1.11270700
H 3.00978700 -0.34177300 3.19116400

2-N-Br
E(BS1) = -2498.518503

E(BS2) = -2500.370899
ZPE = 0.425754
H = 0.462368
G = 0.357694
N 0.96951500 0.16720000 -0.31440600
S 2.64777600 -0.26560800 -0.99129800
S 0.39669100 1.88086300 -0.66061400
C 3.04034100 -1.62196100 0.07621000
O 3.57168600 0.84077100 -0.74221300
O 2.31912900 -0.70169100 -2.34472400
C 1.60646400 2.97421200 0.03759200
O -0.82943500 1.97890700 0.12277600
O 0.41367400 1.93749700 -2.12079100
C 2.63727600 -2.90459500 -0.29671400
C 3.72025200 -1.35491100 1.27086300
C 1.60294700 3.17601500 1.42153100
C 2.51086900 3.60965200 -0.81597400
C 2.92845900 -3.96009600 0.56740800
H 2.08950900 -3.06845400 -1.21666400
C 3.99892200 -2.42350200 2.11828500
H 4.01840600 -0.34344800 1.52041700
C 2.55192900 4.04153300 1.96166200
H 0.87612000 2.67679100 2.05055900
C 3.44270200 4.48113500 -0.25673600
H 2.48279300 3.42094500 -1.88220800
C 3.60129400 -3.71986600 1.76757400
H 2.62016900 -4.96563700 0.30169200
H 4.52484200 -2.24680000 3.05055000
C 3.46648300 4.68982800 1.12558300
H 2.57296600 4.21353600 3.03263300
H 4.15170800 4.99231700 -0.89926900
H 3.81937700 -4.54633000 2.43673600
H 4.20021600 5.36529400 1.55420800
F 1.16927500 0.21136300 1.10164600
Cu -0.67322900 -1.38571900 -0.79567600
C -1.79467200 -1.18881800 3.45858200
C -2.87018000 -0.30647800 3.41922600
C -3.34045900 0.14554500 2.18816700
C -2.71073400 -0.29780500 1.02089600
C -1.19618800 -1.58522900 2.25682000
C -3.16145500 0.11783900 -0.33966000
C -4.37642500 0.77471800 -0.55720600
C -4.71716200 1.14331100 -1.85699000
H -5.65413800 1.65651500 -2.04940200
C -3.85087900 0.83995400 -2.90292900
C -2.65771000 0.16257400 -2.62404400
H -1.41547700 -1.56754300 4.40158000
H -3.34328500 0.02603400 4.33787200
H -4.08971500 1.11399900 -3.92469600
N -1.65140600 -1.13352200 1.07488500
N -2.33538000 -0.18043900 -1.36433900
C -1.67209600 -0.21277600 -3.69374100
H -0.73601600 0.33131600 -3.53530900
H -1.44083600 -1.28054000 -3.62152200
H -2.05291500 0.01406800 -4.69198600
C -0.01440000 -2.51454300 2.21974700
H -0.07781600 -3.16733800 1.34466800
H 0.91081200 -1.93882700 2.12390500
H 0.05080000 -3.12033300 3.12649900
H -4.17082400 0.83883300 2.14695400
H -5.05354700 0.98584600 0.26071600
Br -0.47466900 -3.56875000 -1.94331800

TS-N-F-Br
E(BS1) = -2498.493445
E(BS2) = -2500.353687
ZPE = 0.424025
H = 0.460370
G = 0.356392
$\langle S^2 \rangle$ = 0.3785
858.2295i
N 0.54719600 -0.04778900 -0.62199800
S 1.98047700 -0.44886800 -1.65196900
S 0.27703000 1.62652000 -0.10968600
C 3.25335900 -0.98661400 -0.53747300
O 2.36032200 0.81265900 -2.29364800
O 1.51404000 -1.58038400 -2.44635000
C 1.81612400 2.22516600 0.54579900
O -0.71045400 1.52606500 0.96285500
O -0.04910100 2.30432100 -1.36597000
C 3.19270400 -2.29289700 -0.04127000
C 4.27727900 -0.09803900 -0.20788900
C 2.21697200 1.79940800 1.81624200
C 2.57837600 3.10286500 -0.22908800
C 4.20760200 -2.71525300 0.81308700
H 2.36607300 -2.94353400 -0.30771900
C 5.28254700 -0.54164500 0.65124800
H 4.28660000 0.90783300 -0.60916600
C 3.43012900 2.26900300 2.31558600
H 1.60304900 1.11049300 2.38168600
C 3.78559800 3.56704200 0.29262800
H 2.23470200 3.39727100 -1.21273500
C 5.24630800 -1.84259700 1.15904500
H 4.18586400 -3.72370100 1.21305300
H 6.08840700 0.13204700 0.92290200
C 4.20984100 3.14815500 1.55757600
H 3.76722500 1.94608600 3.29487200
H 4.39374100 4.25124900 -0.28962700
H 6.03096100 -2.18040500 1.82903200
H 5.15362800 3.50850800 1.95476100
F 0.95444000 -0.72847500 0.88890100
Cu -1.07718100 -1.39846700 -0.22799900
C -3.50242600 1.02105900 -2.99050200
C -4.53920500 1.44020700 -2.16647400
C -4.57644400 1.00850700 -0.84223600
C -3.56700500 0.16270100 -0.37659400
C -2.51225800 0.17805500 -2.47034500
C -3.52842200 -0.33579600 1.02759700
C -4.54648900 -0.06503900 1.94788800
C -4.41431300 -0.53329800 3.25237700
H -5.19215900 -0.33510000 3.98286500
C -3.27567000 -1.24859700 3.60960700
C -2.29557300 -1.49881500 2.64231700
H -3.44545700 1.33879400 -4.02568400
H -5.31466600 2.09753400 -2.54638700
H -3.13786400 -1.61234100 4.62175000
N -2.56018000 -0.23301400 -1.18981200
N -2.44377600 -1.05630800 1.38015300
C -1.03336700 -2.24920000 2.95591900
H -0.97089500 -3.15030000 2.33810500
H -0.16715300 -1.63479400 2.69382800
H -0.98174300 -2.52407000 4.01115600

C -1.34697200 -0.26103000 -3.30846200
H -0.52878500 0.45840100 -3.19640500
H -0.97337100 -1.23702900 -2.99417300
H -1.61807100 -0.30034100 -4.36607600
H -5.42717200 0.49521600 1.66296400
H -5.37721700 1.33484600 -0.19200100
Br -0.46884500 -3.78537500 -0.47097400

3-N-F-Br
E(BS1) = -2498.530511
E(BS2) = -2500.396956
ZPE = 0.425799
H = 0.462328
G = 0.353836
$<S^2>$ = 0.9584
N 0.60488900 -1.03819800 -0.45342200
S 1.86785100 -2.11719400 -0.10052400
S 0.66975900 0.00980000 -1.76154500
C 3.32167700 -1.13426700 0.22175200
O 2.12721300 -2.95490000 -1.28009600
O 1.44813000 -2.77042800 1.14753100
C 1.67837700 1.41162200 -1.27559000
O -0.71139200 0.48503300 -1.94435100
O 1.34213400 -0.63030100 -2.90293600
C 3.53930700 -0.66316500 1.51781300
C 4.16459300 -0.80964100 -0.84298800
C 1.80430700 1.77810600 0.06361600
C 2.28327200 2.14418000 -2.30120200
C 4.64475900 0.15419700 1.74877500
H 2.83394600 -0.90436200 2.30300100
C 5.26034400 0.01734700 -0.59513800
H 3.95379300 -1.18667900 -1.83692400
C 2.55991900 2.90913000 0.37712400
H 1.37058300 1.16785400 0.84607100
C 3.02301200 3.27918600 -1.97173800
H 2.18834100 1.82149100 -3.33205500
C 5.49890600 0.49620500 0.69584300
H 4.83331000 0.53202400 2.74868400
H 5.92397100 0.28826700 -1.40996000
C 3.16088400 3.66186700 -0.63412500
H 2.68952400 3.19036200 1.41751100
H 3.49935500 3.85669100 -2.75772100
H 6.35225200 1.14132800 0.88166200
H 3.74741400 4.53958300 -0.38022800
F 0.61800200 -0.23650200 2.16833200
Cu -0.71155000 -0.70387700 1.07636900
C -3.27805100 -2.85745200 -1.45384500
C -4.14664300 -1.87494500 -1.90574900
C -4.03116700 -0.58139500 -1.40800300
C -3.03977100 -0.28709100 -0.47271900
C -2.29075700 -2.53653800 -0.51541600
C -2.86639500 1.06387700 0.10644400
C -3.73822200 2.11271800 -0.18406200
C -3.52147600 3.35032000 0.42043400
H -4.18269900 4.18359400 0.20673100
C -2.46503500 3.49346500 1.30649500
C -1.62639600 2.39835900 1.57096300
H -3.34483700 -3.87826100 -1.81135300
H -4.91294100 -2.11042800 -2.63651700
H -2.27828100 4.43621400 1.80925000
N -2.19598900 -1.26994500 -0.06392700
N -1.82555100 1.21999600 0.95679000
C -0.50340600 2.53686600 2.56009100
H -0.03339500 1.57569100 2.75490700
H 0.25140900 3.22900000 2.17190200
H -0.88371400 2.96141700 3.49445500
C -1.33657000 -3.58768100 -0.03787900
H -0.49597500 -3.66122300 -0.73422200
H -0.93410200 -3.36477600 0.94900400
H -1.83790600 -4.55729300 -0.00667700
Br -2.14830100 -1.52001500 3.01310100
H -4.70253500 0.19215900 -1.75355600
H -4.57474500 1.98344700 -0.85745100

TS-N-F-Br’
E(BS1) = -2498.484198
E(BS2) = -2500.342233
ZPE = 0.424661
H = 0.460607
G = 0.358513
312.6440i
N 0.54448500 0.13882800 -0.85342000
S 2.04480500 -0.34172800 -1.73512800
S 0.36092800 1.78538400 -0.24278200
C 3.21062600 -0.95761500 -0.54758800
O 2.52658800 0.91423300 -2.31714400
O 1.58590400 -1.43523300 -2.58343900
C 1.87726800 2.28357900 0.53404700
O -0.70160000 1.68978300 0.75778400
O 0.15103000 2.53199700 -1.48590800
C 3.05891000 -2.26962200 -0.08631600
C 4.25024600 -0.12420500 -0.13265300
C 2.15719500 1.82095200 1.82385000
C 2.74078800 3.13042800 -0.16611500
C 3.99568700 -2.75568500 0.82151300
H 2.22108900 -2.87444800 -0.41801400
C 5.17557800 -0.63109300 0.77910700
H 4.33254400 0.88660500 -0.51165100
C 3.35146000 2.21880400 2.42116300
H 1.46596500 1.15992600 2.33012800
C 3.92747100 3.52178500 0.45286800
H 2.48907900 3.45711100 -1.16712500
C 5.04712200 -1.93886800 1.25414200
H 3.90250800 -3.76990700 1.19574200
H 5.99205400 -0.00169300 1.11703700
C 4.23170900 3.06454100 1.73896700
H 3.59481200 1.86668000 3.41796600
H 4.61307000 4.17998900 -0.07027500
H 5.77000500 -2.32553000 1.96592500
H 5.16006500 3.36895300 2.21197000
F 0.88168200 -0.62237100 0.78680200
Cu -0.93499200 -1.13809600 -0.19774500
C -3.70202600 0.77586200 -3.03874100
C -4.76883700 1.10990500 -2.21332200
C -4.72197900 0.76918600 -0.86297100
C -3.59411700 0.10695800 -0.37222100
C -2.59733700 0.10678500 -2.49553300
C -3.46091300 -0.30658300 1.05203400
C -4.41690500 0.00594200 2.02355200
C -4.20921700 -0.42370400 3.33174300
H -4.93708300 -0.19061900 4.10229400
C -3.06271500 -1.14938400 3.64078100

C -2.13905000 -1.43019600 2.62764000
H -3.71121100 1.02300300 -4.09455300
H -5.63583600 1.62470000 -2.61458000
H -2.87277200 -1.49236200 4.65172500
N -2.56221600 -0.20636200 -1.18801000
N -2.35391400 -1.01077900 1.36740900
C -0.86842000 -2.18898600 2.88319200
H -0.80889100 -3.05097300 2.21123200
H -0.00617000 -1.55674800 2.64963400
H -0.80276900 -2.52288000 3.92033200
C -1.40932500 -0.25938500 -3.33790500
H -0.64022400 0.51510100 -3.24856100
H -0.96492300 -1.19923600 -3.00441200
H -1.68939100 -0.34059100 -4.39054200
H -5.30319200 0.57555400 1.77645900
H -5.55594500 1.00900500 -0.21650800
Br -0.55028500 -3.59108000 -0.71922000

3-N-F-Br’
E(BS1) = -2498.514173
E(BS2) = -2500.387601
ZPE = 0.427362
H = 0.462976
G = 0.360368
N 0.58857700 -1.02550000 -0.43730600
S 1.85125500 -2.11140600 -0.07515300
S 0.65831400 0.01434400 -1.75679100
C 3.31041900 -1.13604500 0.24241700
O 2.10247800 -2.95159000 -1.25383300
O 1.42775700 -2.75673400 1.17476400
C 1.69138800 1.40237500 -1.28815000
O -0.71658500 0.50354500 -1.94009600
O 1.31817100 -0.65180300 -2.89002400
C 3.53401200 -0.66664900 1.53797600
C 4.15475200 -0.82079200 -0.82403100
C 1.83311700 1.77191700 0.04813500
C 2.30494000 2.11596100 -2.32193900
C 4.64811800 0.13933200 1.76697400
H 2.82803400 -0.90157800 2.32456900
C 5.25905100 -0.00488300 -0.57797000
H 3.93917200 -1.19664000 -1.81741100
C 2.61586200 2.88709500 0.35145300
H 1.38817000 1.17501200 0.83458400
C 3.07115600 3.23609200 -2.00254500
H 2.19673900 1.79018100 -3.35049800
C 5.50431200 0.47175300 0.71258300
H 4.84213200 0.51535700 2.76646600
H 5.92411600 0.25904100 -1.39384700
C 3.22662900 3.62129000 -0.66756800
H 2.75828600 3.17038600 1.38955600
H 3.55466600 3.79954400 -2.79428800
H 6.36456800 1.10799800 0.89695900
H 3.83446800 4.48666800 -0.42168000
F 0.60989200 -0.22114600 2.15973700
Cu -0.71450500 -0.71027200 1.08183000
C -3.22097800 -2.82798500 -1.50690900
C -4.07417100 -1.84103700 -1.97738800
C -3.98050900 -0.55581400 -1.45585600
C -3.02515100 -0.27018400 -0.48087000
C -2.26230300 -2.51748800 -0.53601100
C -2.89744500 1.07076200 0.13128200
C -3.85490700 2.06603100 -0.06906800
C -3.68096600 3.29615600 0.56350600
H -4.40872200 4.08853900 0.42280100
C -2.58080100 3.48242400 1.38712000
C -1.66127200 2.43557700 1.56353000
H -3.27455200 -3.84505400 -1.87696100
H -4.81117000 -2.06707300 -2.74053900
H -2.42461700 4.42014200 1.90936500
N -2.18335500 -1.25524200 -0.06813900
N -1.82174500 1.26462900 0.92580800
C -0.48167000 2.61224700 2.47806500
H -0.03010100 1.65113600 2.71601400
H 0.27321700 3.24173900 1.99443300
H -0.78986600 3.11941600 3.39690100
C -1.32818200 -3.57861500 -0.04122200
H -0.47683600 -3.66401400 -0.72275200
H -0.94264000 -3.36046500 0.95326300
H -1.84308500 -4.54144200 -0.01967700
Br -2.12029100 -1.47331200 2.99289800
H -4.63542800 0.22462300 -1.81747700
H -4.72848000 1.89628800 -0.68425800

4-F-Br
E(BS1) = -2498.528960
E(BS2) = -2500.396659
ZPE = 0.425441
H = 0.462058
G = 0.355642
<S^2> = 2.0088
Cu -1.33068600 0.39148600 0.55417100
N -2.72777300 -0.21970700 -0.90289800
C -2.62076500 -1.26235300 -1.74677400
C -3.47362500 -1.35786700 -2.85465000
C -4.43582900 -0.37791700 -3.06633600
C -4.56208700 0.66838900 -2.15472800
C -3.68830500 0.71445400 -1.06796200
N -2.81782400 1.70012700 0.96383000
C -2.82482100 2.58642600 1.98370200
C -3.81240600 3.57705900 2.04638600
C -4.78420400 3.64809400 1.05667600
C -4.76405500 2.72495900 0.01535600
C -3.76305400 1.75358100 -0.01018700
C -1.77201700 2.48445200 3.04775100
C -1.58650500 -2.30613400 -1.44900000
Br -1.21531900 -1.04004400 2.68816800
F 0.18744300 -0.36291900 -0.20391900
N 1.42694700 1.18526500 -1.47705800
S 1.32944900 2.34297000 -0.21352100
S 0.20462600 1.22200900 -2.71455900
H -3.37158800 -2.19622600 -3.53426300
H -5.32311400 1.42379000 -2.29926200
H -5.51915400 2.76273800 -0.75798500
H -3.80390500 4.28020500 2.87097300
H -5.09577400 -0.43154500 -3.92612000
H -5.55394400 4.41193500 1.09354800
H -1.89532200 1.55407200 3.60793400
H -0.77607700 2.46057300 2.60219800
H -1.83818200 3.33093800 3.73350300
H -0.59490300 -1.85426600 -1.48331000
H -1.72929200 -2.68507300 -0.43160300
H -1.65321600 -3.13461700 -2.15615300

C 1.05803800 2.27878200 -3.86629800
O 0.16029700 -0.14109300 -3.24520800
O -1.02705900 1.88580400 -2.26840800
C 2.58421300 1.74298200 0.89674500
O 1.74264500 3.62840400 -0.79696100
O 0.01460900 2.30172100 0.46875600
C 1.95639700 1.70079000 -4.76996000
C 2.62281200 2.53320100 -5.66692000
C 2.39118800 3.91251900 -5.64630600
C 0.81637800 3.65605100 -3.82754800
C 1.49116400 4.47183900 -4.73229200
H 2.11386300 0.62824500 -4.77200000
H 3.31991400 2.10683000 -6.38066900
H 2.91509200 4.55589400 -6.34635000
H 0.12099800 4.06796400 -3.10638700
H 1.31575800 5.54253300 -4.72508300
C 3.71611700 2.53799200 1.09011100
C 4.67624500 2.10939700 2.00734500
C 4.49418600 0.91146000 2.70337900
C 2.38015300 0.53816000 1.57811500
C 3.35485200 0.12945900 2.48663600
H 3.83436400 3.46473000 0.54070500
H 5.56253300 2.71203600 2.17713300
H 5.24487400 0.58401900 3.41615900
H 1.50487200 -0.06577400 1.37008700
H 3.22092500 -0.80283700 3.02572900

5-N-F-Br
E(BS1) = -2498.539043
E(BS2) = -2500.408980
ZPE = 0.427426
H = 0.463237
G = 0.359776
$<S^2>$ = 2.0055
N -0.69019500 -0.91039300 -0.61484500
S -1.93821400 -1.23467800 -1.68053000
S -0.72746800 -1.33863500 0.98777100
C -3.36672600 -0.33600100 -1.09284600
O -2.27037200 -2.66839400 -1.68310300
O -1.49131400 -0.61465300 -2.94174200
C -1.74080500 -0.11925600 1.83211800
O 0.66209200 -1.13485900 1.44828700
O -1.34907000 -2.65577100 1.20058500
C -3.40320600 1.04788700 -1.28164600
C -4.38134700 -1.02388800 -0.42678700
C -1.67746000 1.22549400 1.45669800
C -2.50968900 -0.54484900 2.91727900
C -4.50358200 1.75358900 -0.79841900
H -2.55881500 1.54834000 -1.74210900
C -5.47232600 -0.30003700 0.05755600
H -4.31076900 -2.09729200 -0.29407100
C -2.39860000 2.15916000 2.20130800
H -1.13503900 1.52891100 0.56685200
C -3.22475500 0.40246800 3.65111300
H -2.55113000 -1.59806800 3.17102300
C -5.53417600 1.08284800 -0.13175700
H -4.55063600 2.83023200 -0.92942200
H -6.27101200 -0.81641400 0.58067400
C -3.16461900 1.75281600 3.29741700
H -2.37398000 3.20521000 1.91136500
H -3.83027200 0.08423100 4.49406300
H -6.38481400 1.64120200 0.24750200
H -3.72478900 2.48703800 3.86864800
F -0.38613000 1.80006700 -1.37865200
Cu 0.76471000 0.40757300 -1.19382500
C 3.35799700 -2.97988900 -1.76249000
C 4.33712500 -2.75791500 -0.80446900
C 4.26112100 -1.61768600 -0.00524100
C 3.19370100 -0.74422200 -0.18917400
C 2.30886100 -2.06244200 -1.91554000
C 3.04118100 0.48650100 0.61578400
C 3.55628600 0.61126100 1.90398300
C 3.33182200 1.79580900 2.60375600
H 3.71709500 1.91203100 3.61130200
C 2.60962500 2.82235800 2.00642100
C 2.11674500 2.66311600 0.70420400
H 3.39270000 -3.85289100 -2.40405800
H 5.16004300 -3.45465000 -0.68496000
H 2.42583000 3.75496500 2.52729000
N 2.24612300 -0.97094500 -1.13023900
N 2.32167100 1.49225100 0.06593300
C 1.39269100 3.76050100 -0.01224700
H 2.08473000 4.26554200 -0.69504900
H 0.58152100 3.33456600 -0.60693900
H 1.00982700 4.49547600 0.69841100
C 1.23016600 -2.28865200 -2.93110300
H 0.40543100 -2.84772500 -2.47755800
H 0.81425000 -1.35106800 -3.30097400
H 1.61330000 -2.86840200 -3.77351300
H 4.08203700 -0.21545300 2.36438100
H 5.02947800 -1.40165000 0.72612200
Br 2.83776700 1.89711700 -2.61544300

D1-N-F-2Br
E(BS1) = -3282.129022
E(BS2) = -3285.543477
ZPE = 0.645396
H = 0.698531
G = 0.557690
$<S^2>$ = 2.0059
N -0.65788600 0.22818900 -1.19454700
S -0.50329100 1.22207000 0.15382600
S 0.21775200 0.54988400 -2.58295500
C -1.31452200 2.74383500 -0.33997500
O 0.89215800 1.55223300 0.47939600
O -1.31786000 0.58281800 1.19750700
C -0.71058000 1.80247500 -3.46430400
O 0.08908100 -0.72120800 -3.33359500
O 1.56418000 1.08730200 -2.34177600
C -2.58873000 2.68776900 -0.91109800
C -0.66918900 3.95841600 -0.10881600
C -1.91245300 1.44432500 -4.07647500
C -0.23975800 3.11526600 -3.48442800
C -3.22373800 3.87685200 -1.25915300
H -3.05503800 1.73104900 -1.10822300
C -1.31944600 5.14448800 -0.45613900
H 0.32529300 3.96589800 0.32213800
C -2.65986600 2.42629500 -4.72263200
H -2.25114200 0.41841900 -4.05167500
C -0.99970400 4.09085800 -4.13125600
H 0.69522900 3.36263700 -2.99611800
C -2.59168500 5.10362200 -1.02948100

H -4.20554300 3.84630500 -1.72065200
H -0.82734500 6.09682400 -0.28548100
C -2.20597700 3.74860000 -4.74690500
H -3.59526500 2.15765800 -5.20370400
H -0.65051000 5.11814800 -4.14744900
H -3.08987400 6.02748800 -1.30719700
H -2.79400700 4.51323900 -5.24525900
Cu -0.99053300 -1.81214900 -1.13587700
C -3.95474500 -1.55844900 -4.44230100
C -4.92468500 -0.75152100 -3.86260400
C -4.84209600 -0.46766000 -2.50112000
C -3.76108100 -0.96388500 -1.76838400
C -2.87370500 -2.00699300 -3.66791800
C -3.71567100 -0.88320000 -0.28196300
C -4.60654000 -0.10031300 0.45736200
C -4.59921500 -0.21682500 1.84580200
H -5.28558200 0.37278500 2.44525300
C -3.71644000 -1.10398800 2.44864300
C -2.81413600 -1.81555700 1.64773600
H -4.01480500 -1.84745200 -5.48556400
H -5.75814600 -0.37897900 -4.44926200
H -3.70224600 -1.23585400 3.52502600
N -2.76725800 -1.65904400 -2.37036800
N -2.81804000 -1.69195100 0.31273300
C -1.77955800 -2.72626000 2.23487100
H -1.65755300 -3.60855800 1.60372000
H -0.81668700 -2.20590400 2.26154800
H -2.03885800 -3.03653300 3.24967100
C -1.82091100 -2.89088300 -4.26384000
H -1.01264900 -2.28549400 -4.68022900
H -1.39968400 -3.52803900 -3.48857700
H -2.24632000 -3.50373700 -5.06219000
H -5.29713300 0.57970100 -0.02543800
H -5.63188000 0.08946100 -2.01481700
F -0.78284500 -3.71420900 -1.44181000
Cu 0.73333600 -4.63160700 -0.66657500
C 3.79241300 -4.50997300 -4.03215000
C 4.88037800 -5.12084000 -3.41810900
C 4.76219700 -5.56401200 -2.10304300
C 3.54410100 -5.37945600 -1.44130400
C 2.59961800 -4.35828000 -3.31423400
C 3.33257800 -5.78789700 -0.02896400
C 4.30216400 -6.46552700 0.71494000
C 4.04129800 -6.78339000 2.04406400
H 4.78357300 -7.31188200 2.63311300
C 2.82619700 -6.41145300 2.60870100
C 1.88344400 -5.74229000 1.82428800
H 3.85520300 -4.14892200 -5.05258200
H 5.81605500 -5.24773800 -3.95301600
H 2.59702600 -6.63228800 3.64466900
N 2.49409200 -4.79043700 -2.04673200
N 2.14692300 -5.45743500 0.53277700
C 0.56562200 -5.28834400 2.37214800
H 0.51280200 -4.19794500 2.31559000
H -0.25363800 -5.69566500 1.77096500
H 0.43464300 -5.59767100 3.41008600
C 1.38018900 -3.72250600 -3.90582700
H 0.99843000 -2.94255800 -3.24426700
H 1.58539500 -3.28542900 -4.88492400
H 0.59214200 -4.47337600 -4.01630200
H 5.24648700 -6.74512700 0.26784200
H 5.60705500 -6.02909200 -1.61237800
Br 1.27831800 -2.16583700 0.13326400
Br -0.41413000 -6.89629700 -0.93763400

6-N-F
E(BS1) = -2485.389452
E(BS2) = -2487.051767
ZPE = 0.426675
H = 0.460161
G = 0.364525
<S^2> = 0.7523
N -0.17342700 -0.23594400 -2.07687500
S 1.18078800 -0.27435500 -1.15218800
S -0.26124800 0.65818700 -3.48567500
C 1.24104400 1.24964200 -0.22536400
O 2.45718700 -0.44900200 -1.85192300
O 0.75836100 -1.38168400 -0.21624500
C -1.81908700 1.52256200 -3.30675600
O -0.39227000 -0.25381300 -4.63353000
O 0.82272800 1.65668800 -3.48424500
C 0.05380400 1.76499000 0.30212900
C 2.47217400 1.86902400 -0.01357000
C -2.90363300 1.14775400 -4.09792300
C -1.89444400 2.59692900 -2.41516700
C 0.10656500 2.93089100 1.06265100
H -0.89168000 1.27876100 0.09700200
C 2.51031800 3.03647200 0.75160500
H 3.37228800 1.44878600 -0.44702600
C -4.09906400 1.86031400 -3.98136300
H -2.81016100 0.31351600 -4.78335600
C -3.09597400 3.29214900 -2.30055200
H -1.02679900 2.88899000 -1.83558600
C 1.33368200 3.56372100 1.28903200
H -0.80919300 3.34493600 1.47270100
H 3.45942900 3.53378500 0.92313600
C -4.19638600 2.92473200 -3.08319800
H -4.95212900 1.57735900 -4.58952800
H -3.17161100 4.12414900 -1.60778800
H 1.37061600 4.47265900 1.88135400
H -5.12922200 3.47284000 -2.99366200
Cu -1.11380000 -1.88028700 -1.28090900
C -3.60817100 -2.79683700 -4.64835300
C -4.80962200 -2.16413900 -4.33987300
C -4.92180300 -1.43511700 -3.15830500
C -3.81047100 -1.33802400 -2.31660900
C -2.53702000 -2.69898300 -3.75461700
C -3.82682100 -0.55581000 -1.04862400
C -4.81188000 0.39746100 -0.77716400
C -4.76549700 1.07423100 0.44027000
H -5.50631100 1.83353400 0.66911200
C -3.76481400 0.76564900 1.35542200
C -2.80613400 -0.20140200 1.02001100
H -3.49518000 -3.37368300 -5.55896800
H -5.65874800 -2.24397600 -5.01085000
H -3.71456000 1.26228300 2.31828900
N -2.66181800 -1.97071300 -2.62935100
N -2.83361400 -0.81643900 -0.17402000
C -1.73125400 -0.62059500 1.98562300
H -1.50259800 0.17532900 2.69818300
H -2.07039800 -1.49555100 2.55258300
H -0.82074800 -0.90237600 1.45391100

C -1.21818600 -3.38113700 -3.96853000
H -0.43404400 -2.62241500 -4.06176800
H -0.99375400 -3.99776300 -3.09193700
H -1.22592000 -3.99939900 -4.86758400
H -5.57574700 0.63645500 -1.50486800
H -5.85771200 -0.95928700 -2.89626100
F -1.20666800 -3.60611600 -0.75849900

7-2Br
E(BS1) = -796.700807
E(BS2) = -798.466122
ZPE = 0.217203
H = 0.236210
G = 0.169346
$<S^2>$ = 0.7531
Cu -1.68457100 -0.00703400 1.02822600
N -2.60558700 -0.17792600 -0.81493700
C -2.34046700 -1.11407500 -1.74761800
C -3.11935600 -1.19222300 -2.90880300
C -4.17812500 -0.30934800 -3.08488800
C -4.45591800 0.63363000 -2.09785400
C -3.64667500 0.67779400 -0.96238000
N -3.06104100 1.49199600 1.22860300
C -3.12910600 2.34571600 2.26940800
C -4.07762500 3.37534200 2.27081600
C -4.93449200 3.51971900 1.18590100
C -4.82476400 2.64937200 0.10358400
C -3.86738200 1.63569700 0.14923700
C -2.15722400 2.16487300 3.39869400
C -1.20351600 -2.06098800 -1.49641600
H -2.89078700 -1.94611500 -3.65315700
H -5.29457100 1.30741100 -2.21293700
H -5.46499200 2.77383900 -0.75946500
H -4.12548900 4.05302000 3.11521200
H -4.79333200 -0.35915500 -3.97713000
H -5.67393100 4.31353000 1.17161100
H -2.39158000 1.25619400 3.95988300
H -1.14059400 2.04761000 3.01034400
H -2.18262600 3.02107600 4.07473500
H -0.25030500 -1.52654200 -1.52892200
H -1.28770900 -2.50074200 -0.49794300
H -1.19254400 -2.86201600 -2.23717100
Br -1.99624100 -1.45105300 3.01683600
Br 0.75535100 0.05502200 0.70008400

NBrSI
E(BS1) = -1628.343076
E(BS2) = -1628.837988
ZPE = 0.207222
H = 0.226679
G = 0.160255
S -1.32152100 1.51883200 -0.08774500
O -1.65485100 2.37634900 -1.22203300
O -1.07240900 2.08031200 1.23597800
N 0.24061400 0.76402800 -0.53982000
S 0.80155300 -0.64136900 0.39884600
O 0.33511800 -1.88744200 -0.21056100
O 0.44243500 -0.31415100 1.77961000
C 2.55282100 -0.46484800 0.15253000
C 3.22557500 -1.41089600 -0.62189900
C 3.20298700 0.61286500 0.76080900
C 4.60350100 -1.26818000 -0.78836200
H 2.68147800 -2.23071700 -1.07606900
C 4.57810400 0.74031400 0.57976900
H 2.64504200 1.32912200 1.35373300
C 5.27394000 -0.19728300 -0.19178600
H 5.14980100 -1.99189600 -1.38410700
H 5.10622400 1.56888100 1.03982100
H 6.34557300 -0.09079600 -0.32819300
C -2.48348600 0.17727000 0.01316800
C -2.68982300 -0.44751100 1.24595200
C -3.12143500 -0.23984000 -1.15891000
C -3.57026900 -1.52710300 1.29844300
H -2.16547800 -0.10023300 2.12738100
C -3.99584000 -1.32284900 -1.08452700
H -2.94387400 0.27664500 -2.09491300
C -4.21691300 -1.96353900 0.13848700
H -3.74984900 -2.02616200 2.24494300
H -4.50504600 -1.66364600 -1.97993300
H -4.89882000 -2.80670500 0.18788500
Br 0.44938800 0.45099700 -2.47972700

D2-N-2F-Br
E(BS1) = -3368.760947
E(BS2) = -3372.010635
ZPE = 0.646513
H = 0.698847
G = 0.561155
$<S^2>$ = 2.0051
N -0.56670300 0.16683000 -1.35953700
S -0.31800200 1.18189600 -0.04406700
S 0.23733300 0.43979500 -2.80061900
C -1.22759900 2.67504200 -0.45765500
O 1.09008900 1.56945700 0.13326900
O -0.99926100 0.52736300 1.08292100
C -0.67723200 1.74341800 -3.61753700
O -0.00328800 -0.82077400 -3.53968700
O 1.62289500 0.90286300 -2.64605700
C -2.49999500 2.58274200 -1.02638100
C -0.65362700 3.90951700 -0.15170400
C -1.89476700 1.43646600 -4.22627900
C -0.18809100 3.04936800 -3.56969300
C -3.20692100 3.75226300 -1.29413700
H -2.90821700 1.61724300 -1.29228100
C -1.37491500 5.07490100 -0.41811000
H 0.34283400 3.95003800 0.27304100
C -2.64044700 2.46539700 -4.79799800
H -2.24666100 0.41421900 -4.25484300
C -0.94660500 4.07146300 -4.14130100
H 0.75930600 3.25468000 -3.08531700
C -2.64796500 4.99680800 -0.98657600
H -4.18751800 3.69206700 -1.75548000
H -0.93780100 6.04146400 -0.18786300
C -2.16976900 3.78107600 -4.75083100
H -3.58875900 2.23860200 -5.27538800
H -0.58427100 5.09358100 -4.10314600
H -3.20197500 5.90572200 -1.20073200
H -2.75769100 4.58152400 -5.18950400
Cu -0.97547600 -1.85408200 -1.22148100
C -4.05038700 -1.51209500 -4.42738400
C -4.96090800 -0.65658600 -3.82088100
C -4.82243900 -0.37152200 -2.46357300

C -3.74160200 -0.91479000 -1.76471500
C -2.96752900 -2.00852700 -3.68490900
C -3.63277700 -0.82868900 -0.28008300
C -4.48367600 -0.04030200 0.49907900
C -4.40864000 -0.15397500 1.88664500
H -5.06405700 0.43900900 2.51668700
C -3.50043900 -1.04198900 2.45024200
C -2.64037100 -1.75926400 1.60862700
H -4.15959000 -1.80333100 -5.46622600
H -5.79351800 -0.24624500 -4.38305000
H -3.43498800 -1.17014700 3.52527200
N -2.80178600 -1.65635000 -2.39624600
N -2.71229100 -1.63747500 0.27662400
C -1.57683000 -2.67059000 2.14242900
H -1.41003600 -3.49317800 1.44439500
H -0.63494900 -2.11850600 2.22222900
H -1.83924500 -3.06783500 3.12603300
C -1.98385400 -2.95798500 -4.30217000
H -1.18848300 -2.40542700 -4.80736000
H -1.53252900 -3.57147900 -3.52420400
H -2.48470500 -3.59532300 -5.03555600
H -5.19437900 0.64102200 0.04867400
H -5.56795300 0.22686400 -1.95647600
F -0.92464100 -3.75727500 -1.43377000
Cu 0.56924500 -4.72866800 -0.60774300
C 3.77360000 -4.09609300 -3.78987400
C 4.91964600 -4.38308700 -3.05622100
C 4.79470000 -4.89861700 -1.76734000
C 3.51139100 -5.11554300 -1.25695300
C 2.51810000 -4.31768500 -3.20842600
C 3.28031400 -5.66464500 0.10579400
C 4.28817600 -6.30537600 0.83195200
C 3.99929900 -6.79673400 2.10158300
H 4.76763900 -7.30043800 2.67918000
C 2.71574100 -6.64470300 2.61515900
C 1.73881400 -6.00725600 1.84367300
H 3.83796500 -3.69256800 -4.79439000
H 5.90348100 -4.20287000 -3.47759200
H 2.45839100 -7.01808000 3.59980100
N 2.40691900 -4.82197000 -1.96948400
N 2.03674700 -5.53109700 0.61820600
C 0.33578900 -5.80803000 2.33602000
H 0.16447000 -4.74406500 2.52587600
H -0.36181800 -6.13243500 1.55840400
H 0.15467900 -6.35849600 3.26080600
C 1.23972500 -3.96330700 -3.90503600
H 0.87251800 -3.00400700 -3.52798600
H 1.37775300 -3.87429100 -4.98503000
H 0.47467600 -4.71355100 -3.69501100
H 5.27426800 -6.43933400 0.40675600
H 5.67742700 -5.10365200 -1.17507100
Br 1.32343400 -2.25665600 -0.04476600
F -0.37170500 -6.30091700 -0.61277800

8-F-Br
E(BS1) = -883.338351
E(BS2) = -884.936333
ZPE = 0.218841
H = 0.236965
G = 0.174089
$<S^2>$ = 0.7532
Cu -1.67089300 0.04874300 1.12189100
N -2.56623100 -0.14709400 -0.81122400
C -2.27872800 -1.05943000 -1.75688100
C -3.10230300 -1.19271100 -2.88629000
C -4.22785500 -0.39177000 -3.01367700
C -4.51867400 0.54273700 -2.01929400
C -3.65970600 0.64722100 -0.92677100
N -3.01548700 1.54053300 1.21814500
C -3.06983300 2.42008500 2.23984200
C -4.04504100 3.42140900 2.25341400
C -4.94823700 3.50944800 1.19904300
C -4.85724400 2.61161700 0.13820600
C -3.86847800 1.62690500 0.16957300
C -2.04478400 2.28434800 3.32749900
C -1.07908400 -1.94535000 -1.57078100
F -0.01524200 -0.60302400 0.95575400
H -2.84897100 -1.92749700 -3.64252900
H -5.40079900 1.16411000 -2.10096000
H -5.53620300 2.69271900 -0.70007500
H -4.08005700 4.12051000 3.08072000
H -4.87990000 -0.48886000 -3.87552200
H -5.71088600 4.28117200 1.19285200
H -2.15519000 1.31769700 3.82767100
H -1.03574700 2.31764400 2.90183700
H -2.13980500 3.08570900 4.06173900
H -0.43375400 -1.56811100 -0.77645000
H -1.41147300 -2.95448900 -1.29919700
H -0.51988600 -2.02978900 -2.50725300
Br -2.54322500 -1.38592800 3.03590800

D3-N-3F
E(BS1) = -3455.423174
E(BS2) = -3458.498958
ZPE = 0.648245
H = 0.699454
G = 0.566775
$<S^2>$ = 2.0039
N -0.24440900 -0.40214900 -1.46228100
S 0.42348200 0.29803800 -0.08688500
S 0.32362700 0.05435600 -2.96050500
C 0.06930200 2.04707900 -0.26804100
O 1.88349600 0.14259600 0.00614200
O -0.39750100 -0.19291500 1.03082300
C -0.71169800 1.43169200 -3.47490600
O 0.00251400 -1.08306000 -3.84675100
O 1.70670000 0.55842900 -2.93035000
C -1.26667700 2.45823700 -0.25955900
C 1.11701100 2.95683400 -0.39544300
C -2.05395500 1.19731200 -3.77813800
C -0.16170100 2.71045100 -3.55100100
C -1.55270700 3.81514500 -0.38627700
H -2.05991900 1.72547300 -0.16119300
C 0.81712500 4.31615100 -0.51604900
H 2.13905200 2.59678200 -0.40816100
C -2.86498500 2.27011900 -4.14223600
H -2.44964200 0.19318300 -3.74382000
C -0.98293700 3.77862400 -3.91568500
H 0.88269000 2.86310900 -3.31054100
C -0.51240200 4.74259500 -0.51277900
H -2.58514000 4.15039800 -0.39014000
H 1.62179300 5.03781900 -0.61680300

C -2.33142200 3.56170800 -4.20571600
H -3.91014600 2.09537800 -4.37875000
H -0.56760400 4.78016100 -3.96383400
H -0.74111700 5.79931600 -0.61286200
H -2.96675900 4.39693500 -4.48468800
Cu -1.05670900 -2.34416800 -1.27760900
C -4.11176000 -1.90085900 -4.34391200
C -5.00413900 -1.03876600 -3.71851300
C -4.79682400 -0.70131200 -2.38332500
C -3.67250300 -1.20197500 -1.72533400
C -2.99397100 -2.37684500 -3.64474800
C -3.47546700 -1.01909600 -0.26387400
C -4.13073200 -0.03285800 0.47822300
C -3.97674300 -0.04352800 1.86447800
H -4.47366800 0.70592600 2.47237900
C -3.18626300 -1.02187100 2.45831300
C -2.51653600 -1.94598000 1.64578100
H -4.26873000 -2.22018900 -5.36766600
H -5.87035100 -0.65980800 -4.25097200
H -3.06203700 -1.06105900 3.53485700
N -2.76754000 -1.97924500 -2.37280100
N -2.66843500 -1.92791900 0.31320900
C -1.58948600 -2.99068700 2.19284000
H -1.98181700 -3.98898500 1.96874700
H -0.62570900 -2.89454100 1.68746900
H -1.46109500 -2.89549700 3.27301200
C -2.04009000 -3.34338600 -4.27619600
H -1.10786000 -2.83119300 -4.51684000
H -1.80432300 -4.13693600 -3.56564800
H -2.46578100 -3.76652200 -5.18788400
H -4.73793800 0.72398600 -0.00343200
H -5.51831000 -0.09400600 -1.85245600
F 0.38305500 -6.53308500 -0.60607200
F -1.03189100 -4.29254500 -1.56726500
Cu 0.55760600 -4.72867600 -0.46645700
C 3.25660400 -2.35422400 -3.15045000
C 4.10561600 -1.83378000 -2.17993300
C 4.12148100 -2.40106100 -0.90739800
C 3.27111400 -3.47945100 -0.65222200
C 2.42979800 -3.43596800 -2.82289700
C 3.19390200 -4.12871000 0.68548600
C 4.25919800 -4.09235000 1.58902200
C 4.12517200 -4.73691700 2.81615000
H 4.94208500 -4.72515400 3.53069100
C 2.94224700 -5.40796000 3.10933500
C 1.90857200 -5.42102600 2.16643000
H 3.20399300 -1.91493600 -4.13944300
H 4.73074100 -0.97516300 -2.40172900
H 2.81026200 -5.92370200 4.05386600
N 2.44874200 -3.97565200 -1.59473700
N 2.05142000 -4.78489600 0.98741000
C 0.60184400 -6.11766500 2.41976000
H -0.17675200 -5.37740200 2.63366000
H 0.30472700 -6.66082400 1.51844700
H 0.67246600 -6.79614700 3.27215600
C 1.47736100 -4.05160300 -3.80870300
H 0.53741000 -4.28713100 -3.30548100
H 1.27809100 -3.37375700 -4.63976400
H 1.89097300 -4.98630700 -4.20530800
H 5.18342800 -3.59278800 1.32680000
H 4.74441100 -1.98319400 -0.12619800
F 0.41305000 -2.79818400 -0.14355100

9-2F
E(BS1) = -969.970368
E(BS2) = -971.406384
ZPE = 0.220181
H = 0.237393
G = 0.178570
$\langle S^2 \rangle$ = 0.7527
Cu -1.61797100 -0.02462400 1.04575900
N -2.61888200 -0.21187400 -0.79202600
C -2.35824100 -1.13870600 -1.73757800
C -3.09112600 -1.15691300 -2.93220300
C -4.10388100 -0.22788800 -3.13089200
C -4.38207300 0.70272700 -2.13206700
C -3.61932900 0.68559700 -0.96351000
N -3.02348400 1.51882000 1.22327000
C -3.13104900 2.35994600 2.27315500
C -4.13256200 3.33986000 2.29481300
C -5.00386700 3.45503500 1.21943400
C -4.85951000 2.60015300 0.12891900
C -3.85097100 1.63572000 0.15737300
C -2.12831500 2.24237500 3.38445700
C -1.30082800 -2.16709200 -1.45676800
F 0.07357400 -0.49201800 0.54175800
H -2.85983900 -1.90136700 -3.68562100
H -5.17727500 1.42264700 -2.27185300
H -5.52502600 2.68994300 -0.71903000
H -4.21151400 3.99972100 3.15135500
H -4.67970000 -0.22717300 -4.05065700
H -5.78673900 4.20651500 1.22162000
H -1.86236700 1.19312200 3.53618800
H -1.21448000 2.78056700 3.10201400
H -2.51067300 2.69014600 4.30417800
H -0.50038400 -1.72762400 -0.85639600
H -1.74268700 -2.98618400 -0.87502400
H -0.91189300 -2.58911900 -2.38604000
F -1.55044200 -0.58570300 2.78390900

DTSF-N-3F (Conf. A, Triplet)
E(BS1) = -5169.53189
E(BS2) = -5169.462064
ZPE = 0.857874
H = 0.927700
G = 0.752039
$\langle S^2 \rangle$ = 2.5975
266.6278i
N -0.36742700 -0.86747100 -1.09259200
S 0.40499700 0.05453800 0.05250500
S -0.16336100 -0.51908700 -2.68661700
C -0.09552200 1.74769600 -0.28431500
O 1.87636100 0.01301700 -0.03668800
O -0.21287100 -0.32150400 1.33888800
C -1.43786800 0.69422900 -3.07072000
O -0.53918500 -1.75349500 -3.40884100
O 1.13359700 0.10600700 -3.01440400
C -1.42032300 2.11430600 -0.03249600
C 0.82504400 2.65086100 -0.81288500
C -2.76922800 0.38011700 -2.78928100
C -1.08734200 1.91278400 -3.64680000
C -1.82576100 3.41630000 -0.31725500

H -2.11518100 1.38633400 0.37227300
C 0.40956100 3.95573800 -1.08737200
H 1.83957300 2.32480100 -1.00838800
C -3.76549800 1.30601600 -3.08765100
H -3.01550900 -0.56838000 -2.33378900
C -2.09303200 2.83759900 -3.93944000
H -0.04475200 2.13398500 -3.84052700
C -0.91149300 4.33596000 -0.84205900
H -2.85393100 3.71331700 -0.13549000
H 1.11597100 4.67087400 -1.49774800
C -3.42754000 2.53672800 -3.66181500
H -4.80247400 1.06856600 -2.86911800
H -1.82933400 3.79552700 -4.37701300
H -1.23291400 5.34921900 -1.06401500
H -4.20472900 3.26061700 -3.88816200
Cu -0.85233500 -3.05990400 -0.49546300
C -4.50099900 -2.95264800 -2.87165700
C -5.31987300 -2.22714200 -2.02100300
C -4.85872600 -1.91310100 -0.74398400
C -3.57656200 -2.31214000 -0.36969100
C -3.21421100 -3.33360900 -2.45835400
C -3.05521800 -2.10269600 1.00355900
C -3.74856700 -1.38055400 1.97370300
C -3.23978600 -1.35229600 3.27123300
H -3.76117000 -0.80273900 4.04837200
C -2.07496900 -2.04830900 3.55927100
C -1.38869600 -2.71865600 2.53530800
H -4.83723600 -3.23879500 -3.86185600
H -6.31318000 -1.92257900 -2.33379500
H -1.67212100 -2.06766900 4.56568800
N -2.76458400 -2.98436600 -1.23298700
N -1.88462000 -2.71917900 1.28388000
C -0.09908900 -3.42846000 2.81650400
H 0.73161400 -2.75665100 2.58475000
H -0.04899500 -3.70872300 3.87193200
H 0.01941200 -4.30394300 2.17644500
C -2.35609500 -4.13739900 -3.38886100
H -1.94556100 -3.47937900 -4.15854900
H -1.52411300 -4.60032800 -2.86575800
H -2.96565100 -4.90326700 -3.87655300
H -4.66583200 -0.85762900 1.73777000
H -5.49931100 -1.38461800 -0.05123600
F 0.35002300 -5.71195600 0.66097200
F -0.05471100 -4.48232700 -1.61673200
Cu 1.51533500 -4.80401700 -0.50449700
C 3.26899900 -2.29501000 -3.56147700
C 4.10843700 -1.51931200 -2.77543400
C 4.29365100 -1.84920300 -1.43345900
C 3.59420100 -2.93474500 -0.91509700
C 2.57474300 -3.37257200 -2.99124600
C 3.81375800 -3.48407600 0.44180500
C 4.70536000 -2.93548100 1.36133400
C 4.93948500 -3.61691700 2.55462000
H 5.62432100 -3.20545400 3.28900400
C 4.31395300 -4.83631000 2.78299000
C 3.41149900 -5.34661300 1.83781800
H 3.12788400 -2.07653100 -4.61351400
H 4.63070300 -0.66987900 -3.20268400
H 4.50884100 -5.40524300 3.68483100
N 2.73239600 -3.63381900 -1.68557000
N 3.16078700 -4.63790000 0.72210500
C 2.74811400 -6.67707100 2.00440500
H 1.67630300 -6.57300900 1.82839800
H 3.12432400 -7.35877800 1.23559600
H 2.94905000 -7.09044100 2.99444200
C 1.66070500 -4.23445500 -3.80878200
H 0.65836800 -3.80303300 -3.78934100
H 2.01560100 -4.27302700 -4.84184000
H 1.61636800 -5.23754200 -3.38758500
H 5.21402100 -2.00371200 1.15134100
H 4.97310900 -1.27736300 -0.81566400
F 0.88573400 -3.05203400 0.26104300
F 2.09333500 -6.36595600 -1.42088200
N 2.76230000 -7.82341500 -2.51598800
S 1.84991300 -9.07319200 -1.71483300
S 4.41022300 -7.48369900 -2.04941600
C 0.21336400 -8.60331800 -2.24282100
O 2.27154700 -10.29307200 -2.42515600
O 1.94366900 -9.02776400 -0.25450200
C 5.19073700 -8.79632500 -2.96662900
O 4.70336900 -6.19849700 -2.68719000
O 4.66397600 -7.67285700 -0.61823100
C -0.29447900 -9.19256700 -3.40464700
C -0.50653700 -7.66735600 -1.49577600
C 5.39153100 -8.62564500 -4.34022500
C 5.55810300 -9.96676000 -2.29710100
C -1.57396900 -8.83365900 -3.82678200
H 0.29729100 -9.91600500 -3.95386000
C -1.78312000 -7.32208500 -1.93936400
H -0.08640300 -7.18810400 -0.61856200
C 5.98236300 -9.66290700 -5.05906200
H 5.09910300 -7.70011100 -4.82324400
C 6.15048900 -10.99229100 -3.03131100
H 5.38603100 -10.06170700 -1.23128400
C -2.31483500 -7.89986400 -3.09496400
H -1.99006100 -9.28197500 -4.72333500
H -2.35146000 -6.58247200 -1.38484100
C 6.35832000 -10.84137400 -4.40624400
H 6.15076800 -9.55145500 -6.12519400
H 6.44924800 -11.90791700 -2.53158800
H -3.30891500 -7.61988900 -3.43110600
H 6.81738000 -11.64628000 -4.97189600

D4-N-4F (Conf. A, Triplet)
E(BS1) = -5170.390266
E(BS2) = -5173.785212
ZPE = 0.858547
H = 0.928826
G = 0.752143
$\langle S^2 \rangle$ = 2.8290
N -0.35031700 -0.86990100 -1.09387000
S 0.43355000 0.04497500 0.04736800
S -0.16415500 -0.51301700 -2.68843800
C -0.08787800 1.73740700 -0.25849800
O 1.90369600 0.01830400 -0.06659100
O -0.15535100 -0.35387300 1.34074200
C -1.44156400 0.70321400 -3.05262000
O -0.54848600 -1.74328000 -3.41315600
O 1.12952700 0.11308900 -3.02691600
C -1.41527000 2.08610200 0.00556300
C 0.82047100 2.65859100 -0.77694500
C -2.77208800 0.38161900 -2.77597500

C -1.09384500 1.93266500 -3.60684400
C -1.83542400 3.38815100 -0.25652200
H -2.10139500 1.34516100 0.40171700
C 0.39017700 3.96325800 -1.02873500
H 1.83757300 2.34640600 -0.98181900
C -3.77034700 1.31093200 -3.05656200
H -3.01646100 -0.57618600 -2.33947300
C -2.10146700 2.86067700 -3.88203900
H -0.05185800 2.15883600 -3.79798900
C -0.93348500 4.32565700 -0.77112800
H -2.86573000 3.67141000 -0.06514300
H 1.08712200 4.69208100 -1.43111000
C -3.43517100 2.55246800 -3.60857500
H -4.80675300 1.06742100 -2.84200300
H -1.83991800 3.82673200 -4.30267700
H -1.26669600 5.33879300 -0.97550200
H -4.21391500 3.27881600 -3.82124700
Cu -0.86345100 -3.07002700 -0.49855300
C -4.50971700 -2.94843400 -2.87492300
C -5.32721700 -2.22303900 -2.02310600
C -4.86552900 -1.91228100 -0.74556800
C -3.58517600 -2.31651800 -0.37091100
C -3.22351400 -3.33279600 -2.46274800
C -3.06298200 -2.10978300 1.00197300
C -3.76844400 -1.41140600 1.98081700
C -3.25715600 -1.38240000 3.27707800
H -3.78886300 -0.85161500 4.06018300
C -2.07595400 -2.05396900 3.55568000
C -1.37808200 -2.70069700 2.52466300
H -4.84585200 -3.23093200 -3.86616800
H -6.31933300 -1.91467000 -2.33584000
H -1.66896300 -2.07194900 4.56035500
N -2.77442800 -2.98904100 -1.23531000
N -1.87821100 -2.70311300 1.27426100
C -0.06871300 -3.37598600 2.79554100
H 0.73836900 -2.68754400 2.53153900
H 0.00890400 -3.63276400 3.85497000
H 0.05630500 -4.26159200 2.17088200
C -2.36692300 -4.13149300 -3.39871400
H -1.95414000 -3.46756400 -4.16214700
H -1.53592300 -4.60056600 -2.88011600
H -2.97799600 -4.89160700 -3.89323600
H -4.69913400 -0.90931400 1.75307400
H -5.50414500 -1.38013500 -0.05390500
F 0.32567300 -5.66424700 0.64009100
F -0.05987700 -4.45166900 -1.65054800
Cu 1.51160600 -4.78439200 -0.53830500
C 3.26951100 -2.28456900 -3.60322000
C 4.10804200 -1.50414100 -2.82089000
C 4.29232500 -1.82431500 -1.47613200
C 3.59198000 -2.90557400 -0.95108700
C 2.57347500 -3.35865800 -3.02746000
C 3.81151000 -3.45394600 0.40484800
C 4.70944100 -2.91523000 1.32347700
C 4.94460000 -3.60470100 2.51232200
H 5.63469800 -3.20058800 3.24579500
C 4.31534000 -4.82276400 2.73735800
C 3.40559600 -5.32502800 1.79414900
H 3.13060200 -2.07378000 -4.65713700
H 4.63133300 -0.65808700 -3.25346100
H 4.51191500 -5.39752500 3.63507100
N 2.73048200 -3.60646400 -1.72000800
N 3.15526600 -4.60542300 0.68672700
C 2.73576400 -6.65271700 1.95357100
H 1.66043300 -6.53787100 1.80865900
H 3.08416700 -7.32110200 1.16052200
H 2.95956100 -7.08516100 2.93028900
C 1.66312100 -4.23114900 -3.83749600
H 0.66108800 -3.79804100 -3.82998500
H 2.02468700 -4.28440300 -4.86752200
H 1.61755900 -5.22740900 -3.39975700
H 5.22364900 -1.98624600 1.11425600
H 4.97206300 -1.24958000 -0.86122900
F 0.90268500 -3.07178800 0.22941600
F 2.05600600 -6.32490600 -1.41774500
N 2.76005700 -7.92929100 -2.62517400
S 1.82490900 -9.09957900 -1.75416000
S 4.37006800 -7.51073800 -2.12083300
C 0.18990300 -8.60229800 -2.26455700
O 2.18639800 -10.36218500 -2.42581700
O 1.94460200 -9.01589000 -0.29691200
C 5.21317300 -8.84091000 -2.95963500
O 4.65275300 -6.24812700 -2.80835200
O 4.60471600 -7.63454100 -0.67894900
C -0.34096700 -9.18476100 -3.41956000
C -0.50997700 -7.65804100 -1.50922700
C 5.48833300 -8.70863800 -4.32441000
C 5.54858200 -9.99097800 -2.23928900
C -1.62117600 -8.81037500 -3.82597500
H 0.23397000 -9.91656700 -3.97561000
C -1.78865700 -7.29859500 -1.93504000
H -0.07307500 -7.18463200 -0.63818000
C 6.12434000 -9.76110000 -4.98019900
H 5.21688700 -7.79911800 -4.84837200
C 6.18517100 -11.03318400 -2.91041200
H 5.31951600 -10.05706000 -1.18193200
C -2.34207500 -7.86887300 -3.08416300
H -2.05371900 -9.25330100 -4.71745900
H -2.34151700 -6.55377200 -1.37172200
C 6.46922000 -10.91884900 -4.27527700
H 6.35140300 -9.67791200 -6.03807100
H 6.45976600 -11.93291700 -2.36952000
H -3.33733400 -7.57750800 -3.40698500
H 6.96315900 -11.73598300 -4.79196000

D4-N-4F (Conf. A, Quintet)
E(BS1) = -5170.386485
E(BS2) = -5173.782032
ZPE = 0.858516
H = 0.928781
G = 0.751458
$\langle S^2 \rangle$ = 6.0156
N -0.47315300 -0.92361200 -1.23272600
S 0.55671900 -0.06992500 -0.25784600
S -0.52926000 -0.61759700 -2.84797900
C 0.08208900 1.65400200 -0.44433500
O 1.97810600 -0.17167600 -0.64242700
O 0.19501200 -0.44932500 1.12228600
C -1.72499800 0.71325200 -3.04733700
O -1.15390800 -1.80999800 -3.45925500
O 0.74415900 -0.13459700 -3.42190600
C -1.18543700 2.04836700 -0.00569200

C 0.96782100 2.55980200 -1.02436300
C -3.05833600 0.46360400 -2.71646000
C -1.31488500 1.96001600 -3.51354000
C -1.56757700 3.37919600 -0.15524300
H -1.85757000 1.31921000 0.43462900
C 0.57681600 3.89404300 -1.16321700
H 1.93799400 2.21398400 -1.36171400
C -3.99443200 1.48728600 -2.84249400
H -3.35438900 -0.51856500 -2.37522000
C -2.25968800 2.98149800 -3.63496600
H -0.27235300 2.12461900 -3.75633400
C -0.68686800 4.30128900 -0.73175100
H -2.55280200 3.69755600 0.17104600
H 1.25719200 4.61080700 -1.61294600
C -3.59458300 2.74824400 -3.29895800
H -5.03348500 1.30044500 -2.58738900
H -1.94812400 3.96072900 -3.98484400
H -0.99031200 5.33744700 -0.84810200
H -4.32438100 3.54701500 -3.39240900
Cu -0.99612300 -3.11924800 -0.47221600
C -4.84832900 -2.91438400 -2.50489000
C -5.52712600 -2.07264700 -1.63760600
C -4.91466100 -1.69372000 -0.44335800
C -3.62977900 -2.15666900 -0.16926600
C -3.55214800 -3.35579000 -2.18983700
C -2.94991800 -1.89388500 1.12156900
C -3.46784200 -1.05212100 2.10318700
C -2.82450100 -0.99926700 3.33978900
H -3.20736400 -0.35483800 4.12447900
C -1.71148200 -1.79688700 3.56322700
C -1.19966300 -2.58991700 2.52535000
H -5.30237000 -3.24754200 -3.43134800
H -6.52658000 -1.72271200 -1.87391400
H -1.21547900 -1.80480700 4.52727700
N -2.96128900 -2.95479100 -1.04615500
N -1.81295500 -2.59821300 1.32779500
C 0.02435200 -3.43086300 2.72921000
H 0.90446700 -2.84990100 2.44327300
H 0.10899200 -3.71873200 3.78026700
H 0.00511400 -4.31135100 2.08565600
C -2.84269200 -4.28676200 -3.12629600
H -2.53732100 -3.73407400 -4.01858500
H -1.94604300 -4.70387400 -2.67722300
H -3.52368700 -5.08645900 -3.43262700
H -4.35532800 -0.45972000 1.92297300
H -5.44052600 -1.06538300 0.26291400
F 0.01170700 -5.63948000 0.33320800
F -0.22011500 -4.24708600 -1.85446100
Cu 1.32142900 -4.79542800 -0.76673200
C 3.32348500 -2.29421700 -3.68800100
C 4.27456300 -1.68320100 -2.88451000
C 4.42517200 -2.09699700 -1.56208700
C 3.58127200 -3.09078800 -1.07530400
C 2.49640600 -3.29617100 -3.15689600
C 3.71983900 -3.69531200 0.26642100
C 4.64273500 -3.25353800 1.21273300
C 4.77266700 -3.96148700 2.40565300
H 5.47766800 -3.62896600 3.16060900
C 4.01052800 -5.10403000 2.60934500
C 3.08476800 -5.51165200 1.63650200
H 3.20151600 -2.01188800 -4.72709000
H 4.91218700 -0.90191300 -3.28475100
H 4.11377300 -5.69270200 3.51365700
N 2.63155300 -3.63331500 -1.86635200
N 2.94418100 -4.77646900 0.52006700
C 2.28510000 -6.76535700 1.78734900
H 1.24291200 -6.57232800 1.52888200
H 2.65743900 -7.50152700 1.06910400
H 2.37287600 -7.16123200 2.80081100
C 1.48362800 -4.00417200 -4.00568800
H 0.52309300 -3.48740900 -3.93605700
H 1.81726300 -4.00949400 -5.04633500
H 1.35182400 -5.02232900 -3.64044800
H 5.24914800 -2.37730000 1.02534300
H 5.18931500 -1.65791600 -0.93453900
F 0.83370100 -3.13308500 0.12946600
F 1.72307500 -6.28347300 -1.77996500
N 2.90684200 -8.04462900 -2.88874200
S 1.94139300 -9.19516800 -2.03359700
S 4.32480800 -7.34330600 -2.17552400
C 0.31400600 -8.73560200 -2.58966400
O 2.33875400 -10.45931000 -2.68246600
O 2.01431500 -9.10390600 -0.57302400
C 5.43176000 -8.63975900 -2.69950300
O 4.57784300 -6.13103900 -2.95895600
O 4.32911200 -7.28853100 -0.71225800
C -0.12875300 -9.25019700 -3.81269700
C -0.47676400 -7.89642200 -1.79734800
C 6.02675200 -8.56069000 -3.96291300
C 5.64035400 -9.72672900 -1.84214500
C -1.40852200 -8.91641900 -4.25226100
H 0.51232900 -9.90276500 -4.39443600
C -1.75603500 -7.58285000 -2.25309700
H -0.10074900 -7.45708900 -0.88315200
C 6.86539400 -9.59697700 -4.36859000
H 5.83949400 -7.70224700 -4.59803000
C 6.47882500 -10.75488000 -2.26535900
H 5.15778400 -9.75500400 -0.87144600
C -2.21943800 -8.08495400 -3.47227200
H -1.77250700 -9.30713900 -5.19703700
H -2.38287500 -6.92798300 -1.65743500
C 7.08764600 -10.68967200 -3.52380500
H 7.34387800 -9.55356800 -5.34153300
H 6.65847800 -11.60518900 -1.61573600
H -3.21546700 -7.82639900 -3.81957300
H 7.73854500 -11.49580700 -3.84788700

DTS-N-3F (Conf. B, Triplet)
E(BS1) = -5170.386303
E(BS2) = -5173.782109
ZPE = 0.857778
H = 0.927656
G = 0.753007
$<S^2>$ = 2.5133
390.7516i
N -0.27578900 -0.84495800 -1.44026700
S 0.90207800 -0.16280900 -0.50356600
S -0.45804200 -0.36705500 -3.00870700
C 0.52226800 1.59182500 -0.40836300
O 2.26432400 -0.28887900 -1.05610800
O 0.68434200 -0.69333100 0.85558800
C -1.54983000 1.06475100 -2.98433400

O -1.23334400 -1.43850800 -3.66752400
O 0.79635600 0.07396000 -3.65323200
C -0.68483400 1.98084900 0.17983700
C 1.43156400 2.52847100 -0.89515600
C -2.90659700 0.86761200 -2.72096500
C -1.03628200 2.33858900 -3.22160600
C -0.98370400 3.33763800 0.27321100
H -1.37760200 1.23097400 0.54554700
C 1.12381000 3.88778100 -0.79219800
H 2.35518300 2.18744600 -1.34867900
C -3.75655700 1.97043800 -2.66153000
H -3.28847900 -0.13508900 -2.59091100
C -1.89488300 3.43775500 -3.16091200
H 0.01785300 2.46209600 -3.43566600
C -0.08035300 4.29035000 -0.21148000
H -1.92212500 3.65358400 0.71850300
H 1.82270300 4.62840200 -1.16889400
C -3.24969800 3.25686200 -2.87424600
H -4.81298000 1.82356100 -2.45684700
H -1.50057300 4.43476400 -3.33015800
H -0.31902900 5.34718900 -0.13829100
H -3.91248400 4.11568100 -2.82399600
Cu -1.20709200 -2.98025600 -0.69997300
C -5.01870400 -2.43098900 -2.70740900
C -5.63480800 -1.53853400 -1.84091900
C -4.97132000 -1.15824600 -0.67784800
C -3.69382100 -1.66517300 -0.43644600
C -3.74102800 -2.93168500 -2.41551300
C -2.96000100 -1.37194500 0.81669200
C -3.37903600 -0.39363100 1.71785900
C -2.72118900 -0.29095200 2.94177600
H -3.02277100 0.46175700 3.66295700
C -1.69563100 -1.17800600 3.23195800
C -1.27641300 -2.11284000 2.27234300
H -5.51776100 -2.76311900 -3.61021600
H -6.62617100 -1.15341900 -2.05614600
H -1.19109700 -1.14845400 4.19098400
N -3.09546800 -2.51795500 -1.30284600
N -1.89354600 -2.16544400 1.07397300
C -0.14536800 -3.04586500 2.58462700
H -0.34163500 -4.03420400 2.17722300
H 0.76556200 -2.67294300 2.11500400
H -0.00328200 -3.10400900 3.66669200
C -3.09624800 -3.93685300 -3.31406400
H -2.17898800 -3.52018700 -3.72832500
H -2.81772400 -4.82511700 -2.74594000
H -3.77982200 -4.21335900 -4.11948800
H -4.19823200 0.27181300 1.47913300
H -5.44986300 -0.49676300 0.03128800
F -0.55562500 -6.13735200 -0.50366300
F -0.60426900 -4.10961900 -2.17360900
Cu 0.87730500 -5.00816200 -1.27139200
C 3.48712700 -2.32694900 -3.60297800
C 4.47716400 -2.04691500 -2.67126800
C 4.43781200 -2.65876000 -1.41989300
C 3.37663600 -3.51618100 -1.13209400
C 2.44898900 -3.20831500 -3.26742100
C 3.27741800 -4.28061700 0.13474000
C 4.05055300 -3.97314400 1.25381000
C 3.95227400 -4.77903500 2.38536900
H 4.53951500 -4.55199600 3.26921700
C 3.10485400 -5.87865400 2.36441200
C 2.32741000 -6.13561800 1.22570700
H 3.50028500 -1.87622500 -4.58857500
H 5.28777300 -1.36896700 -2.91812300
H 3.02241500 -6.53932500 3.22002600
N 2.41460900 -3.75619700 -2.04322200
N 2.41695700 -5.33045300 0.15203200
C 1.37914600 -7.29702200 1.19106300
H 0.36909300 -6.96561700 1.44139600
H 1.32990100 -7.72529400 0.19058300
H 1.69208600 -8.06229500 1.90456800
C 1.37063100 -3.58392300 -4.23835500
H 1.66441200 -3.32511300 -5.25774700
H 1.17784600 -4.65545100 -4.15292800
H 0.44320000 -3.06533800 -3.98617700
H 4.70667200 -3.11257300 1.24856200
H 5.22593000 -2.48187200 -0.69938400
F 0.46351500 -3.58736100 -0.00698200
F 1.27572200 -6.28548200 -2.53539200
N -1.95471500 -7.24297600 0.13776600
S -2.37738400 -8.06890600 -1.34365000
S -3.02845100 -5.98485700 0.69530600
C -1.08103100 -9.28679300 -1.35310300
O -3.66110000 -8.71470000 -1.01931400
O -2.28101700 -7.21472300 -2.52610100
C -4.17877800 -6.98124300 1.62209400
O -2.21871200 -5.20762600 1.63423900
O -3.73186000 -5.31779600 -0.40514600
C -1.36206900 -10.55896500 -0.84813700
C 0.17100200 -8.92993700 -1.85730900
C -3.81277600 -7.40128100 2.90489300
C -5.41294400 -7.30339700 1.05242700
C -0.34485200 -11.51290200 -0.85590000
H -2.35054400 -10.79118600 -0.46844900
C 1.17421800 -9.90084400 -1.84971200
H 0.37405400 -7.92164700 -2.21220400
C -4.71963800 -8.16520800 3.63710100
H -2.84850700 -7.12532300 3.31650300
C -6.30923600 -8.06309900 1.80129100
H -5.65297600 -6.96947700 0.05056800
C 0.91949000 -11.18298100 -1.35426600
H -0.53978400 -12.51011100 -0.47455700
H 2.15957800 -9.64851000 -2.22939500
C -5.96240900 -8.49391100 3.08617900
H -4.45855500 -8.50023900 4.63558300
H -7.27640300 -8.32044400 1.38207400
H 1.70840100 -11.92917800 -1.35551400
H -6.66510000 -9.08882300 3.66142400

D4-N-4F (Conf. B, Triplet)
E(BS1) = -5170.390504
E(BS2) = -5173.789862
ZPE = 0.858569
H = 0.928794
G = 0.753312
$\langle S^2 \rangle$ = 2.8949
N -0.36183700 -0.89659700 -1.35453000
S 0.78977100 -0.13924600 -0.44856000
S -0.52650700 -0.52030900 -2.95043700
C 0.37594400 1.61055300 -0.45446500
O 2.16045700 -0.26238600 -0.98090100

O 0.57410700 -0.60244300 0.93588200
C -1.59239000 0.92923600 -3.02965400
O -1.32270700 -1.61605000 -3.54190000
O 0.73905400 -0.15249800 -3.62037600
C -0.85718500 2.00918400 0.06967200
C 1.28780900 2.53701400 -0.95646400
C -2.94704600 0.77497900 -2.72916000
C -1.06456100 2.17039200 -3.38016400
C -1.17867800 3.36398800 0.08441100
H -1.55253800 1.26707100 0.44606200
C 0.95744600 3.89455700 -0.93208700
H 2.23124000 2.18950100 -1.36171600
C -3.78145100 1.89060400 -2.75346400
H -3.33935300 -0.20651900 -2.50257200
C -1.90795800 3.28288200 -3.40238900
H -0.01179200 2.25914900 -3.61789700
C -0.27205900 4.30611400 -0.41453400
H -2.13733300 3.68630300 0.47911100
H 1.65844000 4.62664300 -1.32146000
C -3.26088900 3.14628000 -3.08422700
H -4.83631900 1.77755600 -2.52091900
H -1.50373400 4.25650800 -3.66101100
H -0.52844900 5.36128600 -0.40288300
H -3.91142600 4.01575800 -3.09950500
Cu -1.20022900 -3.11775600 -0.53501600
C -5.02385000 -2.56427000 -2.52859100
C -5.61862300 -1.62942800 -1.69151000
C -4.94037600 -1.21583800 -0.54826300
C -3.66621000 -1.72963500 -0.29887900
C -3.74872200 -3.06620500 -2.22840100
C -2.92348400 -1.41891000 0.94458800
C -3.33510900 -0.42196800 1.82858000
C -2.67447000 -0.29702800 3.04884000
H -2.97458600 0.46847900 3.75708000
C -1.64432400 -1.17441700 3.34908000
C -1.22982900 -2.12510300 2.40354800
H -5.53709600 -2.92729600 -3.41143800
H -6.60692500 -1.23931600 -1.91190200
H -1.13005200 -1.12267200 4.30193100
N -3.08532900 -2.61311800 -1.14363100
N -1.86042100 -2.21397200 1.21471300
C -0.07550600 -3.02898800 2.71059800
H -0.26979900 -4.03973000 2.35905900
H 0.80855300 -2.66095700 2.18788900
H 0.11232100 -3.03898400 3.78703800
C -3.12113000 -4.12823600 -3.07201400
H -2.18632900 -3.75841000 -3.49083300
H -2.87404400 -4.99347700 -2.45419500
H -3.80043100 -4.42906300 -3.87230100
H -4.15394700 0.24065700 1.58076900
H -5.40677200 -0.52523500 0.14101100
F -0.22327900 -6.27589400 -0.36216400
F -0.53361700 -4.18766300 -2.04208400
Cu 0.96988100 -5.07228600 -1.17035200
C 3.28584700 -2.22611200 -3.50529000
C 4.26282500 -1.87310400 -2.58791500
C 4.30059200 -2.50544000 -1.34600700
C 3.32188600 -3.44939200 -1.05116300
C 2.33042400 -3.19909600 -3.17294400
C 3.32528600 -4.26123200 0.18435600
C 4.11146200 -3.95812400 1.29359400
C 4.12481200 -4.84450000 2.36887000
H 4.72363700 -4.62592600 3.24696400
C 3.38238300 -6.01559100 2.29706000
C 2.58198100 -6.27019800 1.17227500
H 3.23965600 -1.76260200 -4.48310900
H 5.00529100 -1.12242800 -2.83744300
H 3.39928500 -6.73977000 3.10361000
N 2.36116300 -3.75348500 -1.95125300
N 2.55536900 -5.37844500 0.16648200
C 1.76394300 -7.52311000 1.07770600
H 0.73607200 -7.32451500 1.38664700
H 1.71509800 -7.87368200 0.04722900
H 2.19606200 -8.29682800 1.71618600
C 1.29214600 -3.65076200 -4.15408100
H 1.61602100 -3.42185200 -5.17169500
H 1.12799300 -4.72208000 -4.03364400
H 0.34666100 -3.14240800 -3.95600500
H 4.69576500 -3.04749500 1.32062200
H 5.08433800 -2.27679200 -0.63552600
F 0.51111700 -3.66351500 0.12890400
F 1.41280900 -6.29058300 -2.49216000
N -1.81268900 -7.59620800 0.52052700
S -2.39599400 -8.26333100 -0.95994900
S -2.48559400 -6.13427500 1.15732600
C -1.05102600 -9.37297700 -1.31372000
O -3.57455900 -9.04144900 -0.53373600
O -2.54625200 -7.28874700 -2.04210000
C -4.06737900 -6.74666000 1.70262900
O -1.64689400 -5.85716200 2.32714300
O -2.73418900 -5.09123300 0.15437600
C -1.18977100 -10.71710000 -0.95700100
C 0.09670800 -8.86978700 -1.92965300
C -4.13431700 -7.46951700 2.89753800
C -5.19374600 -6.50268500 0.91127600
C -0.13842500 -11.58975600 -1.23657900
H -2.09940600 -11.06474500 -0.48082800
C 1.13723900 -9.76103800 -2.19525700
H 0.20846000 -7.81704700 -2.16499800
C -5.37313400 -7.95647200 3.31033200
H -3.23973500 -7.63294200 3.48790100
C -6.42450000 -6.99414000 1.34139500
H -5.09970800 -5.93954100 -0.01018700
C 1.02227200 -11.11152200 -1.85282600
H -0.22632400 -12.63911600 -0.97379600
H 2.04202900 -9.38945800 -2.66617600
C -6.51211500 -7.72031500 2.53385700
H -5.45011700 -8.51711200 4.23616900
H -7.31377700 -6.81188800 0.74692700
H 1.83910100 -11.79486200 -2.06528400
H -7.47401300 -8.10316800 2.86060800

1
E(BS1) = -784.091640
E(BS2) = -784.203798
ZPE = 0.148029
H = 0.157588
G = 0.116473
C 0.01839900 0.11034700 -0.04410700
C 1.35048700 0.18583200 0.29513800
C 1.90161200 1.49501400 0.17081600
C 0.98959000 2.41692400 -0.26467800

S -0.56550400 1.69364600 -0.53609300
H 1.91273400 -0.66981400 0.65049100
H 2.93135400 1.74015700 0.40404300
H 1.13271700 3.47367200 -0.44393600
C -0.85599800 -1.07025600 -0.03774900
C -2.24459700 -0.96173700 0.16158900
C -0.30802400 -2.35332200 -0.22702900
C -3.05554200 -2.09545600 0.17140900
H -2.68991200 0.01473100 0.32881200
C -1.11952200 -3.48563700 -0.20371200
H 0.75630500 -2.45817800 -0.41190400
C -2.49765300 -3.36340500 -0.00684100
H -4.12461800 -1.98748000 0.32869100
H -0.67538100 -4.46546400 -0.35265200
H -3.12947700 -4.24623300 0.00456600

D5-N-4F (Triplet)
E(BS1) = -5954.492587
E(BS2) = -5958.008035
ZPE = 1.008444
H = 1.089033
G = 0.888607
<S^2> = 2.8603
N -0.70235700 -0.82621100 -1.79992500
S 0.25013000 0.04300000 -0.76040300
S -0.82577700 -0.37491700 -3.37875500
C -0.44440900 1.70086300 -0.74041500
O 1.65686600 0.16583000 -1.18895700
O -0.00742900 -0.52528200 0.57720500
C -2.12438200 0.86988100 -3.43449100
O -1.37340400 -1.55518600 -4.08118700
O 0.39438700 0.25136200 -3.92740500
C -1.75331900 1.86993100 -0.27877900
C 0.32029000 2.78042600 -1.17726900
C -3.42475900 0.48854200 -3.09844900
C -1.82317100 2.18123700 -3.79492400
C -2.30146200 3.14973800 -0.25910500
H -2.32961800 1.00954200 0.04460300
C -0.23707600 4.06131500 -1.14627800
H 1.32817500 2.60805700 -1.53685300
C -4.43931500 1.44261900 -3.11320600
H -3.63520100 -0.53894800 -2.83713500
C -2.84644800 3.13192200 -3.80497300
H -0.80378600 2.44818900 -4.04592600
C -1.54356700 4.24445300 -0.69024000
H -3.32078500 3.29479200 0.08488300
H 0.34773600 4.91169900 -1.48342800
C -4.14977900 2.76592400 -3.46353600
H -5.45326300 1.15329300 -2.85293800
H -2.62053100 4.15941800 -4.07244100
H -1.97681500 5.24007200 -0.67468600
H -4.94058000 3.51015300 -3.46956500
Cu -1.03679300 -3.10058200 -1.24242800
C -4.94371900 -2.99569300 -3.18846800
C -5.67351300 -2.32172600 -2.22293400
C -5.06656000 -2.02427800 -1.00333100
C -3.73981200 -2.39701900 -0.79980400
C -3.60668300 -3.35265500 -2.94350500
C -3.05723700 -2.20337500 0.50206300
C -3.63205500 -1.51491400 1.56887800
C -2.96970400 -1.51661900 2.79610700
H -3.39649300 -0.99201700 3.64481600
C -1.77771000 -2.21422400 2.92477000
C -1.21420700 -2.84754100 1.80693800
H -5.38577800 -3.26149500 -4.14218400
H -6.70530900 -2.03889400 -2.40347200
H -1.25895900 -2.26191700 3.87556900
N -3.02454400 -3.03357500 -1.76857200
N -1.85305000 -2.80831100 0.62279000
C 0.10045000 -3.55831800 1.90752700
H 0.89312000 -2.86276100 1.62155700
H 0.26733600 -3.89641600 2.93332500
H 0.14843400 -4.39419300 1.20874100
C -2.85683100 -4.10994800 -3.99709800
H -2.70772600 -3.47049000 -4.86997300
H -1.88346600 -4.43921000 -3.64696000
H -3.45232700 -4.97185200 -4.31070100
H -4.57665700 -0.99830000 1.46088900
H -5.62897900 -1.52764700 -0.22443200
F 0.20507200 -5.62599600 -0.55704000
F -0.26624600 -4.19882200 -2.66248100
Cu 1.37212300 -4.65324300 -1.69942300
C 2.91199700 -1.71219400 -4.47776900
C 3.86538100 -1.09972100 -3.68053100
C 4.17511200 -1.64618500 -2.43522100
C 3.48555500 -2.77880300 -2.01418900
C 2.23835500 -2.85381000 -4.01406600
C 3.79187600 -3.50864600 -0.76549800
C 4.80642000 -3.12612300 0.11039700
C 5.08681200 -3.93552600 1.20893600
H 5.86767200 -3.65236200 1.90721400
C 4.37589500 -5.11434600 1.38822200
C 3.35748900 -5.46417700 0.48808900
H 2.66225200 -1.32217300 -5.45703600
H 4.37928000 -0.20806900 -4.02392000
H 4.59236700 -5.77830000 2.21722200
N 2.52632600 -3.32807600 -2.79299000
N 3.07700700 -4.63678000 -0.53558400
C 2.60903600 -6.75157400 0.61656200
H 1.53877100 -6.57232000 0.51152300
H 2.89718700 -7.40727600 -0.20926600
H 2.83683400 -7.23599000 1.56782800
C 1.21963400 -3.55065600 -4.85909000
H 0.24068100 -3.09185400 -4.70351400
H 1.49313800 -3.46811400 -5.91235600
H 1.15651400 -4.59838800 -4.57654600
H 5.37389800 -2.22122300 -0.06171000
H 4.93746700 -1.19452300 -1.81470000
F 0.81026600 -3.05243500 -0.70645300
F 1.85010600 -6.07352500 -2.80127300
N 2.63791100 -7.69360100 -4.02187900
S 1.76055500 -8.93644200 -3.19679100
S 4.23730700 -7.25128500 -3.49618000
C 0.07943900 -8.47812900 -3.57096500
O 2.11487300 -10.14053200 -3.97104600
O 1.96394000 -8.94725400 -1.74479500
C 5.10666900 -8.66422400 -4.15037500
O 4.55633800 -6.06726700 -4.30116400
O 4.41500000 -7.22082300 -2.04329100
C -0.52243100 -9.06205200 -4.69014000
C -0.60075300 -7.59689200 -2.72695900
C 5.53328500 -8.64830400 -5.48156000

C 5.30985100 -9.76783400 -3.31532100
C -1.85557500 -8.75962000 -4.96389200
H 0.03982300 -9.73947000 -5.32199400
C -1.93375200 -7.30947100 -3.01978800
H -0.11836100 -7.12276400 -1.88110300
C 6.18629100 -9.77211100 -5.98477600
H 5.36891700 -7.77283200 -6.09886200
C 5.96406000 -10.88248600 -3.83464300
H 4.96156400 -9.74585100 -2.28891900
C -2.55956000 -7.88825600 -4.12669000
H -2.34112500 -9.19925300 -5.82887800
H -2.47576000 -6.61900800 -2.38226600
C 6.39791700 -10.88474100 -5.16456600
H 6.52996500 -9.77883100 -7.01396900
H 6.13520900 -11.74816300 -3.20327500
H -3.59873400 -7.65765600 -4.34293800
H 6.90483300 -11.75800900 -5.56327900
C 0.33343900 -5.47429600 -7.87223800
C 1.29386400 -4.69041900 -8.47478100
C 2.61421400 -4.94911200 -8.00049200
C 2.65285600 -5.92847000 -7.04371100
S 1.07566100 -6.56260500 -6.71624000
H 1.05460100 -3.92430300 -9.20368500
H 3.49300900 -4.41134200 -8.33828800
H 3.49685000 -6.27894600 -6.46884300
C -1.11983900 -5.45493200 -8.08158600
C -2.01024900 -5.80305100 -7.04873600
C -1.65615000 -5.07403700 -9.32618500
C -3.38808800 -5.77995200 -7.25842500
H -1.62262400 -6.07826300 -6.07350300
C -3.03517100 -5.03468200 -9.52611600
H -0.98515300 -4.82553200 -10.14274900
C -3.90929100 -5.39171400 -8.49513400
H -4.05686200 -6.05836400 -6.44911900
H -3.42800900 -4.73741000 -10.49430200
H -4.98313100 -5.36943200 -8.65529200

D5-N-4F (Quintet)

E(BS1) = -5954.488806
E(BS2) = -5958.004977
ZPE = 1.008239
H = 1.088859
G = 0.887946
$\langle S^2 \rangle$ = 6.0151
N -0.73447000 -0.83344100 -1.80486200
S 0.24397100 0.03342700 -0.79026700
S -0.87482400 -0.40443400 -3.38861700
C -0.44629400 1.69302000 -0.75324000
O 1.64069200 0.15421200 -1.25155200
O 0.01740600 -0.53258500 0.55414000
C -2.14802400 0.86665000 -3.44114700
O -1.45730000 -1.58340200 -4.06418200
O 0.34848500 0.18840300 -3.96662000
C -1.75538200 1.86109800 -0.29172500
C 0.32389100 2.77576800 -1.17200900
C -3.45485800 0.51161900 -3.10175200
C -1.82034300 2.17305600 -3.79682100
C -2.29850600 3.14260000 -0.25527700
H -2.33621300 0.99847200 0.01715000
C -0.22843300 4.05841400 -1.12443000
H 1.33260100 2.60475000 -1.53003400
C -4.44928000 1.48691000 -3.10974000
H -3.68582600 -0.51246300 -2.84403100
C -2.82334800 3.14506700 -3.80000400
H -0.79642400 2.41946100 -4.05032900
C -1.53538800 4.24032800 -0.66934500
H -3.31819400 3.28678000 0.08801500
H 0.36076100 4.91112600 -1.44784300
C -4.13314600 2.80508600 -3.45605800
H -5.46835900 1.21804500 -2.84748300
H -2.57671400 4.16867300 -4.06407300
H -1.96481800 5.23732900 -0.64100200
H -4.90815300 3.56577700 -3.45694500
Cu -1.07359000 -3.10939000 -1.22836500
C -5.01126400 -2.94781100 -3.11299700
C -5.71157500 -2.25155200 -2.14134600
C -5.07894500 -1.95640300 -0.93417700
C -3.75765200 -2.35649000 -0.74826400
C -3.67822300 -3.33046100 -2.88545800
C -3.04953400 -2.16919000 0.54074400
C -3.59505500 -1.47143200 1.61670400
C -2.91056900 -1.48064200 2.83173600
H -3.31446100 -0.94874500 3.68707700
C -1.72668000 -2.19524900 2.94033200
C -1.19291700 -2.83887900 1.81399800
H -5.47334600 -3.21117700 -4.05786900
H -6.73983000 -1.94807200 -2.30811500
H -1.19175400 -2.24921800 3.88177600
N -3.07177000 -3.01608600 -1.72237100
N -1.85206200 -2.79114300 0.64142600
C 0.11112700 -3.57180500 1.89207100
H 0.91175600 -2.88893100 1.59795700
H 0.28703100 -3.91810600 2.91362100
H 0.13352800 -4.40453500 1.18824100
C -2.96041700 -4.11624700 -3.94039900
H -2.88284100 -3.52133400 -4.85312400
H -1.95941000 -4.39731000 -3.62835800
H -3.54316900 -5.01230900 -4.17452400
H -4.53455100 -0.94244300 1.52523500
H -5.61783600 -1.44001100 -0.15145600
F 0.13094200 -5.61200600 -0.59268200
F -0.32388300 -4.15379900 -2.68850800
Cu 1.31857600 -4.65456400 -1.73865200
C 2.89222300 -1.74468900 -4.53613900
C 3.85411800 -1.13734000 -3.74519300
C 4.15594700 -1.67477600 -2.49388800
C 3.44938300 -2.79235700 -2.06089600
C 2.20213600 -2.87198700 -4.06100800
C 3.74472100 -3.51407700 -0.80503000
C 4.75053400 -3.12563900 0.07830300
C 5.02034700 -3.92752600 1.18500700
H 5.79397900 -3.63918800 1.88919700
C 4.30715700 -5.10471800 1.36609100
C 3.29676400 -5.45990800 0.45893400
H 2.64999000 -1.36279900 -5.52059600
H 4.38187700 -0.25778200 -4.09868800
H 4.51427000 -5.76218800 2.20262400
N 2.48248800 -3.33527100 -2.83428800
N 3.02785600 -4.64077000 -0.57387400
C 2.54188500 -6.74312400 0.59186800
H 1.47323400 -6.55952600 0.47478300
H 2.83599100 -7.40818700 -0.22440600

H 2.75705200 -7.21919400 1.55027000
C 1.17733800 -3.56696000 -4.90136500
H 0.20360900 -3.09210300 -4.75835300
H 1.45815700 -3.50377600 -5.95433700
H 1.09883300 -4.60927000 -4.60223400
H 5.31834700 -2.22089400 -0.09366300
H 4.92664700 -1.22929500 -1.87913900
F 0.79002500 -3.06320100 -0.72768600
F 1.73501200 -6.06554600 -2.85594300
N 2.68751100 -7.91786800 -4.11740300
S 1.76691800 -9.05705500 -3.20223100
S 4.20766500 -7.31478600 -3.53077600
C 0.10430000 -8.56347900 -3.59983500
O 2.07731400 -10.31922900 -3.90049300
O 1.97511900 -8.98728800 -1.75314400
C 5.18562500 -8.67984200 -4.13038900
O 4.47136800 -6.12823000 -4.35096100
O 4.33566400 -7.24840300 -2.07464800
C -0.49265400 -9.12978700 -4.73158300
C -0.56824000 -7.66746300 -2.76381300
C 5.72643300 -8.62172900 -5.41903300
C 5.35521900 -9.79364600 -3.29879400
C -1.81220300 -8.79424400 -5.02652300
H 0.06352300 -9.81915500 -5.35588500
C -1.88853400 -7.34742200 -3.07788700
H -0.08601200 -7.20455800 -1.91292400
C 6.46636100 -9.70894200 -5.87942500
H 5.58078200 -7.73954300 -6.03179500
C 6.09442200 -10.87238200 -3.77657300
H 4.91732000 -9.80494400 -2.30684100
C -2.50834600 -7.90746700 -4.19763800
H -2.29456100 -9.21965900 -5.90031200
H -2.42354800 -6.64597900 -2.44701800
C 6.64619400 -10.82986700 -5.06209900
H 6.90151600 -9.68238500 -6.87301900
H 6.24135100 -11.74470900 -3.14838300
H -3.53719400 -7.65049400 -4.43241700
H 7.21963200 -11.67560900 -5.42863000
C 0.32398600 -5.49516500 -7.87041400
C 1.31023700 -4.74721500 -8.47683000
C 2.62401200 -5.06841800 -8.02268000
C 2.63195000 -6.06048900 -7.07846800
S 1.03251000 -6.62719300 -6.73567500
H 1.09505100 -3.96381300 -9.19476200
H 3.52133300 -4.56728100 -8.36801100
H 3.46749600 -6.46323900 -6.52657400
C -1.12915800 -5.41067300 -8.06431600
C -2.02472800 -5.71488600 -7.02203300
C -1.65874000 -5.01028600 -9.30569100
C -3.40214200 -5.63095700 -7.22062500
H -1.64016800 -6.00203300 -6.04876900
C -3.03623600 -4.91003300 -9.49427700
H -0.98409800 -4.79469300 -10.12861500
C -3.91611200 -5.22467500 -8.45455400
H -4.07659900 -5.87456600 -6.40500300
H -3.42381300 -4.59867600 -10.46015000
H -4.98929900 -5.15537400 -8.60546500

D6-N-4F (Triplet)
E(BS1) = -5954.505644
E(BS2) = -5958.027465
ZPE = 1.008094
H = 1.088678
G = 0.889691
$\langle S^2 \rangle$ = 3.0238
N -0.49139000 -0.67822700 -1.02449600
S 0.50385000 -0.79335100 0.29064700
S 0.08452700 -0.09938600 -2.45220700
C 1.07371100 0.87991000 0.62410800
O 1.70725200 -1.62114900 0.08104100
O -0.37945200 -1.15523900 1.41787700
C -0.06701600 1.69154400 -2.34156200
O -0.89249600 -0.50654300 -3.48332100
O 1.50985600 -0.39771000 -2.70509600
C 0.13167500 1.84627600 0.98876400
C 2.42806400 1.18856500 0.51459800
C -1.34717700 2.23979500 -2.23614900
C 1.06860300 2.49725200 -2.35386700
C 0.56087200 3.14601300 1.24531900
H -0.91761400 1.57983200 1.05832100
C 2.84938300 2.49390400 0.78112300
H 3.12793600 0.41484800 0.22088400
C -1.48893500 3.62117600 -2.12929900
H -2.21339800 1.59158600 -2.24365600
C 0.91727200 3.88175000 -2.24523400
H 2.04799100 2.04095400 -2.43147700
C 1.91859900 3.46920800 1.14317200
H -0.16149400 3.90897400 1.51840900
H 3.90204000 2.74743500 0.69970600
C -0.35603200 4.44273100 -2.13074100
H -2.48103600 4.05548800 -2.04640000
H 1.79644400 4.51856800 -2.24139800
H 2.24813600 4.48475500 1.34163700
H -0.46757700 5.51941300 -2.04251700
Cu -2.24233400 -2.36702000 -1.10672900
C -4.64838100 0.38833300 -3.44345200
C -4.96559100 1.31690000 -2.46052000
C -4.61901000 1.05425700 -1.13638900
C -3.93438800 -0.12496000 -0.84481900
C -3.95963600 -0.78483900 -3.10167100
C -3.62425200 -0.55604000 0.53943300
C -3.91467200 0.20602000 1.66858000
C -3.72533700 -0.37024700 2.92510900
H -3.94738100 0.19863400 3.82224800
C -3.28109500 -1.68192700 3.01485100
C -2.95969100 -2.39028500 1.84774800
H -4.93120900 0.55229000 -4.47671900
H -5.49328500 2.23025600 -2.71496400
H -3.16178300 -2.16624600 3.97751600
N -3.59203500 -1.00083500 -1.82270000
N -3.11442600 -1.80417000 0.64814400
C -2.44210100 -3.79554800 1.90416700
H -1.34995200 -3.76943000 1.91269500
H -2.80083800 -4.29450000 2.80790700
H -2.73647300 -4.34391000 1.00822000
C -3.63658200 -1.82384100 -4.13044600
H -2.55780600 -1.85454200 -4.28449500
H -3.93131300 -2.81346300 -3.77468300
H -4.14674000 -1.60241900 -5.06979300
H -4.29663800 1.21487800 1.58177600
H -4.89026300 1.74929300 -0.35283200
F -3.03946100 -4.71561900 -1.14936700

F -1.64870300 -3.24284600 -2.74366700
Cu -1.23232600 -4.86785300 -1.77318000
C 2.74136900 -3.62124700 -2.88076800
C 3.42506900 -3.97278200 -1.72933000
C 2.79382100 -4.76048200 -0.76592800
C 1.47583100 -5.13877100 -0.97854800
C 1.40293900 -4.01318000 -3.04829200
C 0.72380000 -6.05796100 -0.10212300
C 1.27869900 -6.73553900 0.98317100
C 0.48904600 -7.64799000 1.67818400
H 0.90149300 -8.18346600 2.52719300
C -0.81544000 -7.89707900 1.25953600
C -1.33143900 -7.20197000 0.15957400
H 3.21690900 -3.04842800 -3.66659800
H 4.45431500 -3.66177100 -1.58536500
H -1.43578200 -8.63200800 1.75982400
N 0.81251000 -4.72812200 -2.08454000
N -0.56029500 -6.29104000 -0.45902900
C -2.69826400 -7.46224100 -0.39907600
H -3.37273200 -6.64539300 -0.13645800
H -2.63708700 -7.46471400 -1.49029100
H -3.08935100 -8.41319500 -0.03354500
C 0.63224600 -3.67032600 -4.28631000
H 0.20677800 -2.66769100 -4.19474600
H 1.30624900 -3.71445500 -5.14342000
H -0.17961800 -4.38370500 -4.42053100
H 2.31218900 -6.57701000 1.25943200
H 3.32817700 -5.08584100 0.11625200
F -0.88209200 -3.54621600 -0.36374900
F -1.58712800 -6.03450600 -3.16885400
N 2.20839400 -7.66914000 -3.83191700
S 3.42851900 -8.06707500 -2.82112400
S 2.37857300 -6.85956900 -5.25054900
C 3.46127200 -9.87107400 -2.86390300
O 4.74771600 -7.58811500 -3.29279400
O 3.04237700 -7.71672600 -1.43943600
C 3.25220300 -7.94481200 -6.39065500
O 1.00004900 -6.72397500 -5.76287000
O 3.19674000 -5.63952600 -5.12356100
C 3.41497900 -10.55806600 -4.08064800
C 3.61801600 -10.55705800 -1.65701400
C 2.51567100 -8.78601400 -7.22867900
C 4.64915500 -7.97847700 -6.37054000
C 3.53325700 -11.94791900 -4.08369300
H 3.27710200 -10.02096300 -5.00903200
C 3.73525800 -11.94748500 -1.67129600
H 3.63108600 -10.00301700 -0.72566100
C 3.19227400 -9.69608200 -8.04225000
H 1.43453700 -8.71891200 -7.24389900
C 5.31364500 -8.89411500 -7.18727200
H 5.19291600 -7.31799500 -5.70766200
C 3.69543800 -12.64312100 -2.88252700
H 3.48999400 -12.48536400 -5.02599500
H 3.84162100 -12.48776700 -0.73635500
C 4.58865200 -9.75586900 -8.01546300
H 2.62988200 -10.35403700 -8.69766400
H 6.39839600 -8.93514600 -7.17505900
H 3.77453700 -13.72540700 -2.88801900
H 5.11170500 -10.46893200 -8.64552700
C 0.34162900 -12.16518900 -2.14149100
C 0.15019600 -10.91796000 -2.82961400
C 0.48421500 -12.21364100 -0.73122200
C 0.41511900 -13.38222200 -2.86621500
C 0.05302500 -10.69483200 -4.22301400
S 0.01501100 -9.38619600 -1.96570700
C 0.69202100 -13.42036600 -0.08071400
H 0.44379200 -11.29883500 -0.14841000
C 0.62295800 -14.58539400 -2.20739000
H 0.31333100 -13.37987000 -3.94450000
C -0.11114200 -9.36256600 -4.55722200
H 0.11506600 -11.49154200 -4.95203500
C -0.11590300 -8.50670900 -3.43536000
C 0.76435800 -14.61161100 -0.81452800
H 0.80321600 -13.43813600 0.99837100
H 0.67864300 -15.50702000 -2.77728700
H -0.19295700 -8.96763600 -5.55942800
H -0.37598900 -7.45073600 -3.41320300
H 0.93003300 -15.55460000 -0.30332600

D6-N-4F (Quintet)
E(BS1) = -5954.505628
E(BS2) = -5958.027447
ZPE = 1.008099
H = 1.08868
G = 0.889238
$\langle S^2 \rangle$ = 6.0249
N -0.49148400 -0.67819300 -1.02433200
S 0.50364300 -0.79364600 0.29086600
S 0.08466100 -0.09936700 -2.45196100
C 1.07376200 0.87947200 0.62460800
O 1.70694000 -1.62159500 0.08124200
O -0.37980800 -1.15555300 1.41797500
C -0.06634200 1.69159600 -2.34112000
O -0.89245400 -0.50611200 -3.48314800
O 1.50990600 -0.39810100 -2.70483500
C 0.13186100 1.84598300 0.98922500
C 2.42819900 1.18786300 0.51538400
C -1.34634500 2.24022600 -2.23576100
C 1.06952800 2.49695500 -2.35319200
C 0.56128400 3.14559400 1.24604200
H -0.91749800 1.57975500 1.05854300
C 2.84974400 2.49307500 0.78217500
H 3.12796500 0.41404400 0.22167900
C -1.48768500 3.62163600 -2.12873300
H -2.21276400 1.59228200 -2.24343800
C 0.91861500 3.88148500 -2.24438400
H 2.04878400 2.04036700 -2.43076500
C 1.91909700 3.46852000 1.14419400
H -0.16097300 3.90866800 1.51910400
H 3.90246800 2.74639600 0.70098400
C -0.35452900 4.44284200 -2.12994600
H -2.47966000 4.05624600 -2.04587000
H 1.79798200 4.51803100 -2.24036900
H 2.24881000 4.48397000 1.34286700
H -0.46575000 5.51954600 -2.04158300
Cu -2.24270100 -2.36672800 -1.10700000
C -4.64793200 0.38937300 -3.44362400
C -4.96497600 1.31792100 -2.46061900
C -4.61855900 1.05506100 -1.13648600
C -3.93426100 -0.12436000 -0.84498800
C -3.95950600 -0.78400600 -3.10191200
C -3.62432400 -0.55568000 0.53923400

C -3.91458100 0.20632200 1.66845800
C -3.72545600 -0.37015000 2.92492700
H -3.94737500 0.19868700 3.82212400
C -3.28159600 -1.68196800 3.01453300
C -2.96033900 -2.39027800 1.84735900
H -4.93064300 0.55350800 -4.47689500
H -5.49241800 2.23143700 -2.71501100
H -3.16247000 -2.16643900 3.97714400
N -3.59206400 -1.00021900 -1.82293600
N -3.11484800 -1.80396700 0.64782500
C -2.44317300 -3.79570700 1.90364000
H -1.35101700 -3.76993000 1.91247300
H -2.80228700 -4.29471400 2.80720000
H -2.73748200 -4.34383800 1.00752900
C -3.63662000 -1.82300000 -4.13074900
H -2.55783000 -1.85401400 -4.28463800
H -3.93171300 -2.81256900 -3.77513000
H -4.14657300 -1.60133200 -5.07014900
H -4.29626200 1.21529700 1.58175700
H -4.88968800 1.75008700 -0.35287700
F -3.03979100 -4.71561800 -1.15021300
F -1.64887400 -3.24232300 -2.74390600
Cu -1.23252800 -4.86752000 -1.77359700
C 2.74156400 -3.62145100 -2.88072900
C 3.42507200 -3.97299100 -1.72917600
C 2.79350700 -4.76025700 -0.76562600
C 1.47543200 -5.13821500 -0.97828300
C 1.40301800 -4.01298800 -3.04826300
C 0.72314500 -6.05728100 -0.10194700
C 1.27756700 -6.73446200 0.98383200
C 0.48780400 -7.64713200 1.67844000
H 0.89990200 -8.18234300 2.52778500
C -0.81632700 -7.89675500 1.25899900
C -1.33193500 -7.20183400 0.15873700
H 3.21733600 -3.04892700 -3.66663400
H 4.45442400 -3.66231500 -1.58522900
H -1.43672300 -8.63184400 1.75898600
N 0.81234300 -4.72757100 -2.08440500
N -0.56068700 -6.29076400 -0.45950300
C -2.69850500 -7.46229100 -0.40047800
H -3.37328800 -6.64576800 -0.13764700
H -2.63706200 -7.46424500 -1.49167300
H -3.08941100 -8.41352000 -0.03546900
C 0.63238800 -3.67012200 -4.28631900
H 0.20648000 -2.66769500 -4.19455200
H 1.30647900 -3.71381000 -5.14337200
H -0.17917000 -4.38381800 -4.42075300
H 2.31079300 -6.57546300 1.26082200
H 3.32772500 -5.08563800 0.11663400
F -0.88270500 -3.54620800 -0.36393800
F -1.58654000 -6.03385900 -3.16969800
N 2.20582400 -7.66941900 -3.83309100
S 3.42593300 -8.06655200 -2.82189900
S 2.37608600 -6.85998000 -5.25183500
C 3.46041900 -9.87052800 -2.86479900
O 4.74486700 -7.58627600 -3.29295600
O 3.03887300 -7.71677100 -1.44033400
C 3.25066100 -7.94490400 -6.39152500
O 0.99769500 -6.72513900 -5.76467600
O 3.19364300 -5.63955300 -5.12466500
C 3.41482200 -10.55758800 -4.08152600
C 3.61767800 -10.55636300 -1.65788600
C 2.51483500 -8.78651500 -7.22975900
C 4.64762300 -7.97788900 -6.37091000
C 3.53423900 -11.94734800 -4.08452700
H 3.27656500 -10.02064200 -5.00993500
C 3.73613100 -11.94668300 -1.67212900
H 3.63017100 -10.00230800 -0.72653400
C 3.19217200 -9.69631500 -8.04302000
H 1.43367200 -8.71994200 -7.24538500
C 5.31285000 -8.89326600 -7.18733700
H 5.19083600 -7.31710400 -5.70788100
C 3.69691500 -12.64238900 -2.88333800
H 3.49147700 -12.48484300 -5.02682400
H 3.84287700 -12.48684500 -0.73716300
C 4.58856800 -9.75543000 -8.01572300
H 2.63033400 -10.35458900 -8.69858900
H 6.39761500 -8.93377600 -7.17472800
H 3.77690100 -13.72461200 -2.88879300
H 5.11219100 -10.46829000 -8.64554300
C 0.34280400 -12.16627700 -2.14034400
C 0.15067700 -10.91959100 -2.82932000
C 0.48617600 -12.21363500 -0.73013000
C 0.41628600 -13.38382000 -2.86418900
C 0.05394300 -10.69736400 -4.22285900
S 0.01363700 -9.38744100 -1.96636800
C 0.69475900 -13.41980500 -0.07882700
H 0.44588500 -11.29839600 -0.14799200
C 0.62498300 -14.58642500 -2.20458300
H 0.31370000 -13.38232900 -3.94240300
C -0.11130300 -9.36541800 -4.55792200
H 0.11721400 -11.49442000 -4.95139700
C -0.11781500 -8.50899200 -3.43657600
C 0.76716000 -14.61156800 -0.81178200
H 0.80656700 -13.43671600 1.00021000
H 0.68065600 -15.50845700 -2.77382700
H -0.19283900 -8.97113800 -5.56040900
H -0.37944400 -7.45324000 -3.41499700
H 0.93346800 -15.55411600 -0.29997300

DTSN-N-4F (Triplet)
E(BS1) = -5954.501304
E(BS2) = -5958.021335
ZPE = 1.007753
H = 1.087537
G = 0.890449
$\langle S^2 \rangle$ = 3.0306
331.6630i
N -0.46756600 -0.71418500 -1.01924100
S 0.51207100 -0.81866300 0.30872300
S 0.12077700 -0.12601000 -2.43744200
C 1.05994500 0.86064200 0.64890400
O 1.72642600 -1.63394700 0.11374600
O -0.38107700 -1.18947100 1.42504800
C -0.06489000 1.66164900 -2.33004800
O -0.83136000 -0.55282400 -3.48418100
O 1.55539800 -0.39809100 -2.66657100
C 0.10375000 1.81497300 1.00840200
C 2.41127000 1.18573900 0.54961800
C -1.35595400 2.18696700 -2.24292500
C 1.05637200 2.48733100 -2.32473100
C 0.51540900 3.11940900 1.27004600

H	-0.94259400	1.53550000	1.07113400
C	2.81478800	2.49571500	0.82081900
H	3.12277500	0.42106800	0.26017500
C	-1.52380800	3.56547200	-2.13676500
H	-2.21035800	1.52371400	-2.26413500
C	0.87888000	3.86873000	-2.21657100
H	2.04466000	2.04844000	-2.38793600
C	1.86977500	3.45917900	1.17779800
H	-0.21802300	3.87300600	1.53962300
H	3.86487300	2.76203700	0.74741000
C	-0.40572000	4.40692600	-2.12015800
H	-2.52461200	3.98189800	-2.06829700
H	1.74649200	4.52097000	-2.19867400
H	2.18565600	4.47826200	1.38027500
H	-0.53758700	5.48131200	-2.03208700
Cu	-2.21214600	-2.40918800	-1.11817900
C	-4.61932800	0.32515000	-3.48109100
C	-4.96039200	1.24818100	-2.50095200
C	-4.62606200	0.98808700	-1.17332700
C	-3.92973400	-0.18260900	-0.87480500
C	-3.92016000	-0.83984600	-3.13260200
C	-3.63011500	-0.60960500	0.51297500
C	-3.94346100	0.14982500	1.63788700
C	-3.76234000	-0.42303700	2.89712800
H	-4.00235500	0.14362500	3.79103100
C	-3.30278600	-1.72895700	2.99318000
C	-2.95845400	-2.43423300	1.83074200
H	-4.89170600	0.48690800	-4.51749800
H	-5.49677000	2.15500000	-2.76052200
H	-3.18914900	-2.21109400	3.95761800
N	-3.56480700	-1.05323400	-1.84937100
N	-3.10602300	-1.85111500	0.62861100
C	-2.42388900	-3.83259600	1.89546600
H	-1.33224100	-3.79223200	1.91048900
H	-2.78207100	-4.33277400	2.79871800
H	-2.70591300	-4.38771800	0.99973600
C	-3.57293200	-1.87290900	-4.15944100
H	-2.49160700	-1.89625200	-4.29548500
H	-3.86686000	-2.86584900	-3.81235600
H	-4.06869700	-1.65118800	-5.10636900
H	-4.33658300	1.15385400	1.54555700
H	-4.91581300	1.67848400	-0.39232600
F	-2.99829500	-4.77918700	-1.14826100
F	-1.62110700	-3.30291000	-2.74578500
Cu	-1.19001400	-4.91813700	-1.76202200
C	2.76960300	-3.60924800	-2.84920900
C	3.45335100	-3.95452500	-1.69584900
C	2.83087600	-4.75690000	-0.73875100
C	1.52116900	-5.15828100	-0.96061900
C	1.44001500	-4.02599100	-3.02655500
C	0.77858300	-6.08796300	-0.08604400
C	1.34857700	-6.77685200	0.98408000
C	0.56913400	-7.69994300	1.67744700
H	0.99235800	-8.24402900	2.51559100
C	-0.73778100	-7.94929200	1.26837600
C	-1.26665100	-7.24682400	0.17873400
H	3.23788000	-3.01919300	-3.62684400
H	4.47492700	-3.62295300	-1.54317200
H	-1.34930000	-8.69429300	1.76448300
N	0.85706900	-4.75960400	-2.07113900
N	-0.50846000	-6.32064000	-0.43211300

C	-2.62800600	-7.52293200	-0.38286600
H	-3.30452400	-6.69793800	-0.15370800
H	-2.55057300	-7.55914900	-1.47272800
H	-3.02219400	-8.46351500	0.00511700
C	0.66912500	-3.68705800	-4.26543400
H	0.21328300	-2.69934400	-4.16076700
H	1.34860500	-3.69513300	-5.11928200
H	-0.12110800	-4.42106800	-4.41801300
H	2.38485100	-6.61876600	1.25064100
H	3.36389400	-5.06961100	0.14900600
F	-0.85280500	-3.57739700	-0.36385500
F	-1.51577600	-6.10144400	-3.15030400
N	2.15513700	-7.74929500	-3.89076000
S	3.45382000	-8.13086900	-2.89740900
S	2.33762900	-6.85426600	-5.30898700
C	3.48809800	-9.92556200	-2.91460800
O	4.69859700	-7.64205600	-3.51591800
O	3.15031900	-7.71157900	-1.52134300
C	3.21788000	-7.90636000	-6.46764400
O	0.96077400	-6.69716700	-5.80372900
O	3.15928000	-5.65938000	-5.08598300
C	3.47212800	-10.60967500	-4.13227900
C	3.60076800	-10.60101900	-1.69800700
C	2.48826900	-8.79369300	-7.26382000
C	4.61218600	-7.84690900	-6.52350500
C	3.55961700	-12.00133500	-4.12332700
H	3.37856100	-10.07229400	-5.06730700
C	3.69204600	-11.99293200	-1.70415700
H	3.59179600	-10.04210600	-0.76975900
C	3.17661900	-9.65922400	-8.11388500
H	1.40632400	-8.79869000	-7.22391500
C	5.28713400	-8.71841200	-7.37897400
H	5.14633000	-7.14621000	-5.89576400
C	3.66881800	-12.69177300	-2.91297600
H	3.53555600	-12.54410800	-5.06302900
H	3.75720900	-12.53084100	-0.76434900
C	4.57350600	-9.62690200	-8.16526000
H	2.62237100	-10.35323400	-8.73775100
H	6.37086400	-8.68721000	-7.42923300
H	3.72228200	-13.77572200	-2.91118100
H	5.10531200	-10.30546000	-8.82514700
C	0.26100000	-12.00829100	-2.03012800
C	0.17736100	-10.77229600	-2.77111500
C	0.40777500	-12.01327200	-0.62262100
C	0.23210600	-13.25508000	-2.69945000
C	0.01104500	-10.59325300	-4.16184200
S	0.30846900	-9.20183200	-1.97451800
C	0.52859900	-13.20677900	0.07870000
H	0.43811000	-11.07516600	-0.07644600
C	0.35493700	-14.44468500	-1.99208500
H	0.13165000	-13.29059400	-3.77807500
C	0.04070100	-9.27962000	-4.57123100
H	-0.13987200	-11.42028900	-4.84424500
C	0.35024500	-8.33879500	-3.51447700
C	0.50656400	-14.42975200	-0.60077500
H	0.64414000	-13.18526600	1.15793200
H	0.33756100	-15.38925700	-2.52702300
H	-0.10491800	-8.93591600	-5.58422200
H	-0.07408200	-7.33789300	-3.48752500
H	0.60598000	-15.36123400	-0.05231600

DTSN-N-4F (Quintet)
E(BS1) = -5954.501296
E(BS2) = -5958.021331
ZPE = 1.007797
H = 1.087554
G = 0.890155
<S^2> = 6.0324
330.8124i
N -0.46657700 -0.71311900 -1.01933700
S 0.51127000 -0.81829900 0.30990700
S 0.12374700 -0.12409900 -2.43636800
C 1.05847400 0.86087300 0.65184000
O 1.72602300 -1.63329400 0.11613700
O -0.38336600 -1.18988800 1.42478200
C -0.06249200 1.66344600 -2.32821200
O -0.82670000 -0.55052400 -3.48478900
O 1.55875900 -0.39578900 -2.66344100
C 0.10170000 1.81480400 1.01086200
C 2.40986500 1.18626200 0.55435200
C -1.35381600 2.18839600 -2.24270200
C 1.05856500 2.48938800 -2.32063300
C 0.51284300 3.11912400 1.27390400
H -0.94467400 1.53512600 1.07218800
C 2.81284500 2.49611700 0.82693400
H 3.12185500 0.42193600 0.26517700
C -1.52216300 3.56678700 -2.13586500
H -2.20802300 1.52495100 -2.26566500
C 0.88057400 3.87067000 -2.21179800
H 2.04704900 2.05076900 -2.38261800
C 1.86726100 3.45917800 1.18349000
H -0.22102900 3.87241100 1.54315200
H 3.86297100 2.76266300 0.75493000
C -0.40430100 4.40849400 -2.11696800
H -2.52317100 3.98292000 -2.06863800
H 1.74799900 4.52310600 -2.19212900
H 2.18273700 4.47816900 1.38706000
H -0.53655800 5.48278700 -2.02835300
Cu -2.21145400 -2.40742400 -1.12164300
C -4.61559300 0.32814100 -3.48625300
C -4.95809400 1.25055500 -2.50602700
C -4.62548900 0.98974400 -1.17811000
C -3.92938700 -0.18102400 -0.87934600
C -3.91674200 -0.83697100 -3.13751600
C -3.63152300 -0.60878300 0.50857400
C -3.94618100 0.15006000 1.63351000
C -3.76671200 -0.42354200 2.89265500
H -4.00776800 0.14264400 3.78657800
C -3.30749200 -1.72959200 2.98854600
C -2.96177000 -2.43426200 1.82615500
H -4.88662400 0.49046500 -4.52292500
H -5.49427000 2.15743900 -2.76578500
H -3.19518400 -2.21230200 3.95285400
N -3.56306700 -1.05104100 -1.85393000
N -3.10769200 -1.85041200 0.62418100
C -2.42759800 -3.83278800 1.89077400
H -1.33596100 -3.79270600 1.90763400
H -2.78736000 -4.33349000 2.79310800
H -2.70833900 -4.38725200 0.99423500
C -3.56807400 -1.86945900 -4.16444700
H -2.48652500 -1.89315100 -4.29859900
H -3.86303300 -2.86252300 -3.81856300
H -4.06209400 -1.64692900 -5.11209500
H -4.33903800 1.15419800 1.54123500
H -4.91641100 1.67962600 -0.39708600
F -2.99743000 -4.77747900 -1.15470600
F -1.61781900 -3.30008700 -2.74892400
Cu -1.18818800 -4.91572200 -1.76560600
C 2.77351300 -3.60793300 -2.84632700
C 3.45547800 -3.95414200 -1.69217400
C 2.83118000 -4.75635200 -0.73611500
C 1.52143200 -5.15661200 -0.95977500
C 1.44388100 -4.02369400 -3.02559200
C 0.77688500 -6.08607600 -0.08663800
C 1.34409500 -6.77442500 0.98526500
C 0.56309200 -7.69774400 1.67660400
H 0.98421300 -8.24149800 2.51602200
C -0.74258300 -7.94769800 1.26397300
C -1.26885200 -7.24534200 0.17300300
H 3.24328300 -3.01799700 -3.62316100
H 4.47713500 -3.62346100 -1.53809700
H -1.35523400 -8.69276700 1.75859100
N 0.85922400 -4.75702300 -2.07104800
N -0.50919400 -6.31916200 -0.43600500
C -2.62920200 -7.52110100 -0.39133200
H -3.30726200 -6.69820500 -0.15916700
H -2.55079900 -7.55281300 -1.48121500
H -3.02233900 -8.46381000 -0.00743200
C 0.67475000 -3.68429600 -4.26542800
H 0.21847000 -2.69676100 -4.16098500
H 1.35542100 -3.69194600 -5.11831700
H -0.11508000 -4.41850100 -4.41932900
H 2.37955900 -6.61592000 1.25471000
H 3.36288400 -5.06991800 0.15213100
F -0.85323700 -3.57630100 -0.36606500
F -1.51095600 -6.09783900 -3.15552400
N 2.15244100 -7.75061600 -3.88841100
S 3.44883900 -8.13221100 -2.89195000
S 2.33848300 -6.85592700 -5.30650500
C 3.48461500 -9.92687700 -2.90987500
O 4.69489400 -7.64226400 -3.50696900
O 3.14149600 -7.71384000 -1.51647300
C 3.22231100 -7.90764900 -6.46282500
O 0.96293400 -6.69948300 -5.80503200
O 3.15914400 -5.66078900 -5.08131600
C 3.47272400 -10.61048100 -4.12787100
C 3.59463000 -10.60275700 -1.69325500
C 2.49512700 -8.79451100 -7.26174800
C 4.61680400 -7.84827000 -6.51438600
C 3.56160500 -12.00205600 -4.11928000
H 3.38121100 -10.07282100 -5.06293200
C 3.68737300 -11.99456600 -1.69976200
H 3.58256000 -10.04427400 -0.76478200
C 3.18605000 -9.65979300 -8.10998200
H 1.41305900 -8.79927200 -7.22545500
C 5.29433500 -8.71953500 -7.36805300
H 5.14911400 -7.14784400 -5.88479100
C 3.66819500 -12.69290700 -2.90893900
H 3.54070100 -12.54443000 -5.05928800
H 3.75049600 -12.53281000 -0.76000500
C 4.58309100 -9.62767900 -8.15689100
H 2.63368100 -10.35343800 -8.73591700
H 6.37821900 -8.68840500 -7.41491500

H 3.72279900 -13.77680100 -2.90746100
H 5.11690000 -10.30607000 -8.81533000
C 0.25735800 -12.01274300 -2.03315000
C 0.17486900 -10.77702900 -2.77479400
C 0.40050900 -12.01720000 -0.62528500
C 0.23110200 -13.25977100 -2.70212000
C 0.01305200 -10.59848100 -4.16603500
S 0.30197400 -9.20608700 -1.97820100
C 0.52045600 -13.21040700 0.07672800
H 0.42881500 -11.07890000 -0.07932900
C 0.35303100 -14.44907000 -1.99407300
H 0.13338200 -13.29570700 -3.78098600
C 0.04297000 -9.28490000 -4.57574700
H -0.13472400 -11.42582700 -4.84875700
C 0.34756000 -8.34342200 -3.51827600
C 0.50110500 -14.43361700 -0.60238700
H 0.63323900 -13.18844700 1.15624600
H 0.33772400 -15.39381500 -2.52877700
H -0.09959600 -8.94168300 -5.58933400
H -0.07832800 -7.34287800 -3.49272600
H 0.59984500 -15.36485300 -0.05338500

D7-N-4F (Quintet)
E(BS1) = -5954.510308
E(BS2) = -5958.029236
ZPE = 1.009178
H = 1.089112
G = 0.890516
$\langle S^2 \rangle$ = 6.0389
N -0.39779100 -0.78211500 -0.99364100
S 0.52143500 -0.88320300 0.37808700
S 0.26075800 -0.15254800 -2.36266200
C 1.01480600 0.80419300 0.75869400
O 1.76045800 -1.66887100 0.22283300
O -0.40993800 -1.28883400 1.44956000
C -0.01266900 1.62398400 -2.25502900
O -0.59435200 -0.61066600 -3.47845700
O 1.71986400 -0.35715200 -2.48378800
C 0.02436300 1.72863400 1.10340300
C 2.35872500 1.16673400 0.69534900
C -1.32894000 2.08993700 -2.23869800
C 1.06746100 2.50013700 -2.18518900
C 0.39423700 3.04088500 1.38819200
H -1.01534600 1.42080200 1.13823300
C 2.71977000 2.48417600 0.98889900
H 3.09736600 0.42527100 0.41425700
C -1.56605000 3.45844400 -2.13648700
H -2.14910900 1.38916300 -2.31143500
C 0.82069900 3.87129900 -2.08174800
H 2.07699000 2.10802400 -2.19402900
C 1.74063700 3.41809000 1.33237300
H -0.36571100 3.77148700 1.64749000
H 3.76363300 2.77953900 0.94300700
C -0.49047600 4.34997400 -2.05431900
H -2.58732300 3.82779700 -2.12233000
H 1.65524700 4.56222800 -2.01347400
H 2.02365600 4.44318200 1.55231300
H -0.67617600 5.41663200 -1.96963800
Cu -2.15987700 -2.44019800 -1.17528400
C -4.51744100 0.33300400 -3.54975700
C -4.91093300 1.22296600 -2.55862600
C -4.61017800 0.93754300 -1.22856300
C -3.89728400 -0.22484100 -0.93623700
C -3.80307800 -0.82493400 -3.20839500
C -3.63059000 -0.67463900 0.45088600
C -3.96775800 0.06877500 1.57966000
C -3.81782400 -0.52431800 2.83365000
H -4.07562100 0.02945600 3.73066300
C -3.36649100 -1.83369200 2.91931600
C -2.99620200 -2.52191700 1.75449700
H -4.76046100 0.51505700 -4.59005600
H -5.46109200 2.12307200 -2.81253900
H -3.27833000 -2.33178500 3.87831400
N -3.48421900 -1.06461700 -1.91924100
N -3.11104700 -1.91872400 0.55846300
C -2.46978400 -3.92356300 1.81146700
H -1.37858500 -3.88883800 1.85110200
H -2.84981400 -4.43350000 2.70023400
H -2.73549000 -4.46618700 0.90323200
C -3.39907500 -1.82070900 -4.25094300
H -2.31208000 -1.83805900 -4.33159100
H -3.70670800 -2.82574500 -3.95454500
H -3.84539700 -1.56650200 -5.21404900
H -4.35322000 1.07636300 1.49384700
H -4.94049700 1.60057200 -0.44008300
F -2.99247000 -4.87019900 -1.24054000
F -1.59675500 -3.36965400 -2.79204800
Cu -1.16887900 -4.99163300 -1.80494100
C 2.73989400 -3.55551900 -2.86103900
C 3.43024900 -3.88972800 -1.70825800
C 2.83713000 -4.73516100 -0.76992200
C 1.54376400 -5.18328400 -1.00337800
C 1.43412200 -4.03315000 -3.05806900
C 0.82710500 -6.13142100 -0.12680500
C 1.44728300 -6.82799400 0.90989000
C 0.70497700 -7.75789300 1.63268200
H 1.16655600 -8.30669800 2.44712700
C -0.61858200 -8.00010700 1.28310800
C -1.19735400 -7.29730000 0.21761800
H 3.18435200 -2.92720700 -3.62266500
H 4.43286400 -3.51166400 -1.53922400
H -1.20934600 -8.74359800 1.80584000
N 0.87259300 -4.80032100 -2.11449600
N -0.47325000 -6.36518800 -0.42728700
C -2.57176600 -7.61944300 -0.28261400
H -3.09380700 -6.70495000 -0.56596700
H -2.46993000 -8.23820700 -1.18224000
H -3.13574700 -8.18032500 0.46497200
C 0.66918000 -3.72324900 -4.30816500
H 0.19176400 -2.74505800 -4.21455700
H 1.35940200 -3.71884100 -5.15359600
H -0.10082400 -4.47767400 -4.46388500
H 2.49384900 -6.66743400 1.13051600
H 3.37445800 -5.02933100 0.12149500
F -0.85261800 -3.64081500 -0.40723200
F -1.43931400 -6.16164600 -3.20945900
N 2.13983400 -7.82748200 -3.99170300
S 3.51724800 -8.22395800 -3.03844500
S 2.33605200 -6.84394600 -5.41377600
C 3.51564900 -10.01121900 -3.02066900
O 4.67116400 -7.76689100 -3.81985600
O 3.34594500 -7.72301200 -1.67253300

C 3.24644100 -7.84606600 -6.58808000
O 0.96371600 -6.69287200 -5.90942700
O 3.13727100 -5.66500300 -5.08973800
C 3.53536800 -10.69550400 -4.23772600
C 3.54704900 -10.67598400 -1.79462700
C 2.54801300 -8.78170000 -7.35689700
C 4.62342400 -7.65995200 -6.72419300
C 3.57233600 -12.08888100 -4.21713100
H 3.51028500 -10.15700200 -5.17784100
C 3.58864800 -12.06954800 -1.79144600
H 3.50950800 -10.10931500 -0.87215800
C 3.25802900 -9.57282100 -8.25914600
H 1.47467000 -8.88133100 -7.26233000
C 5.31841500 -8.45521500 -7.63529000
H 5.12985600 -6.92050200 -6.11866400
C 3.59668500 -12.77305700 -2.99811200
H 3.57609200 -12.63788700 -5.15327000
H 3.58848600 -12.60375200 -0.84742500
C 4.64072400 -9.41417400 -8.39270400
H 2.73008200 -10.30526200 -8.86104900
H 6.38955100 -8.32532300 -7.75051800
H 3.61265200 -13.85829500 -2.98875300
H 5.18899000 -10.03374000 -9.09558200
C 0.15453500 -11.79798500 -1.81923600
C 0.20073400 -10.63594900 -2.66817200
C 0.32244200 -11.69281100 -0.41584100
C -0.02224700 -13.09437700 -2.36399000
C 0.03302800 -10.56296100 -4.07465900
S 0.51920600 -9.02604400 -2.00828700
C 0.32231200 -12.82298300 0.39395300
H 0.46435200 -10.71704600 0.03971200
C -0.01941200 -14.21890200 -1.54788800
H -0.14293600 -13.21954300 -3.43439700
C 0.23021900 -9.33009100 -4.62260600
H -0.24200900 -11.42795200 -4.66740200
C 0.70035600 -8.26656600 -3.67253600
C 0.15491900 -14.09517500 -0.16371700
H 0.45661200 -12.71232900 1.46611900
H -0.14929900 -15.20119700 -1.99288900
H 0.09217300 -9.08723500 -5.66585200
H 0.09136700 -7.35921400 -3.67147600
H 0.15966500 -14.97701900 0.46940500

D8-N-3F (Constrained, Triplet)
E(BS1) = -5954.565310
E(BS2) = -5958.079056
ZPE = 1.009974
H = 1.089926
G = 0.891241
$\langle S^2 \rangle$ = 2.0039
N -0.44996700 -1.37225200 -0.57789400
S -0.27400200 -1.69433400 1.03264100
S 0.76995300 -0.63702400 -1.40251000
C -0.10447300 -0.08920200 1.82922000
O 0.94298900 -2.46111600 1.36755900
O -1.56548700 -2.24498700 1.48216500
C 0.52665400 1.13239300 -1.15799100
O 0.48677900 -0.90228500 -2.82870900
O 2.12412100 -0.94384900 -0.89875800
C -1.16133400 0.82090500 1.73506000
C 1.06485500 0.22327500 2.51976600
C -0.63130300 1.72854400 -1.65993000
C 1.47611500 1.87533700 -0.45898500
C -1.03720200 2.06640900 2.34580100
H -2.05672600 0.55639400 1.18280700
C 1.17625700 1.47235900 3.13574000
H 1.86868200 -0.50275700 2.55881600
C -0.84359700 3.08961600 -1.45433000
H -1.34988400 1.13443100 -2.20432900
C 1.25479800 3.23922100 -0.25490700
H 2.36058600 1.38421400 -0.07212000
C 0.12921200 2.39129300 3.04750900
H -1.84585100 2.78692700 2.27052200
H 2.08267700 1.72779900 3.67634300
C 0.09861000 3.84585800 -0.74949300
H -1.74326800 3.55721700 -1.84351800
H 1.98374500 3.82360000 0.29804100
H 0.22175100 3.36475700 3.51990600
H -0.07007400 4.90602300 -0.58498000
Cu -1.98343900 -2.67594600 -1.88636200
C -3.14519600 0.86883500 -4.19646400
C -3.93012900 1.52818400 -3.26296900
C -4.19496900 0.90688800 -2.04410500
C -3.65094300 -0.35261700 -1.79318700
C -2.60206400 -0.39186200 -3.89592500
C -3.99846600 -1.12682000 -0.57278100
C -4.72996200 -0.58475100 0.48370000
C -5.14121000 -1.43518500 1.50964200
H -5.71827600 -1.04430000 2.34180100
C -4.82309600 -2.78346800 1.44291100
C -4.03189900 -3.26493900 0.38712100
H -2.93483900 1.31167900 -5.16347300
H -4.34803600 2.50575200 -3.48072500
H -5.15580300 -3.47252400 2.21144700
N -2.84632000 -0.97006400 -2.69952400
N -3.62768100 -2.42576000 -0.58356000
C -3.61289700 -4.70360900 0.34573900
H -2.64270700 -4.79724000 0.84093000
H -4.34364400 -5.31901500 0.87911700
H -3.46200600 -5.07474500 -0.67064900
C -1.73767300 -1.08465100 -4.90637400
H -0.72513500 -0.67588800 -4.84608100
H -1.67132000 -2.15079900 -4.69333800
H -2.12664300 -0.90298900 -5.91239200
H -4.98731600 0.46614700 0.51135400
H -4.83974000 1.38689400 -1.32034100
F -2.64131600 -6.10123400 -2.38997900
F -1.47353500 -3.59858100 -3.46698900
Cu -0.86441700 -5.50714200 -2.36908500
C 3.24006200 -3.89986300 -2.48247700
C 3.70281500 -4.14991400 -1.19690300
C 2.84681800 -4.74542500 -0.27333900
C 1.55357600 -5.08445100 -0.67393800
C 1.92668200 -4.24879300 -2.82778500
C 0.63339600 -5.80571100 0.24653800
C 0.63577200 -5.57797500 1.62595600
C -0.24552300 -6.31694200 2.41180200
H -0.27930900 -6.16242600 3.48618400
C -1.09905200 -7.23697900 1.80564500
C -1.05993400 -7.38809800 0.41349600
H 3.87652600 -3.43120900 -3.22453100
H 4.71785100 -3.88806900 -0.91511800

H -1.80237500 -7.81777800 2.39333600
N 1.11751500 -4.84777200 -1.93136900
N -0.19100900 -6.69047200 -0.33533700
C -1.99641500 -8.28921800 -0.34012600
H -2.46180400 -7.68951100 -1.13273600
H -1.44001200 -9.10373900 -0.81693800
H -2.75499800 -8.72556400 0.31483900
C 1.36302900 -3.95821800 -4.18396900
H 0.62120900 -3.16163600 -4.08460400
H 2.14901000 -3.66009000 -4.88152500
H 0.83676000 -4.83606200 -4.56076400
H 1.25937900 -4.80370400 2.05215800
H 3.17824200 -4.96385300 0.73466300
F -1.28184800 -4.14558100 -0.95415500
F -0.32649100 -6.63182400 -3.81815900
N 2.90424400 -7.60578100 -5.33285400
S 4.53143300 -8.23239900 -5.26472500
S 2.31243100 -6.81504900 -6.79322800
C 4.25000200 -9.95162300 -4.88750700
O 5.07505400 -8.09474000 -6.61393000
O 5.19977800 -7.59811300 -4.12780900
C 2.20210300 -8.14900000 -7.97539300
O 0.95720600 -6.40122600 -6.44029500
O 3.32454300 -5.85011600 -7.20606000
C 3.70430100 -10.77207600 -5.88189400
C 4.53897600 -10.42017500 -3.60543000
C 1.06694000 -8.96590600 -7.93449700
C 3.24262100 -8.36291100 -8.88019600
C 3.41655600 -12.09705200 -5.56127800
H 3.50701900 -10.38378700 -6.87518700
C 4.24339600 -11.74949900 -3.30278100
H 4.96600000 -9.75312200 -2.86616900
C 0.99880200 -10.05467200 -8.80218700
H 0.25143900 -8.74194200 -7.25578100
C 3.15392100 -9.45407800 -9.74588300
H 4.10042500 -7.70382400 -8.88684500
C 3.67755400 -12.57999600 -4.27423500
H 2.98689600 -12.74920000 -6.31424900
H 4.45252600 -12.13313900 -2.30981000
C 2.04396800 -10.30225200 -9.69834700
H 0.12809700 -10.70161300 -8.78387200
H 3.95419200 -9.64109100 -10.45414700
H 3.44352000 -13.61132600 -4.03010200
H 1.98711500 -11.15329600 -10.36962700
C 1.72199000 -9.35249300 -0.57014500
C 1.77506900 -9.03765600 -1.98164200
C 2.41696700 -8.56557200 0.37776900
C 0.94226700 -10.43867600 -0.11074300
C 1.20474300 -9.83850100 -3.03085500
S 2.52763700 -7.62633100 -2.56618200
C 2.32076700 -8.84353300 1.73169300
H 3.02167900 -7.72537900 0.05281500
C 0.85053800 -10.71207600 1.24844600
H 0.38756600 -11.05465800 -0.80765000
C 1.36772700 -9.31035600 -4.26520900
H 0.71595800 -10.78363900 -2.84256900
C 1.94939800 -7.94455900 -4.29445600
C 1.53379600 -9.91515900 2.17138500
H 2.84702200 -8.22209200 2.44759600
H 0.23886100 -11.54028300 1.58957100
H 1.02447600 -9.77382900 -5.18156700
H 1.08429400 -7.27875500 -4.31959700
H 1.45305400 -10.12611200 3.23298100

D9-N-3F (Triplet)
E(BS1) = -5954.620062
E(BS2) = -5958.131933
ZPE = 1.008872
H = 1.089489
G = 0.893722
$\langle S^2 \rangle$ = 2.0038
N -0.57330900 -1.34191300 -0.24731400
S -0.92480100 -1.32115400 1.38395900
S 0.91978300 -0.86096800 -0.76326000
C -0.64065800 0.38274200 1.87731900
O -0.03370200 -2.16315700 2.20213700
O -2.37605600 -1.55852700 1.47326400
C 0.78389800 0.91638600 -1.02219500
O 1.07457200 -1.47439900 -2.10013800
O 1.99783500 -1.06162800 0.22387500
C -1.49484300 1.37123200 1.38092300
C 0.43194200 0.69344300 2.71099000
C -0.23813700 1.40675200 -1.83755200
C 1.70446400 1.77244900 -0.42142300
C -1.26237100 2.69968800 1.72944700
H -2.31501400 1.09835600 0.72542800
C 0.65112300 2.02796700 3.05970000
H 1.07917000 -0.10017800 3.06468600
C -0.33670600 2.77848000 -2.05568600
H -0.95182300 0.72676100 -2.27846200
C 1.59532500 3.14757000 -0.64283400
H 2.47762500 1.36517900 0.21888600
C -0.19140200 3.02705300 2.56818400
H -1.90989600 3.48033700 1.34267000
H 1.48283300 2.28578500 3.70823900
C 0.57994200 3.65035100 -1.45820800
H -1.13109400 3.16507900 -2.68713900
H 2.30160500 3.82379600 -0.17108100
H -0.01217000 4.06465800 2.83373900
H 0.49818100 4.72032400 -1.62467100
Cu -1.60459200 -2.84582100 -1.49225600
C -1.95833700 0.01703900 -4.78367400
C -2.97652600 0.82757800 -4.30427000
C -3.64427600 0.45551100 -3.13863500
C -3.24318900 -0.69870100 -2.46566300
C -1.58280600 -1.13152100 -4.06958100
C -3.99612400 -1.23068800 -1.29945000
C -4.96797500 -0.49228400 -0.62268200
C -5.70854500 -1.13285400 0.36989600
H -6.47744000 -0.58995000 0.91083600
C -5.46024100 -2.47000600 0.65046000
C -4.43109200 -3.14233500 -0.02715900
H -1.44361200 0.25201100 -5.70864100
H -3.27213300 1.72253300 -4.84222000
H -6.03672300 -2.99681400 1.40330400
N -2.19498400 -1.44517800 -2.90833300
N -3.72416700 -2.51233900 -0.97991100
C -4.07251300 -4.55952600 0.30480600
H -3.21949200 -4.54881100 0.98804700
H -4.91522900 -5.06829800 0.78123000
H -3.73545100 -5.11467800 -0.57445600
C -0.50185000 -2.02275700 -4.60299700

H 0.47549300 -1.57845100 -4.39956900
H -0.54490400 -3.00038800 -4.12661000
H -0.61367600 -2.12736000 -5.68630200
H -5.15048600 0.54940000 -0.85562900
H -4.48538100 1.03606300 -2.78366400
F -2.57960200 -6.40267800 -1.83831600
F -1.10805500 -4.32709800 -2.73433400
Cu -0.87514800 -5.83149900 -1.43456800
C 3.10159200 -4.06588200 -1.11603300
C 3.30012400 -4.13864300 0.25281200
C 2.35686900 -4.79669500 1.03946300
C 1.22792600 -5.33453700 0.42820900
C 1.95242200 -4.63396700 -1.68440200
C 0.20234200 -6.08899300 1.18734100
C 0.12586100 -6.06272700 2.58072500
C -0.83059400 -6.85237500 3.21193300
H -0.92067600 -6.83960400 4.29351400
C -1.65410100 -7.66809700 2.44398200
C -1.54318400 -7.64124500 1.04801000
H 3.81550400 -3.56698100 -1.76144500
H 4.18094100 -3.69882100 0.70846900
H -2.38747000 -8.31828700 2.90817700
N 1.03976300 -5.24635300 -0.91148200
N -0.64778500 -6.83464300 0.44756300
C -2.38414400 -8.51565200 0.16646900
H -2.75994900 -7.91219100 -0.66541500
H -1.75999400 -9.31303400 -0.25527600
H -3.20129700 -8.97625600 0.72606800
C 1.74490200 -4.55649300 -3.16560500
H 1.51939200 -3.52302300 -3.43208500
H 2.65633400 -4.85184000 -3.69221900
H 0.91830500 -5.17827800 -3.49248800
H 0.77722000 -5.42197100 3.16004400
H 2.51052900 -4.90110900 2.10500000
F -1.31950900 -4.06495900 -0.13066100
F -0.10936000 -7.59696500 -2.79298000
N 2.98259900 -7.92231800 -5.24625400
S 4.52146000 -8.26823600 -5.99247800
S 1.77620300 -6.96005400 -6.06504200
C 4.37341000 -9.99375600 -6.40787700
O 4.55965400 -7.46234800 -7.21304200
O 5.52331700 -8.11619200 -4.93863900
C 1.43864100 -7.83489700 -7.57578000
O 0.58357600 -7.09259700 -5.20239200
O 2.31525600 -5.63517800 -6.35135100
C 3.84310200 -10.34260900 -7.65255600
C 4.73945800 -10.95027100 -5.45660800
C 0.68074200 -9.00771500 -7.50642600
C 1.96379800 -7.34800400 -8.77350400
C 3.67116500 -11.69473100 -7.94495500
H 3.57751700 -9.57853000 -8.37295500
C 4.56063000 -12.29789700 -5.76763600
H 5.15513000 -10.64290900 -4.50427700
C 0.45462100 -9.71909800 -8.68292200
H 0.27629500 -9.34874800 -6.56008700
C 1.72404500 -8.07294700 -9.94119200
H 2.55578600 -6.44162000 -8.77878700
C 4.02478900 -12.66732500 -7.00505600
H 3.26022500 -11.98472400 -8.90633400
H 4.83884800 -13.05666600 -5.04369200
C 0.97787400 -9.25460800 -9.89406900
H -0.12833200 -10.63339400 -8.65333300
H 2.12348500 -7.71605100 -10.88455700
H 3.88582300 -13.71817300 -7.23915600
H 0.80115000 -9.81559000 -10.80626800
C 2.40431300 -8.92256400 -0.10111800
C 2.43582100 -8.98810100 -1.56697400
C 3.34630000 -8.17174900 0.62434800
C 1.40049700 -9.60793000 0.60852600
C 1.93600500 -9.98610000 -2.38436300
S 3.10923400 -7.69144500 -2.51282800
C 3.28592200 -8.10810200 2.01499900
H 4.13346000 -7.63821000 0.09985900
C 1.34763500 -9.54882100 1.99857500
H 0.64208200 -10.16119000 0.06478300
C 2.08067300 -9.71481800 -3.76545100
H 1.48657600 -10.89209900 -1.99703600
C 2.69623900 -8.50642600 -4.00365000
C 2.28762700 -8.79604700 2.70808000
H 4.01906900 -7.51789500 2.55630100
H 0.55525800 -10.06908400 2.52720200
H 1.75323100 -10.37240200 -4.56177900
H 0.19383500 -7.39463000 -3.67288300
H 2.23541000 -8.73866600 3.79087500

1’

E(BS1) = -2398.742905
E(BS2) = -2399.112425
ZPE = 0.346208
H = 0.373233
G = 0.288750

C 3.60691800 -3.76505800 -1.42400300
C 2.44866200 -4.32783800 -1.92409700
C 1.40723800 -3.39257900 -2.14435800
C 1.77115100 -2.10879400 -1.81979300
S 3.42307600 -2.03561800 -1.23229300
H 2.34396100 -5.39171000 -2.09785400
H 0.41931400 -3.64112200 -2.51194100
C 4.86343100 -4.44845200 -1.08975900
C 5.72684200 -3.95762500 -0.09367600
C 5.22238100 -5.62819800 -1.76825900
C 6.91013900 -4.62610500 0.21436300
H 5.46061600 -3.05992000 0.45666400
C 6.40105600 -6.29923000 -1.45034100
H 4.58476000 -6.00689700 -2.56056700
C 7.25119000 -5.80112400 -0.45947200
H 7.56176200 -4.23267500 0.98859500
H 6.66199900 -7.20679900 -1.98631700
H 8.17193800 -6.32255500 -0.21675100
N 0.98489600 -0.95255900 -1.89715200
S -0.14247200 -0.59020000 -0.60033200
S 0.95913000 -0.06517600 -3.39040400
C 0.60474200 0.80613200 0.21764300
O -0.06236700 -1.75747200 0.27812200
O -1.41448200 -0.18994100 -1.20438500
C -0.15186700 -1.00177800 -4.42352100
O 2.31468000 -0.17997100 -3.92977900
O 0.37676600 1.24117800 -3.08260400
C 0.18799700 2.09576300 -0.11432300
C 1.60824500 0.56190000 1.15929300
C -1.52358500 -0.75061100 -4.33998800
C 0.37797500 -1.99435800 -5.25176600

C 0.80118900 3.17370000 0.52329900
H -0.58476800 2.24220500 -0.85804300
C 2.21272500 1.65240800 1.78232100
H 1.89826500 -0.45405900 1.40160100
C -2.38580800 -1.52147100 -5.11767800
H -1.89685500 0.01820300 -3.67502700
C -0.50131600 -2.75733900 -6.01994400
H 1.44865600 -2.15476100 -5.29530000
C 1.81036900 2.95290100 1.46439000
H 0.49076100 4.18518300 0.28299400
H 2.99297600 1.48555000 2.51750900
C -1.87687300 -2.52188600 -5.95150500
H -3.45471800 -1.34107900 -5.07179700
H -0.11067400 -3.53183300 -6.67164200
H 2.28451200 3.79773800 1.95385700
H -2.55588500 -3.11919800 -6.55180500

HF
E(BS1) = -100.432113
E(BS2) = -100.4877494
ZPE = 0.009233
H = 0.012538
G = -0.004167
F -0.33936500 0.64131700 0.00000000
H -1.26708200 0.64131700 0.00000000

Benzene
E(BS1) = -232.265455
E(BS2) = -232.318802
ZPE = 0.100593
H = 0.105928
G = 0.076145
C -0.08156700 0.14130100 0.00005900
C 1.31594100 0.14123100 0.00050500
C 2.01475900 1.35145900 -0.00004700
C 1.31606200 2.56175900 -0.00104400
C -0.08144500 2.56182800 -0.00149000
C -0.78026400 1.35159900 -0.00093800
H -0.62479100 -0.79942900 0.00048900
H 1.85907100 -0.79955300 0.00128100
H 3.10106800 1.35140000 0.00029700
H 1.85927900 3.50249300 -0.00147400
H -0.62456800 3.50261600 -0.00226600
H -1.86657200 1.35165000 -0.00128300

D5-N-4F (Benzene, Triplet)
E(BS1) = -5402.655816
E(BS2) = -5406.109608
ZPE = 0.960141
H = 1.036911
G = 0.844299
<S^2> = 3.0051
N -0.17270900 -1.25598600 -1.27886500
S 0.44016600 -0.29289900 -0.07813700
S 0.10345400 -0.89218000 -2.85747700
C -0.15585100 1.36724600 -0.42908900
O 1.91344000 -0.22246800 -0.05606000
O -0.24315300 -0.72789000 1.15650300
C -1.20666300 0.25196000 -3.32143500
O -0.16888400 -2.13903000 -3.60474500
O 1.38502200 -0.20180000 -3.11534500
C -1.52359900 1.62792500 -0.30706700
C 0.74225500 2.35536700 -0.82841100
C -2.53223700 -0.17237000 -3.20711900
C -0.89068200 1.52602500 -3.78607300
C -1.99597700 2.90641800 -0.59417800
H -2.19974700 0.83694700 -0.00091200
C 0.25865400 3.63582500 -1.10791700
H 1.79468300 2.11341400 -0.91863700
C -3.55890100 0.70111700 -3.55690700
H -2.75273900 -1.16812700 -2.84813300
C -1.92696600 2.39667200 -4.13237800
H 0.14707600 1.82913100 -3.85283700
C -1.10565000 3.90942700 -0.99277800
H -3.05727400 3.11997000 -0.51423800
H 0.94712000 4.41559300 -1.41926000
C -3.25668300 1.98742000 -4.01807500
H -4.59236000 0.37875600 -3.46947600
H -1.69211300 3.39693100 -4.48290500
H -1.47900900 4.90402900 -1.21791900
H -4.05857100 2.66929100 -4.28523800
Cu -0.45176100 -3.53323100 -0.67710300
C -3.70794900 -3.82749600 -3.54270500
C -4.70475500 -3.17237000 -2.83775600
C -4.46306800 -2.79247800 -1.51769500
C -3.21635800 -3.05927200 -0.95671600
C -2.46578600 -4.08145500 -2.93719100
C -2.90715500 -2.77451600 0.46511200
C -3.78967600 -2.12059000 1.32252000
C -3.45876000 -2.03038700 2.67408900
H -4.12880000 -1.52963700 3.36549300
C -2.28205900 -2.60902500 3.12701900
C -1.40522300 -3.21692000 2.21607100
H -3.86864700 -4.16002700 -4.56208700
H -5.66645800 -2.96896800 -3.29706000
H -2.01799700 -2.58590500 4.17835700
N -2.23398800 -3.67214000 -1.67374000
N -1.72705000 -3.26425100 0.91009700
C -0.11191700 -3.81707700 2.67890600
H 0.67689500 -3.06457500 2.60200700
H -0.20122100 -4.13750600 3.72023100
H 0.18335300 -4.64943600 2.03863400
C -1.41906800 -4.83872600 -3.69839100
H -1.09696500 -4.24562600 -4.55796200
H -0.54631000 -5.04555900 -3.08501200
H -1.85174700 -5.77338900 -4.07059300
H -4.71845400 -1.70016700 0.95980900
H -5.24038800 -2.31111900 -0.93980400
F 0.79873400 -5.88319400 0.39783400
F 0.80335400 -4.53755500 -1.80142700
Cu 2.14313500 -4.80565400 -0.39319900
C 4.21872200 -1.84879800 -2.79804500
C 4.87156800 -1.13015000 -1.80981200
C 4.81009200 -1.56913600 -0.48816200
C 4.05835900 -2.70303700 -0.19740900
C 3.49088800 -3.00113400 -2.46277800
C 3.97294800 -3.29463500 1.15486100
C 4.53115700 -2.69930600 2.28539200
C 4.46053800 -3.37040200 3.50376400
H 4.87469300 -2.91682700 4.39839400
C 3.87813900 -4.62957800 3.55788200
C 3.33315600 -5.19638200 2.39758700

H 4.26287700 -1.54282200 -3.83644500
H 5.43967100 -0.24023700 -2.06042700
H 3.84324600 -5.19228300 4.48367700
N 3.41303100 -3.37259900 -1.17744600
N 3.35687300 -4.49612700 1.25119100
C 2.79780500 -6.59380000 2.38551000
H 1.90108100 -6.64681000 1.76684200
H 3.56204300 -7.23588700 1.93668200
H 2.59660300 -6.94196200 3.40068800
C 2.82292900 -3.82825300 -3.51854200
H 1.78789600 -3.50172500 -3.64079000
H 3.35791000 -3.71053500 -4.46344600
H 2.82252700 -4.87654900 -3.22314500
H 5.00338400 -1.72812200 2.22098300
H 5.33957900 -1.03656800 0.29052700
F 1.15505500 -3.26274900 0.32363900
F 3.07584500 -6.20205000 -1.16605800
N 5.60199500 -6.90853500 -1.17380900
S 6.63164400 -6.29164700 0.06945700
S 5.67070900 -6.25936000 -2.77758500
C 7.27630600 -6.90343800 -3.20443500
O 4.64904000 -6.99187500 -3.52719500
O 5.70873400 -4.79576900 -2.79586600
C 7.73726500 -7.68606200 0.11547500
O 7.37608600 -5.08110000 -0.29967300
O 5.78397400 -6.28208300 1.26616600
C 7.41568000 -8.28622800 -3.38399000
C 8.67991900 -8.79416700 -3.66958600
C 9.77319000 -7.92807900 -3.77837700
C 8.35900200 -6.02436500 -3.29558400
C 9.61397300 -6.54965300 -3.59748400
H 6.55636200 -8.94016700 -3.28973700
H 8.81356300 -9.86232500 -3.80365500
H 10.75689700 -8.33044800 -3.99917700
H 8.21030000 -4.96451500 -3.12869900
H 10.46803800 -5.88622300 -3.68534200
C 9.08554800 -7.49326500 -0.19781100
C 9.93563400 -8.59696200 -0.17173700
C 9.43281900 -9.86250500 0.14833800
C 7.21342100 -8.94456600 0.43916800
C 8.07796900 -10.03575500 0.45112900
H 9.44583900 -6.50698500 -0.46302900
H 10.98631600 -8.47098100 -0.41110800
H 10.09955400 -10.71931500 0.15471900
H 6.15887200 -9.06853700 0.65546200
H 7.68616100 -11.01932800 0.68625400
C 3.59159400 -9.94918000 0.88482100
C 3.57747700 -9.23532600 -0.31661700
C 4.20841300 -9.77109400 -1.44563400
C 4.85525800 -11.00592400 -1.37155400
C 4.87951900 -11.71294600 -0.16358500
C 4.24598600 -11.18508400 0.96418000
H 3.10055200 -9.54218600 1.76415400
H 3.12301200 -8.25173300 -0.38454500
H 4.20802000 -9.20262800 -2.37014500
H 5.34895700 -11.41626000 -2.24868000
H 5.38913900 -12.67080900 -0.10313400
H 4.26156000 -11.73269900 1.90255800

D5-N-4F (Benzene, Quintet)
E(BS1) = -5402.656333
E(BS2) = -5406.108149
ZPE = 0.959761
H = 1.036636
G = 0.843878
<S^2> = 6.0158
N -0.22390200 -1.20485500 -1.30769900
S 0.35605300 -0.12866400 -0.19349400
S 0.02575400 -0.95407000 -2.91288700
C -0.35081800 1.46528700 -0.63604400
O 1.82255500 0.03842200 -0.20933700
O -0.27040700 -0.52066400 1.08575500
C -1.32772600 0.10759200 -3.44683400
O -0.21139900 -2.25712600 -3.56901600
O 1.28118000 -0.24189000 -3.23600500
C -1.73140500 1.64382900 -0.51211600
C 0.47590500 2.48329500 -1.10753300
C -2.63536000 -0.36018100 -3.29917400
C -1.06358600 1.35906100 -3.99709700
C -2.28929600 2.86921200 -0.86879000
H -2.35139500 0.83059400 -0.15055500
C -0.09267000 3.71086600 -1.45599100
H 1.54050400 2.30348100 -1.19926500
C -3.69648900 0.44543500 -3.70464700
H -2.81408200 -1.33722900 -2.87127400
C -2.13410800 2.16180900 -4.39906600
H -0.03884400 1.69783800 -4.08915300
C -1.47061100 3.90209100 -1.33844300
H -3.36146300 3.01836800 -0.78709900
H 0.54026200 4.51295600 -1.82349600
C -3.44626900 1.70829700 -4.25373100
H -4.71626600 0.08880900 -3.59224100
H -1.93983100 3.14445800 -4.81779000
H -1.91056100 4.85499100 -1.61728400
H -4.27489800 2.33759900 -4.56484500
Cu -0.28915200 -3.49199300 -0.54245300
C -3.57996200 -4.11702800 -3.32536500
C -4.59043000 -3.45292500 -2.64873900
C -4.34407200 -2.97453200 -1.36157000
C -3.08021300 -3.15862800 -0.80604300
C -2.32081400 -4.28389300 -2.72421400
C -2.75837700 -2.77245300 0.58860700
C -3.65325100 -2.11294700 1.42875800
C -3.29849300 -1.92911200 2.76474100
H -3.97694200 -1.42048500 3.44205800
C -2.08738500 -2.42804900 3.22346300
C -1.20454200 -3.04912700 2.32833800
H -3.74285800 -4.52186200 -4.31800400
H -5.56533600 -3.31554700 -3.10483000
H -1.80423000 -2.33477500 4.26591800
N -2.08720500 -3.78405400 -1.49564400
N -1.54855000 -3.18002500 1.03441400
C 0.11711000 -3.58620800 2.79014100
H 0.88694200 -2.82307900 2.65543100
H 0.06207800 -3.85975600 3.84688300
H 0.41158500 -4.44108100 2.18051600
C -1.25761400 -5.06110600 -3.44141300
H -1.06408300 -4.60157000 -4.41393500
H -0.32674000 -5.08137100 -2.88196800
H -1.61649700 -6.08201300 -3.61408900
H -4.60938200 -1.76002700 1.06505400
H -5.12913900 -2.47989800 -0.80555200

F 0.82088000 -5.59020600 0.36698600
F 1.03192300 -4.23897500 -1.78612600
Cu 2.29094600 -4.58555200 -0.33583900
C 4.43431900 -1.50532100 -2.46420400
C 5.02152900 -0.83672800 -1.40210600
C 4.94197600 -1.37749400 -0.11855800
C 4.23564700 -2.56198700 0.06189600
C 3.73808500 -2.70369200 -2.23866600
C 4.13618400 -3.26799800 1.35839600
C 4.73923800 -2.81039400 2.52905800
C 4.63701900 -3.58262200 3.68430000
H 5.08740200 -3.23727400 4.60918600
C 3.97816700 -4.80364900 3.63556800
C 3.39828000 -5.23544800 2.43461900
H 4.49741800 -1.12047900 -3.47525500
H 5.55452200 0.09424600 -1.56534100
H 3.91389400 -5.44155300 4.50957700
N 3.63991500 -3.17136500 -0.98727400
N 3.45859100 -4.44003100 1.35371100
C 2.77750900 -6.59155900 2.30516000
H 1.87129000 -6.53703900 1.70142100
H 3.49414300 -7.23595400 1.78677700
H 2.56664900 -7.01746700 3.28812200
C 3.12543800 -3.47038800 -3.37012100
H 2.10877600 -3.11479200 -3.55332400
H 3.72812300 -3.32752100 -4.27005800
H 3.08115800 -4.52832200 -3.11809700
H 5.27817800 -1.87232700 2.54384900
H 5.42062300 -0.88055700 0.71488800
F 1.33589400 -3.02654200 0.39369900
F 3.17761700 -5.99079100 -1.14203900
N 5.65636900 -6.85032400 -1.14305600
S 6.69379400 -6.43894400 0.17662800
S 5.82625100 -6.05854800 -2.67381800
C 7.40723300 -6.75417200 -3.11009700
O 4.79771300 -6.65674100 -3.52612000
O 5.94162100 -4.60422300 -2.54452900
C 7.65800000 -7.93459000 0.13515400
O 7.56466300 -5.28193000 -0.06660600
O 5.80856200 -6.44134700 1.34627800
C 7.46425300 -8.11270900 -3.44931600
C 8.70444900 -8.67155900 -3.74553300
C 9.85585000 -7.87795100 -3.70473500
C 8.54792300 -5.94890000 -3.05166600
C 9.77862100 -6.52263600 -3.36457900
H 6.55957600 -8.70960700 -3.46845700
H 8.77398900 -9.72276000 -4.00424000
H 10.82064300 -8.31946100 -3.93412100
H 8.46135800 -4.90850200 -2.76325300
H 10.67749500 -5.91550400 -3.33805100
C 9.02929300 -7.85114200 -0.12246600
C 9.76773000 -9.03188700 -0.16661200
C 9.13433700 -10.26348900 0.03074800
C 7.00422700 -9.15769000 0.33602400
C 7.75914300 -10.32615600 0.28016300
H 9.49375500 -6.88733900 -0.29071100
H 10.83375700 -8.99112600 -0.36427100
H 9.71490300 -11.17966300 -0.01692100
H 5.93536300 -9.19431900 0.51139500
H 7.26638600 -11.28200800 0.41998800
C 3.29300200 -9.88956900 0.53412800
C 3.41286700 -9.08507800 -0.60244700
C 4.06376100 -9.58446900 -1.73684100
C 4.59515200 -10.87524900 -1.73248400
C 4.48226700 -11.67572400 -0.58961500
C 3.83064900 -11.18290100 0.54372900
H 2.78784500 -9.50860200 1.41735800
H 3.04668500 -8.06312400 -0.61052200
H 4.16837400 -8.94716900 -2.60931700
H 5.10444600 -11.25802300 -2.61308100
H 4.90066500 -12.67864400 -0.58366100
H 3.74135000 -11.80261900 1.43182300

D6-N-4F (Benzene, Quintet)
E(BS1) = -5402.659268
E(BS2) = -5406.112991
ZPE = 0.960255
H = 1.036716
G = 0.846150
$\langle S^2 \rangle$ = 6.0174
N -0.12817700 -0.93478300 -1.23771000
S 0.92380000 -0.88852700 0.03573300
S 0.27601300 -0.25421800 -2.68003400
C 1.16981600 0.85461000 0.40488500
O 2.25219300 -1.46534300 -0.24843000
O 0.18107500 -1.44268300 1.18476400
C -0.18405100 1.48118900 -2.54805000
O -0.65232300 -0.83409400 -3.67341000
O 1.72058500 -0.28585800 -2.99100600
C 0.06524100 1.61259100 0.80549800
C 2.43913800 1.41872100 0.29586000
C -1.53994800 1.80319200 -2.46797100
C 0.79870800 2.46810400 -2.51699500
C 0.24216200 2.96284100 1.09619600
H -0.91327300 1.14954500 0.87725000
C 2.60681200 2.77325500 0.59577900
H 3.27069300 0.80102400 -0.02347600
C -1.91679600 3.13819000 -2.33974200
H -2.28177300 1.01854300 -2.51943000
C 0.41248900 3.80377900 -2.38652700
H 1.84282000 2.18714300 -2.57926200
C 1.51179600 3.54264200 0.99250000
H -0.60879000 3.56576100 1.39784600
H 3.59068900 3.22522800 0.51418400
C -0.93998600 4.13906500 -2.29543200
H -2.96989800 3.39648500 -2.27770100
H 1.17071700 4.57969300 -2.34895800
H 1.64413200 4.59660900 1.21808200
H -1.23437300 5.17921800 -2.19149300
Cu -1.77293600 -2.70611100 -1.17161600
C -4.65424200 -0.24217300 -3.30305100
C -5.00827700 0.64087300 -2.29119800
C -4.51987600 0.43175900 -1.00325300
C -3.66884300 -0.64905900 -0.77581600
C -3.79066500 -1.31338800 -3.02849100
C -3.20592500 -1.03371100 0.57900700
C -3.48157800 -0.29337300 1.72644900
C -3.14492600 -0.83977100 2.96542200
H -3.35134600 -0.28769400 3.87660200
C -2.57365300 -2.10321300 3.02090500
C -2.27069600 -2.78535900 1.83300000
H -5.04054200 -0.12091000 -4.30830200

H -5.67116300 1.47544300 -2.49464900
H -2.33874800 -2.56786300 3.97190200
N -3.29546200 -1.47779000 -1.78395300
N -2.56760300 -2.22440900 0.64768700
C -1.61242800 -4.13104800 1.85285700
H -0.53373800 -3.99145200 1.75190600
H -1.82823900 -4.64365100 2.79353100
H -1.93448100 -4.72722800 0.99744800
C -3.41576400 -2.29554600 -4.09437600
H -2.36431300 -2.16242200 -4.35198600
H -3.51707700 -3.31636000 -3.72259800
H -4.04013900 -2.15477100 -4.97858400
H -3.96082100 0.67522800 1.66754200
H -4.81555100 1.08673000 -0.19455300
F -2.39632100 -5.23151300 -1.09559000
F -1.33052900 -3.62201500 -2.83352500
Cu -0.66087900 -5.18362100 -1.89197200
C 2.88234100 -3.38368200 -3.49035700
C 3.72007200 -3.52864400 -2.39452300
C 3.35348000 -4.38086200 -1.35266500
C 2.11924200 -5.01459200 -1.41981200
C 1.64313800 -4.04498600 -3.51283300
C 1.63205700 -5.98874600 -0.42004200
C 2.42884000 -6.48656700 0.61013000
C 1.89491100 -7.43836600 1.47496900
H 2.49659800 -7.83386800 2.28651900
C 0.59552900 -7.89112200 1.27687600
C -0.16683700 -7.37739400 0.21849000
H 3.15442400 -2.75084700 -4.32644900
H 4.66388900 -2.99541900 -2.35180500
H 0.15894500 -8.64279400 1.92450400
N 1.29133600 -4.80030700 -2.46583500
N 0.36277100 -6.43550200 -0.58365700
C -1.54638300 -7.88968500 -0.07161300
H -2.17724900 -7.07987900 -0.44072400
H -1.48812100 -8.66504000 -0.84367800
H -1.98378000 -8.33912000 0.82233800
C 0.71423200 -3.92883300 -4.68376700
H 0.20789500 -2.96008000 -4.65172200
H 1.29293900 -4.00923800 -5.60755300
H -0.03361600 -4.71944400 -4.64227800
H 3.44978100 -6.14904000 0.72855500
H 4.01116500 -4.52732200 -0.50632900
F -0.29468800 -3.78617400 -0.55091500
F -1.03525200 -6.35645300 -3.25648400
N 3.48609700 -8.92563200 -4.83002900
S 3.75527500 -8.64954600 -3.17345400
S 3.62306400 -7.57470400 -5.89639400
C 2.96453800 -10.06486100 -2.43562800
O 5.21529500 -8.79247600 -3.00060900
O 3.11539500 -7.42704100 -2.66463900
C 5.23881100 -6.91085200 -5.54590700
O 3.63990200 -8.19468800 -7.22880000
O 2.61191700 -6.55285800 -5.59368000
C 3.34367200 -11.35407000 -2.81913600
C 2.07106600 -9.83699700 -1.38919100
C 6.35082600 -7.44505200 -6.20151000
C 5.34458600 -5.88508800 -4.60380800
C 2.80595000 -12.43874000 -2.12920300
H 4.03912200 -11.50233500 -3.63755700
C 1.55313400 -10.93280400 -0.69781100
H 1.79607000 -8.82427100 -1.12859400
C 7.60815100 -6.92220900 -5.90380300
H 6.22592000 -8.23846900 -6.92961300
C 6.60873300 -5.37459000 -4.31890600
H 4.45848500 -5.50979200 -4.10888400
C 1.91946300 -12.22825900 -1.06679200
H 3.08230100 -13.44797600 -2.41674700
H 0.86109100 -10.76841600 0.12181600
C 7.73569000 -5.89284800 -4.96490100
H 8.48693600 -7.31582600 -6.40418100
H 6.71326900 -4.57381700 -3.59383400
H 1.51208200 -13.07892600 -0.52921400
H 8.71879800 -5.49262900 -4.73683000
C 0.97835600 -9.48854300 -5.60130200
C 0.34714800 -8.63175100 -4.67528000
C -0.34609300 -9.17308700 -3.59781800
C -0.40123800 -10.56051800 -3.43041500
C 0.24477300 -11.41786900 -4.34002200
C 0.92795000 -10.88525900 -5.42341100
H 1.42802700 -9.08617100 -6.50068900
H 0.40333300 -7.55651000 -4.78627200
H -0.83702400 -8.49221300 -2.91457300
H -0.93359400 -10.98305800 -2.58378100
H 0.20666400 -12.49185300 -4.18939700
H 1.42348200 -11.53532000 -6.13734400

DTSN-N-4F (Benzene, Quintet)
E(BS1) = -5402.650907
E(BS2) = -5406.104445
ZPE = 0.96038
H = 1.035519
G = 0.849224
$\langle S^2 \rangle$ = 6.0385
335.0240i
N -0.15049700 -0.91988600 -1.22832200
S 0.91022000 -0.86430400 0.03705700
S 0.24472100 -0.25108200 -2.67900900
C 1.13900100 0.88073300 0.40851900
O 2.24371700 -1.42335800 -0.25824400
O 0.18218500 -1.43004900 1.18986900
C -0.19788300 1.48918600 -2.55289000
O -0.70097400 -0.82657700 -3.65828900
O 1.68532700 -0.30061900 -3.00560900
C 0.02615300 1.63020600 0.80195300
C 2.40492700 1.45424100 0.30938700
C -1.55086500 1.82363200 -2.47421100
C 0.79391900 2.46703900 -2.52408500
C 0.19096100 2.98145500 1.09497800
H -0.94946500 1.16019500 0.86586400
C 2.56053500 2.80963600 0.61194400
H 3.24317900 0.84295800 -0.00484600
C -1.91552200 3.16244600 -2.35056000
H -2.29955700 1.04530100 -2.52396800
C 0.41985400 3.80657700 -2.39787500
H 1.83542800 2.17636300 -2.58592800
C 1.45702800 3.57066000 1.00103000
H -0.66662500 3.57785900 1.39078700
H 3.54161400 3.26891000 0.53785600
C -0.92961700 4.15442300 -2.30884800
H -2.96622800 3.43072200 -2.29022800
H 1.18504600 4.57570000 -2.36231600

H 1.57987800 4.62541800 1.22828300
H -1.21452100 5.19756500 -2.20851200
Cu -1.79746200 -2.68764000 -1.14481100
C -4.68336800 -0.20825300 -3.25606000
C -5.01840000 0.68152800 -2.24366100
C -4.51914000 0.47164600 -0.96000100
C -3.67764000 -0.61759700 -0.73744100
C -3.82650300 -1.28631200 -2.98695800
C -3.20891700 -1.00604600 0.61444000
C -3.47777100 -0.26761900 1.76474700
C -3.13659200 -0.81709200 3.00108400
H -3.33774300 -0.26629300 3.91420600
C -2.56817900 -2.08204600 3.05167800
C -2.27324200 -2.76301200 1.86111900
H -5.07880700 -0.08662600 -4.25772300
H -5.67486100 1.52210100 -2.44325000
H -2.33011300 -2.54903700 4.00072700
N -3.32087700 -1.45194400 -1.74687400
N -2.57398500 -2.19885400 0.67829500
C -1.61978000 -4.11109500 1.87468400
H -0.54162000 -3.97571500 1.76295100
H -1.82833600 -4.62294600 2.81737000
H -1.95236000 -4.70600100 1.02231000
C -3.47094600 -2.27408000 -4.05435400
H -2.42281400 -2.14610600 -4.32752700
H -3.57096300 -3.29302600 -3.67741300
H -4.10738300 -2.13378100 -4.93003200
H -3.95596700 0.70175000 1.71029500
H -4.79963200 1.13262500 -0.15078800
F -2.44091200 -5.19975000 -1.06912400
F -1.37288400 -3.59609500 -2.81245100
Cu -0.71231500 -5.16820400 -1.88470900
C 2.84623400 -3.41068100 -3.50501900
C 3.69384400 -3.58286900 -2.42117500
C 3.32058800 -4.43384800 -1.38028500
C 2.07393400 -5.04287900 -1.43879700
C 1.59409600 -4.04750500 -3.51909900
C 1.58339700 -6.02472200 -0.44866500
C 2.38633300 -6.55717200 0.55912200
C 1.85121500 -7.52285100 1.40712300
H 2.45833700 -7.94687400 2.20001900
C 0.54330900 -7.95380800 1.21543600
C -0.22626500 -7.40335200 0.18143700
H 3.12142600 -2.77764000 -4.33996600
H 4.65102700 -3.07326500 -2.38751400
H 0.10570000 -8.71642400 1.84938400
N 1.23939300 -4.80371500 -2.47407300
N 0.30576900 -6.44905300 -0.60463800
C -1.61704400 -7.88835800 -0.10238300
H -2.24110800 -7.05940700 -0.43923900
H -1.58156300 -8.64113800 -0.89769400
H -2.04778900 -8.35723800 0.78478800
C 0.65585700 -3.90549600 -4.67941400
H 0.16672300 -2.92841700 -4.63586700
H 1.22490600 -3.99159900 -5.60836700
H -0.10594200 -4.68253500 -4.63567300
H 3.41380800 -6.23751700 0.67009100
H 3.98588000 -4.60296000 -0.54407800
F -0.31659800 -3.78014100 -0.54142500
F -1.11512300 -6.32623800 -3.25597000
N 3.23031800 -8.82010700 -4.89429600
S 3.68995800 -8.62196400 -3.25016500
S 3.54831500 -7.47514600 -5.93174500
C 2.98023400 -10.05554200 -2.46368000
O 5.15447500 -8.77192000 -3.19305500
O 3.09094700 -7.41566500 -2.65459500
C 5.22488800 -6.97936200 -5.58542800
O 3.49519900 -8.05941300 -7.28325200
O 2.65281100 -6.34837500 -5.62097900
C 3.50267200 -11.32096500 -2.74560200
C 2.00304300 -9.85772600 -1.49070100
C 6.27585200 -7.68624600 -6.17177800
C 5.43402000 -5.89492300 -4.73326100
C 3.00515500 -12.41803200 -2.04586100
H 4.27760600 -11.43943000 -3.49456100
C 1.52210900 -10.96412000 -0.78924200
H 1.63085600 -8.86094300 -1.29812500
C 7.58084600 -7.28883000 -5.88633900
H 6.07239600 -8.51836800 -6.83624400
C 6.74541400 -5.51028300 -4.45858300
H 4.58943900 -5.37894900 -4.29467300
C 2.01734900 -12.23905800 -1.07039000
H 3.39075100 -13.41039900 -2.25596800
H 0.76097300 -10.82501100 -0.02819200
C 7.81371600 -6.20699600 -5.03027900
H 8.41520800 -7.82114100 -6.33168100
H 6.93088600 -4.66887000 -3.79838500
H 1.63796300 -13.09779700 -0.52506600
H 8.83330800 -5.90470200 -4.81128500
C 1.42589500 -9.28735800 -5.29449000
C 0.52261700 -8.50661400 -4.46880700
C -0.35682900 -9.13494700 -3.61824900
C -0.40457500 -10.54324400 -3.54089800
C 0.45949200 -11.33084000 -4.33292100
C 1.35313400 -10.73509800 -5.19099800
H 1.56680200 -8.92819200 -6.31238600
H 0.53954700 -7.42658400 -4.53018600
H -1.02141600 -8.51143300 -3.03298100
H -1.10261200 -11.02538400 -2.86426600
H 0.41301300 -12.41300100 -4.26636900
H 2.01026500 -11.32557200 -5.81972500

D7-N-4F (Benzene, Quintet)
E(BS1) = -5402.658819
E(BS2) = -5406.111765
ZPE = 0.961036
H = 1.036400
G = 0.848570
$<S^2>$ = 6.0477
N -0.14884100 -0.93049700 -1.24019500
S 0.91984400 -0.89494500 0.01924000
S 0.24008900 -0.24488100 -2.68472000
C 1.15643500 0.84439300 0.41171900
O 2.25012200 -1.45352400 -0.29159100
O 0.19789400 -1.47406300 1.16915200
C -0.19438600 1.49554700 -2.53424400
O -0.71341000 -0.80491900 -3.66543600
O 1.67857600 -0.29501400 -3.02060000
C 0.04805300 1.59272500 0.81957700
C 2.42389600 1.41471500 0.31391300
C -1.54515900 1.83525800 -2.44234400
C 0.80205200 2.46856100 -2.50004600

C 0.21886300 2.93966000 1.12853900
H -0.92883900 1.12521800 0.88167300
C 2.58561300 2.76567700 0.63258900
H 3.25860700 0.80447500 -0.01153100
C -1.90290100 3.17411100 -2.30004800
H -2.29770800 1.06104800 -2.49678200
C 0.43500600 3.80811000 -2.35492300
H 1.84177600 2.17399500 -2.57233300
C 1.48656000 3.52560500 1.03619000
H -0.63533900 3.53530400 1.43542700
H 3.56794600 3.22247200 0.55971200
C -0.91222200 4.16102800 -2.25274200
H -2.95195700 3.44633900 -2.22954600
H 1.20398100 4.57324400 -2.31508100
H 1.61415500 4.57704200 1.27587500
H -1.19156900 5.20415500 -2.13774700
Cu -1.81469700 -2.68261500 -1.17597300
C -4.70182500 -0.14408300 -3.20957800
C -5.01784800 0.73071500 -2.17803900
C -4.50553800 0.49479700 -0.90407300
C -3.67092500 -0.60513600 -0.70992000
C -3.85016300 -1.23289900 -2.96923800
C -3.19127500 -1.02191300 0.62975000
C -3.43942600 -0.30133700 1.79584200
C -3.08975800 -0.87606500 3.01826600
H -3.27464500 -0.33945700 3.94319600
C -2.53400700 -2.14743400 3.04007300
C -2.26007400 -2.81047000 1.83438900
H -5.10784400 -0.00210900 -4.20429100
H -5.66970700 1.57980100 -2.35547100
H -2.29020200 -2.63358900 3.97798700
N -3.33182900 -1.42397500 -1.73805800
N -2.56848800 -2.22226100 0.66535100
C -1.62337700 -4.16676700 1.81705300
H -0.54460000 -4.04488400 1.69599000
H -1.82897400 -4.69257900 2.75268400
H -1.97297100 -4.74191800 0.95795800
C -3.51309700 -2.20473600 -4.05692500
H -2.46420700 -2.08706700 -4.33156300
H -3.62693400 -3.22918800 -3.69896400
H -4.15076200 -2.03880300 -4.92717600
H -3.90812900 0.67368800 1.76423400
H -4.77096300 1.14409600 -0.08045200
F -2.48297300 -5.20037400 -1.13259200
F -1.41671800 -3.57773500 -2.86092400
Cu -0.76097600 -5.16698900 -1.95855000
C 2.80307900 -3.41202800 -3.56878700
C 3.65160900 -3.59586900 -2.48757100
C 3.27490000 -4.45030300 -1.45089900
C 2.02567300 -5.05362600 -1.51164400
C 1.54864500 -4.04406100 -3.58599800
C 1.52830300 -6.03292900 -0.52239100
C 2.31900600 -6.55635600 0.49981800
C 1.77276200 -7.51398700 1.34983700
H 2.36912500 -7.92907000 2.15551900
C 0.46870200 -7.95027700 1.14359700
C -0.28676700 -7.41254000 0.09291200
H 3.07984100 -2.77448200 -4.39981500
H 4.61145400 -3.09139000 -2.45195000
H 0.02478900 -8.70921400 1.77755100
N 1.19226600 -4.80708900 -2.54646300
N 0.25372900 -6.46133200 -0.69125700
C -1.66792800 -7.90736700 -0.21750000
H -2.29998200 -7.07442500 -0.52929200
H -1.61367800 -8.62568400 -1.04306800
H -2.09826400 -8.41530900 0.64791400
C 0.60610500 -3.88761800 -4.74066900
H 0.10738600 -2.91672900 -4.67454700
H 1.17186300 -3.94823600 -5.67344400
H -0.14735700 -4.67351300 -4.70901300
H 3.34451400 -6.23449700 0.62160500
H 3.94046600 -4.62772000 -0.61677500
F -0.34424600 -3.79492500 -0.60118100
F -1.16542100 -6.30989900 -3.34081400
N 3.14756300 -8.78402700 -4.88441300
S 3.77261300 -8.63704900 -3.24998100
S 3.47564300 -7.42509200 -5.95537800
C 3.05707900 -10.01379700 -2.37518100
O 5.21675800 -8.87678600 -3.35673200
O 3.29789700 -7.38514000 -2.64203700
C 5.16306200 -6.98415600 -5.61911900
O 3.38408100 -8.01320400 -7.29951600
O 2.60409900 -6.29208700 -5.61456200
C 3.66641200 -11.26815400 -2.48479900
C 1.98341900 -9.77810600 -1.51834000
C 6.18645700 -7.71669300 -6.22311400
C 5.40899300 -5.90691600 -4.76740700
C 3.15089700 -12.32271200 -1.73665200
H 4.51940900 -11.40628400 -3.13907000
C 1.48249600 -10.84421300 -0.77253200
H 1.55088200 -8.78986500 -1.45271800
C 7.50409800 -7.35500100 -5.95164400
H 5.95269600 -8.54216400 -6.88564500
C 6.73354100 -5.55926700 -4.50743100
H 4.58437000 -5.37089000 -4.31568700
C 2.05816200 -12.11133300 -0.88764200
H 3.60225600 -13.30666300 -1.81094600
H 0.64492900 -10.68051200 -0.10275200
C 7.77512100 -6.28278200 -5.09446200
H 8.31874800 -7.90766700 -6.40817300
H 6.94927400 -4.72553800 -3.84693400
H 1.66105200 -12.93806000 -0.30670200
H 8.80495300 -6.00874100 -4.88677300
C 1.70293000 -9.25852400 -5.09707100
C 0.66358500 -8.49559400 -4.32994600
C -0.37067400 -9.14063500 -3.70972800
C -0.46324100 -10.55465600 -3.69151900
C 0.55027000 -11.33260100 -4.30576300
C 1.60592800 -10.74768600 -4.94805800
H 1.57561600 -9.03997400 -6.17167700
H 0.71321100 -7.41472600 -4.32660400
H -1.11649000 -8.52492400 -3.21979600
H -1.28688200 -11.04148000 -3.18003700
H 0.49849000 -12.41636900 -4.24933200
H 2.38732900 -11.33742600 -5.41559900

3-radical cation (Doublet)
E(BS1) = -1091.19617148
E(BS2) = -1091.35961414
ZPE = 0.242044
H = 0.256958
G = 0.200006

$<S^2>$ = 0.7625
C -5.72139000 -2.24911000 0.16675600
C -4.33392100 -2.29745700 0.12494200
C -3.56249100 -1.11873900 -0.01176600
C -4.26594100 0.13801000 -0.01210400
C -5.68215500 0.15751300 0.03607800
C -6.40318900 -1.02173500 0.10756100
C -2.12554200 -1.12712600 -0.07360000
C -3.54183900 1.34165100 -0.00467600
C -2.13795200 1.36088000 -0.03391900
C -1.40983400 0.12064300 -0.10877600
C -0.00669900 0.19595200 -0.28263100
H 0.56528400 -0.70844200 -0.44041100
C 0.65250300 1.41885200 -0.28452500
C -0.05932000 2.62298100 -0.15035400
C -1.43938800 2.59376800 -0.04361200
H -6.28158000 -3.17304700 0.26166900
H -3.84473700 -3.25689500 0.22178100
H -6.19286100 1.11520400 0.02801500
H -7.48674700 -0.99848300 0.14267600
H -4.08312000 2.28283100 0.02399100
H 1.72978200 1.44075800 -0.40889700
H 0.46779600 3.57060700 -0.15698600
H -2.00584800 3.51722900 0.02192600
C -1.37284100 -2.37247800 -0.09186500
C -0.30750300 -2.71540100 0.72958200
S -1.67691100 -3.66106300 -1.25452000
C 0.24028600 -3.98771800 0.44402300
H 0.03186400 -2.07517300 1.53463600
C -0.38364700 -4.60558100 -0.61434900
H 1.06347900 -4.42953200 0.99131700
H -0.14657100 -5.56186100 -1.06111500

4-radical cation (Doublet)
E(BS1) = -1028.78374087
E(BS2) = -1028.94663092
ZPE = 0.187015
H = 0.200352
G = 0.146006
$<S^2>$ = 0.7646
C 0.34436300 0.15872800 0.00002000
C 1.76026700 0.16922800 0.00012000
C 1.06703800 2.19447800 0.00007000
O -0.08632600 1.42085100 -0.00001500
H -0.40254400 -0.62047800 -0.00003100
N 2.16600300 1.46939400 0.00015200
C 2.66068200 -0.95713800 0.00018000
C 4.05646800 -0.73175200 0.00022300
C 2.16720700 -2.28311900 0.00019400
C 4.93132800 -1.80701300 0.00027700
H 4.42695500 0.28672200 0.00021200
C 3.05166600 -3.35187100 0.00024600
H 1.09992000 -2.47452700 0.00016600
C 4.43273300 -3.11789800 0.00028800
H 6.00198200 -1.63370500 0.00031100
H 2.67178400 -4.36759600 0.00025500
H 5.12119200 -3.95679100 0.00033000
C 0.92192400 3.59420200 0.00005700
C -0.27390400 4.32326100 0.00011800
S 2.32371000 4.64759300 -0.00004700
C -0.05324000 5.70599100 0.00008500
H -1.24733900 3.84951200 0.00018200
C 1.29738400 6.02617100 0.00000100
H -0.83818900 6.45097400 0.00012400
H 1.73457000 7.01624600 -0.00003100
